# Exploring Novel Indazole and Pyrazole Nucleoside Analogues: Enzymatic Synthesis, Characterization, and Biological Evaluation

**DOI:** 10.3390/ijms27177551

**Published:** 2026-08-24

**Authors:** Anton F. Mironov, Barbara Z. Eletskaya, Konstantin V. Antonov, Alexander S. Paramonov, Roman S. Esipov, Irina D. Konstantinova, Sofya N. Andreevskaya, Tatiana G. Smirnova, Inna L. Karpenko, Vera A. Sokhraneva, Iulia S. Zhivotova, Sergey N. Kochetkov, Anastasia L. Khandazhinskaya, Elena S. Matyugina

**Affiliations:** 1 Shemyakin-Ovchinnikov Institute of Bioorganic Chemistry, Russian Academy of Sciences, Miklukho-Maklaya St. 16/10, 117997 Moscow, Russia; anton-mironov1999@inbox.ru (A.F.M.); fraubarusya@gmail.com (B.Z.E.); antonov.kant@yandex.ru (K.V.A.); a.s.paramonov@gmail.com (A.S.P.); esipov@ibch.ru (R.S.E.); kid1968@yandex.ru (I.D.K.); 2 Institute of Pharmacy and Biotechnology, Peoples’ Friendship University of Russia Named after Patrice Lumumba, Miklukho-Maklaya St. 6, 117198 Moscow, Russia; 3 Microbiology Department, Central Tuberculosis Research Institute, 2 Yauzskaya Alley, 107564 Moscow, Russia; andsofia@mail.ru (S.N.A.); s_tatka@mail.ru (T.G.S.); 4 Engelhardt Institute of Molecular Biology, Russian Academy of Sciences, Vavilov St. 32, 119991 Moscow, Russia; ilkzkil@gmail.com (I.L.K.); sokhraneva.v@mail.ru (V.A.S.); zhivotovajulia51@gmail.com (I.S.Z.); snk1952@gmail.com (S.N.K.); khandazhinskaya@bk.ru (A.L.K.)

**Keywords:** indazole, pyrazole, nucleoside analogues, purine nucleoside phosphorylase, antibacterial activity, antituberculosis activity

## Abstract

New indazole and pyrazole nucleoside analogues were synthesized by enzymatic transglycosylation using *E. coli* purine nucleoside phosphorylase (PNP). Indazoles substituted at position 5 or 6 with bromine, pyrazole or pyrimidine fragments were used as heterocyclic bases. In the case of indazole-pyrazole hybrids, the selectivity of PNP in glycosylation of the pyrazole fragment, rather than the indazole one, was established. Molecular modeling methods allowed the identification of the preferred orientations of substrates in the active site of the enzyme, leading to the formation of N1- and N2-regioisomers of indazole. Unique substrate specificity ensured the possibility of glycosylation of the bases with conversion rates of 65–100% (according to HPLC analysis of the reaction mixtures) and isolated yields of 19–94%. It was shown that the synthesized nucleoside analogues are not inhibitors of *E. coli* adenosine deaminase (ADA). They do not exhibit cytotoxicity towards cancer cell lines (SH-SY5Y neuroblastoma cell lines, K-562 lymphoblastic cells, and HL-60 promyeloblastic cells). The activity of indazole derivatives against *Mycobacterium tuberculosis* (strain H37Rv) was determined by the microdilution method. 1-(β-D-2′deoxyribofuranosyl)-5-bromo-indazole and 5-(pyrimidin-5-yl)-1H-indazole at a concentration of 50 μg/mL were able to inhibit 50% and 90% of the culture growth, correspondingly.

## 1. Introduction

**Figure 1:**
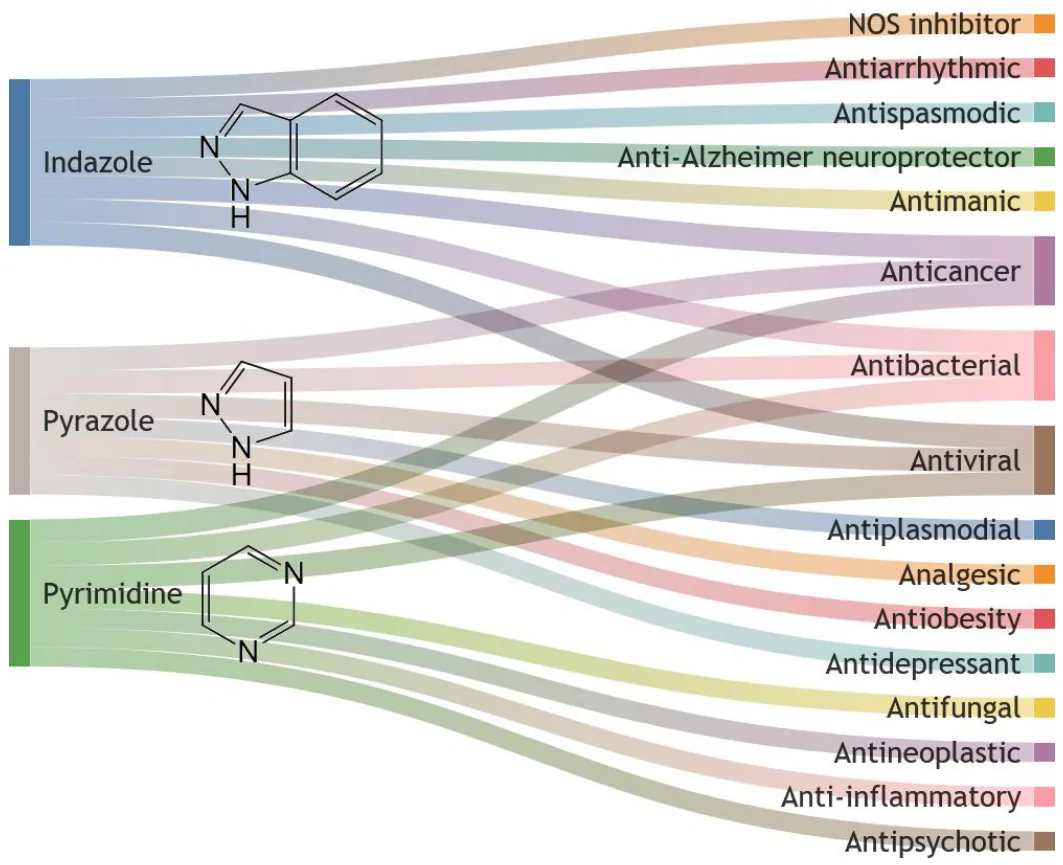
Biological activity of indazole, pyrazole and pyrimidine derivatives.

The need to introduce novel effective antimetabolites and nucleoside antibiotics into clinical practice has driven a considerable interest in the synthesis of compounds containing heterocyclic bases that are structurally distinct from natural purine and pyrimidine bases (e.g., substituted indazoles and pyrazoles). Derivatives of such bases are of notable interest due to the broad spectrum of activity they exhibit (Figure 1) [1,2,3].

Indazole derivatives pazopanib (inhibits VEGF-induced phosphorylation of vascular endothelial growth factor receptor-2 (VEGFR-2), angiogenesis) [4] and niraparib (poly[adenosine diphosphate ribose] polymerase inhibitor) [5] have found application in oncology. The indazole core integrated into a heterocyclic system is capable of interacting with biological targets, for example, inhibiting nitric oxide synthase (NOS), which can be used to treat septic shock and slow tumor growth [6,7]. New indazole derivatives were intensively synthesized for study of their antimicrobial activity [8,9,10].

Pyrazole, as well as indazole, is one of the key pharmacophore groups in the design of new chemotherapeutic agents [11]. Clinically approved drugs include the anti-inflammatory celecoxib [12] and diphenamisole [13] and the antibacterial ceftolozane [14]. Betazole, an H2-receptor agonist with bactericidal activity, is also noteworthy [15].

Pyrimidine derivatives form a large group of antitumor (nilotinib [16], imatinib [17], osermitibinib [18], pralsetinib [19]), antiviral (etravirine [20], rilprivirine [21]), antibacterial (trimethoprim [22], iclaprim [23]), and anti-inflammatory (epirizole [24]) drugs.

**Figure 2:**
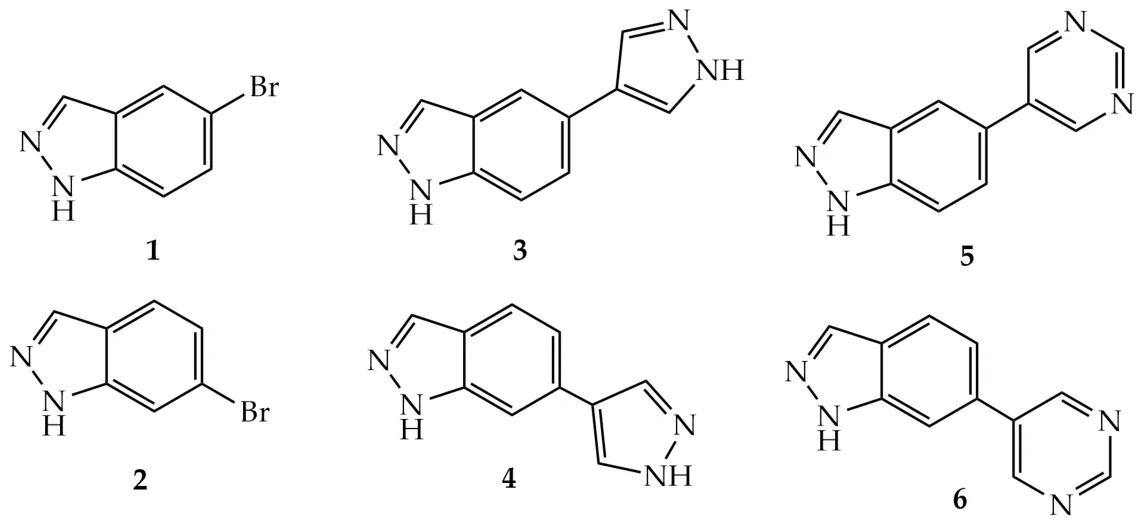
Indazole derivatives substituted at positions 5 and 6.

Previously, we obtained the corresponding pyrazole-indazole and pyridine-indazole derivatives (**3**–**6**) from bromoindazoles (**1**, **2**) (Figure 2). Among them, 6-(1H-pyrazol-4-yl)-1H-indazole (**4**) showed antiviral activity against the influenza A virus and antitumor activity, and 5-(pyrimidin-5-yl)-1H-indazole (**5**) exhibited antibacterial properties [9].

Conjugation of heterocyclic bases with carbohydrate residues changes the properties of the molecule, including solubility and intracellular metabolism. This often creates the possibility for obtaining compounds with enhanced or fundamentally new activity realized within the nucleoside/nucleotide metabolic pathway [25]. Inclusion in nucleoside/nucleotide metabolism may also weaken the cytotoxicity or antiviral activity of the resulting nucleoside compared to the original base. In this case, it can be assumed that the modified heterocyclic base exhibits its biological activity without undergoing intracellular conversion to the corresponding nucleoside. Thus, the preparation of nucleoside analogs and comparison of their biological properties with those of the original bases **1**–**6** can contribute to an understanding of the mechanism of action.

For the synthesis of pyrazole nucleosides, classical chemical methods [26,27,28,29] and modern biocatalytic methods based on the enzymatic transglycosylation reaction can be used. In the latter case, the possibility of obtaining new derivatives is limited by the substrate specificity of bacterial nucleoside phosphorylases. *E. coli* nucleoside phosphorylases (*E. coli* PNPs) have broad substrate specificity. It is known that, except for substituted purine bases, as substrates, *E. coli* PNPs accept fleximer bases, pyrazolo-pyrimidines, and tricyclic conjugated systems [30,31].

Here, we present the synthesis of new nucleoside analogues containing bromoindazole, indazole-pyrazole, or indazole-pyrimidine fragments as a heterocyclic base. Antitumor and antibacterial properties have previously been described for these analogs [9]. Despite the high interest in modified nucleosides, the conjugates we propose remain poorly studied, which underscores the relevance of this study. Based on the obtained heterocycles, ribo- and 2′-deoxyribonucleosides were synthesized for the subsequent evaluation of cytotoxicity against several cancer cell lines, as well as inhibitory activity against *E. coli* adenosine deaminase and antibacterial activity against *Mycobacterium tuberculosis* (strain H37Rv).

## 2. Results and Discussion

### 2.1. Enzymatic Synthesis of Nucleosides

In this study, 5-Bromo-**1** and 6-bromoindazole **2** were used for the synthesis of indazole derivatives **3**–**6** (Figure 2) by means of the Suzuki–Miyaura cross-coupling reaction, as described previously [9]. It should be noted here that 5-bromo-1-(β-D-ribofuranosyl)indazole was previously obtained using the Vorbruggen method, which showed moderate cytotoxicity against tumor cells (U-937, K-562, Raji) [26].

The possibility of enzymatic synthesis of ribo- and 2′-deoxyribofuranosides, bearing compounds **1**–**6** as heterocyclic bases, was investigated using *E. coli* PNP obtained earlier [32]. Pyrazole-containing fleximer bases are known to be good substrates for *E. coli* PNP [33,34]. In was shown that imidazole-indazoles are good substrates for *L. leichmannii* nucleoside deoxyribosyltransferase (*L*NDT) [35]. Vichier-Guerre S. et al. carried out the enzymatic synthesis of 2′-deoxynucleoside using *L*NDT, in which 5-(4-imidazolyl)-1H-indazole was primarily glycosylated at the imidazole moiety, and the indazole moiety was subjected to secondary glycosylation. In the synthesis of riboside with 5-(4-imidazolyl)-1H-indazole using *E. coli* PNP, one product was formed at the imidazole moiety [35]. It can be expected that due to the similarity of the catalytic reactions carried out by *L*NDT and *E. coli* PNP, indazoles **1**–**6** may turn out to be substrates for *E. coli* PNP. Moreover, the active center of *E. coli* PNP accepts more complex tricyclic ethenoadenine bases as substrates [36]. The literature data state that *E. coli* PNP probably exhibits extremely low substrate activity towards indazole [37]. However, there is currently no practical evidence using *E. coli* PNP for glycosylation directly at the indazole fragment.

**Figure 3:**
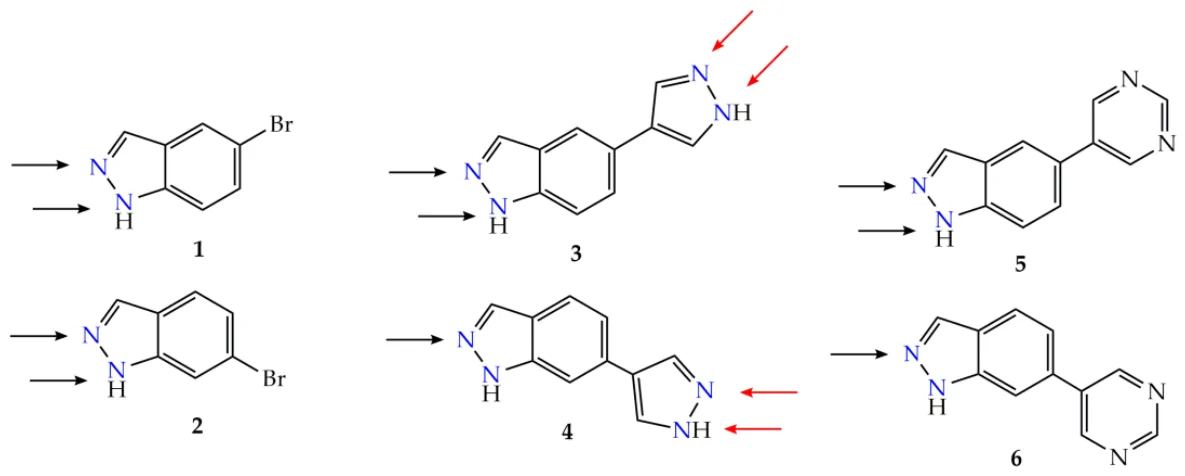
Potential glycosylation sites of indazole bases by *E. coli* PNP. Nitrogen atoms, the potential glycosylation sites, are shown in blue; black arrows indicate the predominant reaction direction, and red arrows indicate secondary directions.

The possibility of forming several regioisomeric nucleosides in the active center remains for indazole systems with a pyrazole substituent. Glycosylation of the starting 5(6)-bromoindazoles (**1**, **2**) can occur at both the N1 and the N2 atoms (Figure 3).

The tautomerism of the indazole nucleus, which can exist in the form of 1H- and 2H-tautomers, explains the formation of a mixture of regioisomers, and the 1H-form predominates in the solution [38]. Theoretically, glycosylation of pyrazole-indazole bases **3** and **4** at all four nitrogen atoms is possible. In the case of indazole-pyrimidines **5** and **6**, glycosylation can only occur at the nitrogen atoms of the indazole. Due to steric hindrance, the N1-regioisomer of the nucleoside of base **5** and the N2-regioisomer of the nucleoside of base **6** should predominate.

**Figure 4:**
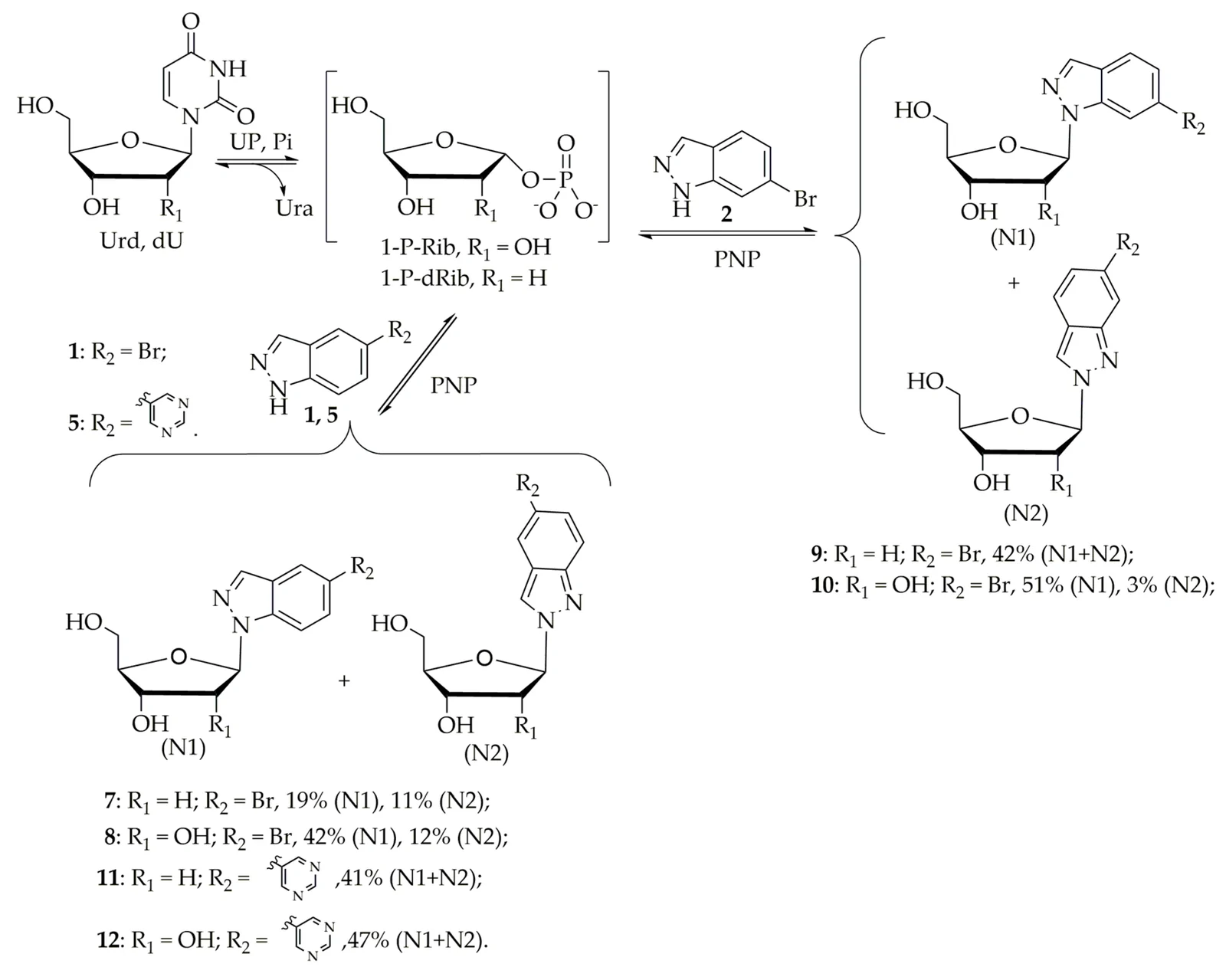
Enzymatic synthesis of nucleoside analogues at the indazole moiety.

Good substrate properties for all indazole derivatives, with the exception of base **6,** were shown in the *E. coli* PNP reaction. The transglycosylation reaction (Figure 4) for 5-bromo-indazole (**1**) proceeded with the formation of two products: for the riboside, the N1-isomer (N1-(**8**), 77%) predominated over the N2-isomer (N2-(**8**), 23%), whereas for 2′-deoxyriboside the ratio was N1-(**7**) (64%) and N2-(**7**) (36%). For 6-bromo-indazole (**2**), in the riboside series, a predominance of the N1-isomer (N1-(**10**), 94%) over N2-(**10**) (6%) was observed, and among 2′-deoxyribosides, the ratio was N1-(**9**) (79%) and N2-(**9**) (21%). The isomer ratios in the reaction mixture of nucleoside synthesis are given according to high-performance liquid chromatography (HPLC) data. For the pair of N1-2′-deoxyribo-6-bromo-2H-indazole and N2-2′-deoxyribo-6-bromo-1H-indazole (N1-(**9**) and N2-(**9**)) the isomer ratios were determined by NMR spectra. All products were successfully isolated, with the exception of isomers N1-(**9**) and N2-(**9**), which could not be separated under the conditions used without a significant loss of yield. The formation of a mixture of regioisomers is consistent with previously described cases of non-regioselectivity of the enzymatic glycosylation of unnatural bases catalyzed by bacterial PNP [34]. For example, in the case of benzimidazole nucleosides with asymmetric substituents using *E. coli* PNP, N1 and N3 nucleoside regioisomers are formed [39].

In the case of 5-pyrimidinyl-1H-indazole glycosylation (**5**), maximum conversion to ribo- and 2′-deoxyribosides was not achieved (Table 1). The formation of minor N2-regioisomeric nucleosides was observed (Figure 4). Due to the low yields of the obtained minor nucleosides, 3% for riboside and 6% for 2′-deoxyriboside, according to HPLC data (Figures S62 and S63), they were not isolated from the reaction mixture. In the synthesis of nucleoside analogue N1-(**12**), the minor N2-regioisomer (N2-(**12**)) was detected as an impurity of the N1-regioisomer when recording the NMR spectrum (Figures S33–S35).

**Figure 5:**
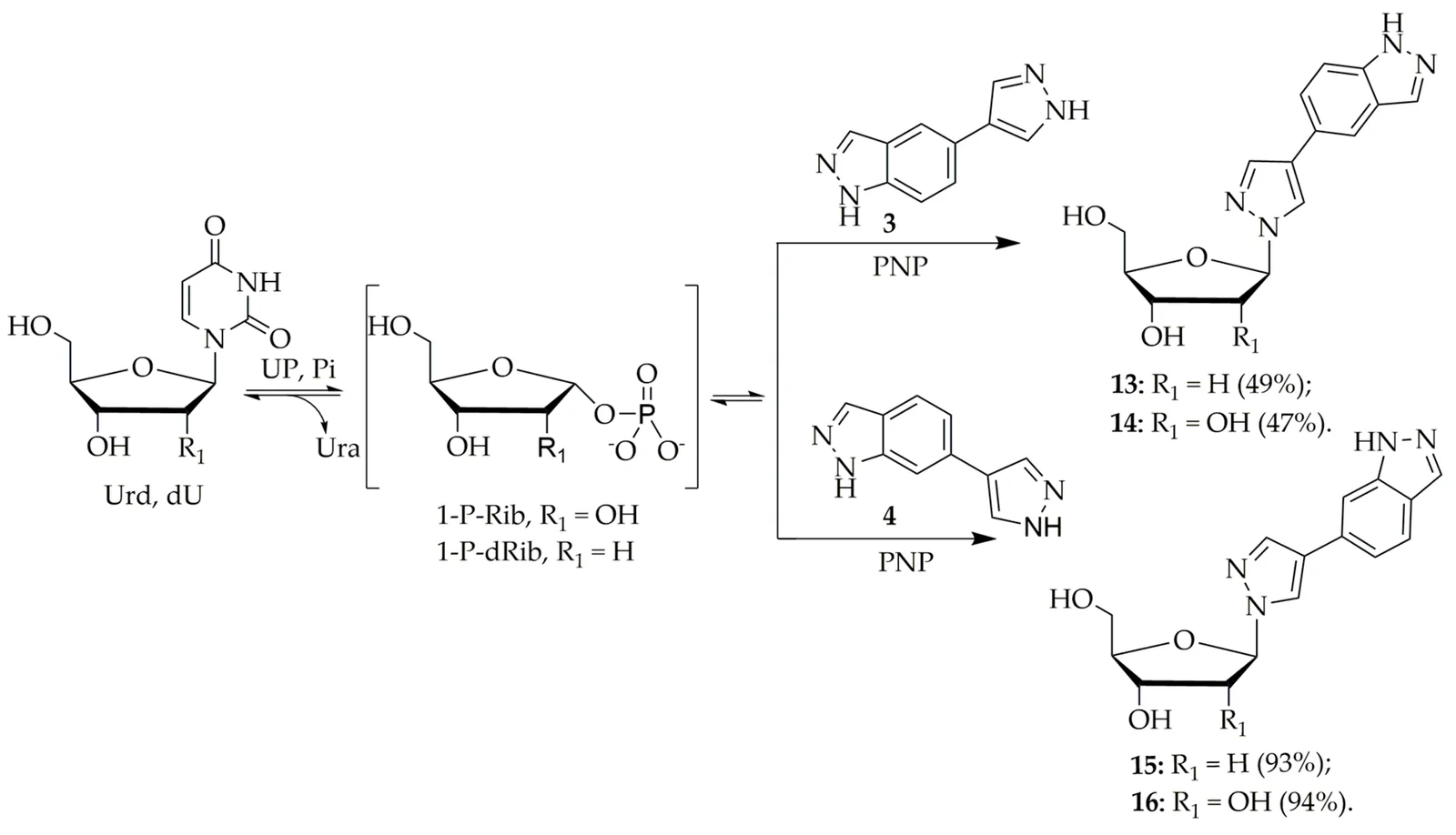
Enzymatic synthesis of nucleoside analogs at the pyrazole moiety.

Indazole derivative **6** did not exhibit substrate properties for *E. coli* PNP according to HPLC and HRMS data (Figures S64 and S65), although we hypothesized that the N2 atom of indazole might be glycosylated. According to chromatographic and mass spectrometric analysis (Figures S58–S61), glycosylation of bases **3** and **4** leads to only one product in each case. Due to the structural features of the hybrid compounds **3** and **4** and the possibility of glycosylation of both the indazole and pyrazole fragments, we expected the formation of a mixture of isomers. However, the experiment revealed absolute regioselectivity of the reaction directly in the pyrazole center (Figure 5).

Structures of N1- and N2-regioisomers of indazole nucleosides were characterized by nuclear magnetic resonance (NMR) spectroscopy (Figures S1–S53) and high-resolution mass spectrometry (HRMS). The reaction mixtures were analyzed by HPLC (Figures S54–S64). The quantitative ratio of N1- and N2-2′-deoxyribosides of 6-bromoindazole (N1-(**9**) and N2-(**9**)) in the unseparated mixture was determined by NMR spectroscopy. The conditions of the enzymatic synthesis of nucleosides **7**–**16** are presented in Table 1. The physicochemical characteristics, NMR and HRMS spectra, and HPLC profiles of the synthesized nucleosides are given in the Supplementary Information.

Thus, despite the significant differences between 5(6)-substituted indazoles and natural purines, bases **1**–**5** proved to be effective substrates of *E. coli* PNP. The nucleosides were synthesized with good conversion according to HPLC data and moderate yields of isolated products. The formation of N2-regioisomeric products complicated the isolation of the target N1-regioisomeric indazole nucleosides. Experiments in vitro were performed with individual N1-isomers of nucleosides, but in the case of (β-D-2′-deoxyribofuranosyl)-6-bromo-indazole (**9**) and (β-D-ribofuranosyl)-5-(pyrimidin-5-yl)-indazole (**12**) there was a mixture of isomers (N1-(**9**)/N2-(**9**)—79/21%, N1-(**12**)/N2-(**12**)—94/6%).

### 2.2. Molecular Docking and Partial Charge Analysis

**Figure 6:**
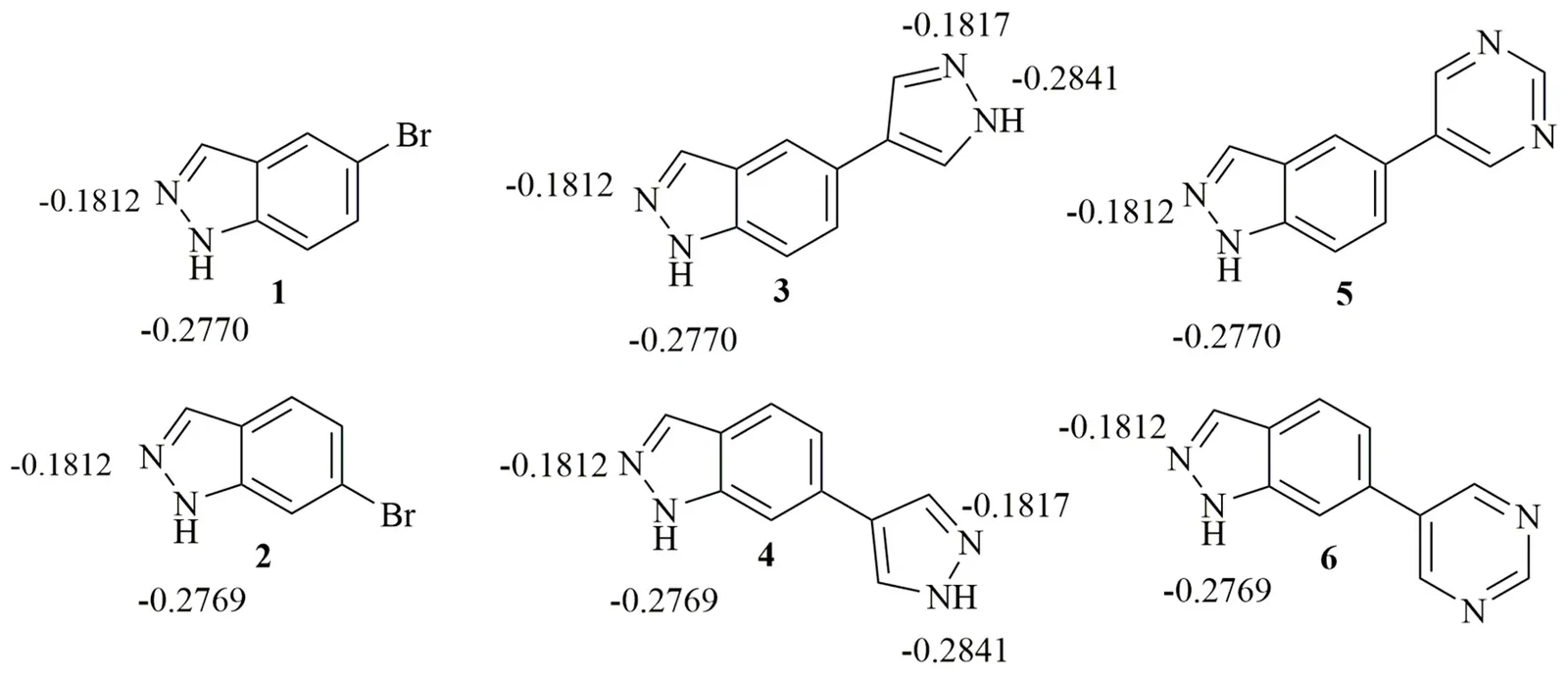
Semi-empirical AM1-BCC partial atomic charges calculated for nitrogen atoms in the investigated compounds. The most negative charge indicates the preferred site for glycosylation.

To understand why some of the studied bases are not substrates of the enzyme, we hypothesized that the substituents might influence the charge distribution on the nitrogen atoms involved in the glycosylation reaction. To test this hypothesis, we calculated the AM1 BCC charges (Austin model 1 with bond charge correction) (Figure 6) for the nitrogen atoms in compounds **1**–**6**.

The results showed that the N1 atom of indazole has a greater negative charge (−0.2770 e for **1** and −0.2769 e for **2**) than the N2 atom (−0.1812 e for both compounds), which should determine the reaction predominantly at the N1 position. Despite this, N2 isomers of indazole were also experimentally confirmed in the reaction mixture.

For pyrazole-indazoles, the charge for the N1 atom of the pyrazole fragment is −0.2841 e for compounds **3** and **4**, which is slightly higher (more negative) than that of the N1 indazole fragment (−0.2770 e for **3** and −0.2769 e for **4**). From an electronic point of view, this indicates that the transglycosylation reaction should proceed predominantly at the pyrazole fragment, which is completely consistent with the experimental data. The absence of *bis*-glycosides in the reaction mixture is apparently due to the steric inaccessibility of the second site after the first glycosylation event. A similar situation was described in [35] for imidazole-indazole, where the formation of secondary products was also not observed. This suggests that the active site of *E. coli* PNP does not allow sequential glycosylation at two heterocycles within a single substrate.

Analysis of AM1 BCC charges does not reveal significant differences between 5-(pyrimidin-5-yl)-1H-indazole (**5**) and 6-(pyrimidin-5-yl)-1H-indazole (**6**). The electronic properties of nitrogen atoms in both compounds and charges on the N1 and N2 atoms of indazole (for the substrate (**5**), N1 is −0.2770 e and N2 −0.1812 e; for the non-substrate (**6**), N1 is −0.2769 e and N2 −0.1812 e) are virtually identical. So, the lack of glycosylation for compound **6** is due to steric rather than electronic factors—the bulky pyridyl substituent at position **6** probably creates steric hindrances during binding in the enzyme’s active site.

To elucidate the reasons for such different glycosylation reaction and byproduct formation, molecular docking of the studied compounds to the active site of *E. coli* PNP was performed. The primary goal was to assess whether steric or conformational factors prevent the correct orientation of the ligand within the active site and whether these factors could explain the absence of products for some indazole derivatives, as well as the regioselectivity of the formation of N1- and N2-isomers.

The following criteria were used to determine the correct orientation of the ligand in the active site: the distance from the nitrogen atoms of the base to the Ser90 residue (hydrogen bond) [40], the angle between the plane of the heterocycle and the aromatic ring of Phe159, and the distance from the centroid of the base to the plane of Phe159 (π–π stacking). The results are presented in Table 2.

Docking results showed that all the studied compounds exhibit affinity for the active site of the *E. coli* PNP protein in the binding energy range from −5.49 to −7.27 kcal/mol. No correlation was observed between the binding energy and substrate activity; geometric parameters were decisive. For all active substrates (+positions), the distance to Ser90 was 2.87–3.25 Å, the angle with Phe159 was 48–61°, and the distance from the centroid of the base to the plane of Phe159 did not exceed 0.94 Å. Inactive conformations (−positions) did not meet these criteria.

**Figure 7:**
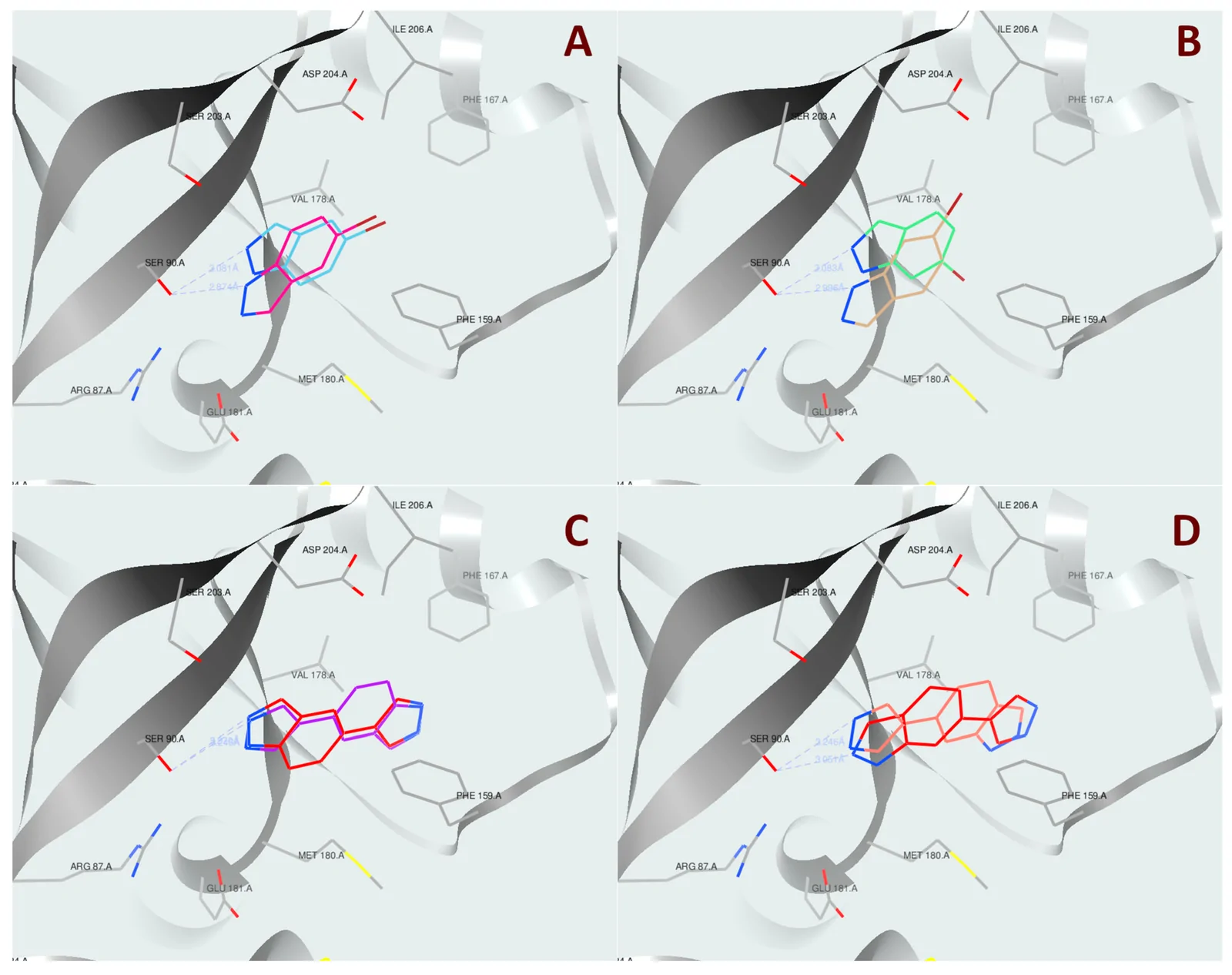

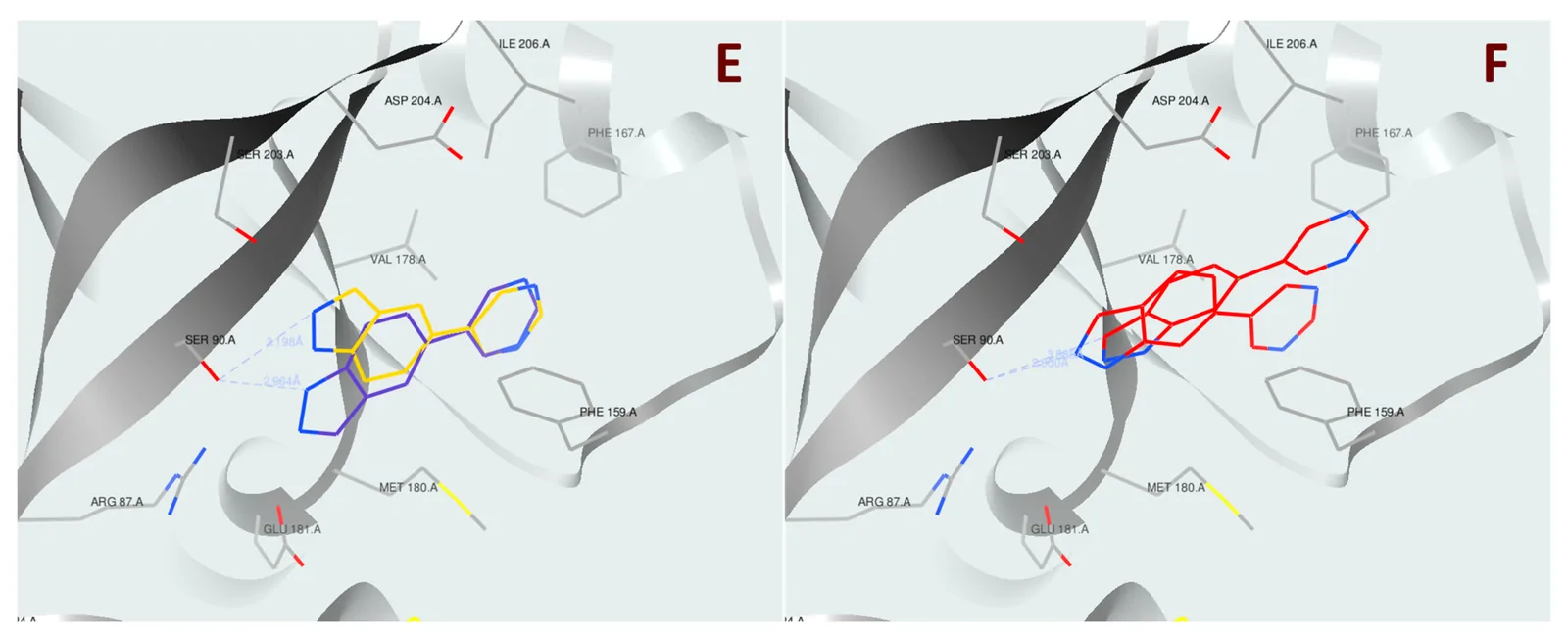
Molecular docking of the studied compounds in the active site of *E. coli* PNP: (**A**) 5-bromo-1H-indazole (**1**)—glycosylation model at N1 (blue) and N2 (pink) atoms; (**B**) 6-bromo-1H-indazole (**2**)—similar glycosylation model at N1 (light green) and N2 (beige); (**C**) 5-(1H-pyrazol-4-yl)-1H-indazole (**3**)—similar glycosylation model at the pyrazole fragment (orange) and at the indazole fragment (red, not realized experimentally); (**D**) 6-(1H-pyrazol-4-yl)-1H-indazole (**4**)—glycosylation model at the pyrazole fragment (purple) and at the indazole fragment (red, not supported by experimental data). Hydrogen bonds with Ser90 are shown as blue dotted lines; distances (Å) are indicated. Molecular docking of the studied compounds in the active site of *E. coli* PNP. (**E**) 5-(pyrimidin-5-yl)-1H-indazole (**5**)—glycosylation model at the N1 (yellow) and N2 (purple) atoms of indazole; (**F**) 6-(pyrimidin-5-yl)-1H-indazole (**6**)—glycosylation model at the N1 and N2 atoms of indazole (red) (predicted by docking, but not confirmed by experimental data). Hydrogen bonds with Ser90 are shown with blue dotted lines; distances (Å) are indicated.

Structures of indazole derivatives (**1** and **2**) in the active site of *E. coli* PNP are shown in Figure 7 (models A and B). Binding of the indazole ring can occur through two different orientations, creating conditions for glycosylation at both the N1 and N2 nitrogen atoms. The presented arrangements illustrate the mechanism of formation of regioisomeric nucleosides.

The docking results for indazole-pyrazole conjugates **3** and **4** are shown in Figure 7 (models C and D). There are two possible orientations: glycosylation at the pyrazole moiety (experimentally observed) and at the indazole moiety (theoretical, not observed experimentally). For compounds **3** and **4**, the lack of glycosylation at N1 of the indazole is due to the distance from the centroid of the indazole base to the Phe159 plane being outside the acceptable range (0.861–1.201 Å, with a norm of <0.94 Å), while the angle (47.3–48.9°) is only at the lower limit of acceptable values. Figure 7 (models E and F) shows the docking results for pyrimidyl indazoles **5** and **6**, which, despite their structural similarity, exhibit different specificities for the active site of *E. coli* PNP.

The key difference between compounds **5** and **6** is the position of the hydrophobic substituent (5th vs. 6th), which determines substrate specificity. For indazole derivative **5**, both orientations of the base in the active site satisfy the geometric criteria characteristic of the substrates, which ensures the formation of products at both the N1- and N2-nitrogen atoms (model E). For compound **6**, the substituent is in the 6-position, which facilitates its interaction with the hydrophobic cavity formed by the residues Phe159, Phe167, Ile206, and Val178 (Model F). Additional hydrophobic contacts stabilize the complex; however, this is accompanied by a shift of the indazole core relative to the optimal position in the active site, which is manifested by an increase in the distance to Ser90 (>3.8 Å), a decrease in the angle with Phe159 (38–48°), and an increase in the distance to the Phe159 plane (>1.1 Å). The combination of these disturbances explains the lack of substrate activity in compound **6**, despite the close binding energy values of the studied compounds.

### 2.3. Cell Line In Vitro Tests

Earlier anticancer activity against promyeloblastic cells HL-60, lymphoblastic cells K-562 and other cell lines was established for compounds **3** (IC_50_ 15.5–36.8 µM) and 4 (IC_50_ 4.2–9.9 µM) [9] and compound **8** [26]. Compounds **7**–**16** were screened for cytotoxicity against the above-mentioned cell lines as well as neuroblastoma cells SH-SY5Y. Compound **8** exhibited cytotoxicity against K-562 at the level described in the literature (IC_50_ = 100 µM), while no activity was observed for derivatives **7** and **9**–**16** (IC_50_ > 100 µM). Compounds **5**, **7**, and **10**–**12** were also tested against epithelial human liver cells THLE-2; no toxic effect was observed (IC_50_ > 100 µM).

### 2.4. Antituberculosis Studies

The antimycobacterial activity of compounds **1**, **2**, **5**, **7**, **8** and **10**–**12** was assessed on the laboratory strain of *M. tuberculosis* (MTb) H37Rv (Table 3), with rifampicin as a positive control and untreated cultures as a negative control.

Screening of the anti-tuberculosis activity showed that compounds **5**, **7**, and **12** possessed a moderate inhibition. Different patterns were observed for bromo-indazole and pyrimidinyl-indazole derivatives. In case of bromoindazoles, glycosylation to corresponding nucleoside analogs led to the appearance of antimycobacterial activity. Starting bromoindazoles **1** and **2** were not active. 2′-Deoxynucleoside **7**, containing 5-bromoindazole **1**, was more active than the corresponding ribonucleoside **8** (99.55% versus 55.60% at 100 μg/mL). Ribonucleoside **10**, based on 6-bromoindazole **2**, inhibited the growth of culture on 69.94% at 100 μg/mL.

In the case of 5-(pyrimidin-5-yl)-1H-indazole (5) and its nucleoside derivatives, we observed the opposite effect, i.e., a decrease in activity. Among the 5-pyrimidinyl-1H-indazole derivatives, base **5** demonstrated the highest activity, with 99.40% inhibition of culture growth at a concentration of 100 μg/mL and 91.86% at a concentration of 50 μg/mL, followed by ribonucleoside **12** and the corresponding 2′-deoxyribonucleoside **11**. Obviously, 5-(pyrimidin-5-yl)-1H-indazole **5** does not need the glycosylation step to inhibit mycobacterial growth. This led us to speculate that the main mechanism of action of these derivatives does not involve nucleoside metabolism.

### 2.5. The ADA Inhibition Test

The development and progression of pulmonary tuberculosis is always accompanied by dysfunction of the immune system. Key enzymes of purine metabolism adenosine deaminase (ADA) and its isoenzymes, which control adenosine levels, play an important role in the regulation of cellular immunity [41]. Modified nucleosides can reduce the activity or even inhibit this enzyme [42]. Therefore, during the study of properties of new nucleoside analogues, ADA activity is usually screened. The ability to inhibit *E. coli* ADA was studied in the presence of bases **1**–**6** and nucleosides **7**–**16**. Adenosine was used as a negative control for testing ADA activity, and cladribine (Ki 0.2 μM) and pentostatin (Ki 2.5 pM) [42] were used as a positive control of inhibition. Then, adenosine was deaminated with the addition of the obtained substances. The synthesized compounds at a concentration of 0.1 mM had an insignificant effect on the enzymatic activity of *E. coli* ADA. They reduced the rate of adenosine deamination within 10–15%. Among all tested compounds, nucleoside **13** reduced the rate of adenosine deamination by 27%, while cladribine reduced the rate by 44%, and pentostatin completely inhibited the reaction. The effects of indazole bases **1**–**6** and nucleosides **7**–**16** on ADA activity are given in the Supplementary Information.

## 3. Materials and Methods

### 3.1. General Procedures

All chemicals and solvents were of laboratory grade and obtained from commercial suppliers and were used without further purification. The method of producing recombinant *E. coli* phosphorylases was described earlier. The following recombinant *E. coli* [32] enzymes were used in the present study: uridine phosphorylase (UP) with a specific activity of 00 units per mg of protein, 9 mg per mL; purine nucleoside phosphorylase (PNP), 52 units per mg, 15 mg per ml. The production of adenine deaminase from *E. coli* and the determination of enzymatic activity were described previously [43]. Analytical HPLC was performed on the Waters system (Waters 1525, Waters 2489, Breeze 2, Waters Corporation, Milford, MA, USA); (a) Nova Pak C18 (Waters Corporation, Milford, MA, USA), 4.6 × 150 mm, eluent A—0.1% TFA/H_2_O, eluent B—70% acetonitrile in 0.1% TFA/H_2_O, flow rate 1 mL/min, detection at 280 nm, gradient 0–100% B, 20 min; (b) Ascentis^®^ Express C18 column (Supelco, Bellefonte, PA, USA). 2.7 μm 7.5 × 3.0 mm, eluent A—0.1% TFA/H_2_O, eluent B—70% acetonitrile in 0.1% TFA/H2O, flow rate 0.5 mL/min, detection at 280 nm, gradient 0–100% B, 20 min. NMR spectra were recorded using a Bruker Avance II 700 spectrometer (Bruker BioSpin, Rheinstetten, Germany) in DMSO-d6 at 30 °C. Chemical shifts in ppm (δ) are measured relative to the residual solvent signals as internal standards (2.50). Coupling constants (J) are measured in Hz. UV–Spectra were recorded using a Hitachi U-2900 spectrophotometer (Hitachi High-Tech Corporation, Tokyo, Japan).

High-resolution mass spectra were recorded using a micrOTOF-Q II device (Bruker Daltonics, Bremen, Germany) by electrospray ionization mass spectrometry (ESI-MS). The following parameters were used: capillary voltage 4500 V; mass scanning range *m*/*z* 50–2000; external calibration with Agilent Calibrant Solution (Agilent, Darmstadt, Germany); gas pressure 0.4 bar; nitrogen spray gas (4 L/min); interface temperature 180 °C. The samples were injected into the mass-spectrometer spray chamber from an Agilent 1260 HPLC chromatograph equipped with an Agilent Poroshell 120 EC-C18 column (3.0 × 50 mm; 2.7 μm) (Agilent Technologies Inc., Santa Clara, CA, USA) and a compatible pre-column cartridge using an autosampler. The samples were eluted in the acetonitrile gradient in 0.1% formic acid with a flow rate of 300 µL/min. Molecular ions in the spectra were analyzed and matched with the appropriately calculated *m*/*z* and isotopic profiles in the Bruker Data Analysis 4.0 program. Samples were dissolved in mQ H_2_O.

Compounds **3**–**6** were prepared as described earlier [9].

### 3.2. Enzymatic Reactions

The reaction conditions of nucleoside synthesis (ratio of reagents, PNP amount, reaction time, and conversion of the base into nucleoside) are provided in Table 1. Uridine or 2′d-uridine and KH_2_PO_4_ were dissolved in water at 40–50 °C, pH was adjusted up to 7.0, and bases **1**–**5** were dissolved in 1 mL of DMF and added to the reaction mixture. The enzymes (PNP, UP) were added, and the reaction mixtures were incubated at 50 °C. Reaction progress was monitored by HPLC. When conversion reached the highest value, reaction was terminated by the addition of ethanol (50%, *v*/*v*), and sharp cooling interrupted the reaction. The reaction mixtures were evaporated up to 5 mL. The products were purified by reverse-phase column chromatography (silica gel C18, Merck, Darmstadt, Germany), with a 145 × 15 mm column. Nucleosides **11**–**16** were eluted from the column with a gradient of 50% ethanol in water (500 mL, flow rate 1 mL/min). For nucleoside **11**, additional column chromatography was performed on silica gel 60 (Fluka, Darmstadt, Germany), with a 100 × 10 mm column and a gradient elution system of chloroform—chloroform:methanol (9:1, 500 mL, flow rate 1.0 mL/min).

For nucleosides **7**–**10**, the products were purified by reversed-phase column chromatography (silica gel C18, Merck, Darmstadt, Germany), with a 145 × 15 mm column and a gradient elution system of 20% methanol in water—100% methanol. For the separation of the N1 and N2 isomers, a preparative reversed-phase HPLC column (MZ-PREPARATIVE; MZ-ANALYSENTECHNIK GmbH, Barcelona, Spain), 250 × 20 mm (silica gel C18, PerfectSil target ODS-3 5 µm) was used (flow rate 4.0 mL/min), with a system of 20% methanol in water—100% methanol.

### 3.3. Cell Assay

Leukemia cells from ATCC K562 (CCL-243) (Manassas, Virginia, USA) and HL60 (CCL-240) (Manassas, VA, USA) were cultured using RPMI 1640 medium (Servicebio, Wuhan Optie Bio City, Hubei, China) supplemented with 10% fetal bovine serum (FBS) and 2 mM L-glutamine. Cell cultures were incubated at 37 °C and 5% CO_2_. Neuroblastoma cells SH-SY5Y (CRL-2266) from ATCC were cultured in DMEM medium (Servicebio, Wuhan Optie Bio City, Hubei, China) containing 10% fetal bovine serum (FBS) at 37 °C and 5% CO_2_. Cells were seeded in 96-well plates at a density of 1 × 10^4^ cells/well. THLE-2 (CRL-2706) epithelial human liver cells from ATCC were cultured using THLE-2 Cell Complete Medium (Procellsystem, Singapore), and cells were incubated at 37 °C and 5% CO_2_. Test substances were then added in a concentration range from 0.8 to 100 μM. DMSO (1%) was used as a control, which corresponds to the percentage of DMSO when adding the highest concentration of the drug. The total well volume was 150 μL. The cells were incubated for 48 h at +37 °C and 5% CO_2_. Resazurin was added at 10 μL of a 0.5 mg/mL solution in phosphate-buffered saline. The cells were incubated under the same conditions for 4 h, and then the fluorescence level was measured at a wavelength of 590 nm using an excitation wavelength of 560 nm on a CLARIOstar multimodal microplate reader (BMG Labtech, Ortenberg, Germany). The data obtained for each concentration were normalized to the control, and the IC_50_ (half-maximal inhibitory concentration) was calculated using nonlinear regression. The minimum number of replicates for each concentration was three. To calculate IC_50_ and plot graphs of the percentage of living cells versus drug concentration, GraphPad Prism software v.9.5.1 (GraphPad Software, San Diego, CA, USA) was used.

### 3.4. Antituberculosis Tests

The virulent laboratory strain *M. tuberculosis* H37Rv was standardized by the number of colony-forming units (CFU) and growth phase, as described previously [9,33]. The anti-tuberculosis activity of the compounds was determined by microdilution in a 96-well plate. MTb culture was seeded at 10^5^ CFU per well and incubated with a series of two-fold compound dilutions (from 3.125 μg/mL to 100 μg/mL) for 14 days. The number of MTb cells per well was determined by quantitative real-time PCR (Amplitub-RV kit, Syntol, Moscow, Russia). The number of MTb cells was determined using a calibration curve relating the amplification threshold cycle values of the single-copy *regX* gene to the corresponding number of MTb cells. The anti-tuberculosis effect of the compounds was determined by comparing culture growth in wells with a series of compound dilutions and culture growth without the addition of the drugs. The anti-tuberculosis drug rifampicin at a critical concentration of 0.5 μg/mL was used as a positive control, and untreated cultures were used as a negative control. Experiments were performed in three independent replicates (*n* = 3). Statistical calculations were performed using GraphPad Prism 8.0 (GraphPad Software, San Diego, CA, USA).

### 3.5. Inhibition of ADA E. coli

Reaction mixtures contained 2.0 mM adenosine, 1 mM sodium phosphate buffer (pH 7.0), and 0.1 mM inhibitor: 6-amino-2-chloropurine 2′deoxyriboside (cladribine), 2′-deoxycoformycin (pentostatin), or compounds **1**–**16**. ADA (0.008 units) was added, and the reaction mixtures were incubated at 25 °C for 25 min. Conversion of adenosine into inosine was monitored each 5 min by HPLC. The production of adenine deaminase from *E. coli* and the determination of enzymatic activity were described previously. Experimental diagrams of the diasamenization rate are presented in the Supporting Information (Figures S66 and S67) (page 70).

### 3.6. Partial Charge Calculation

The partial charges of the atoms were calculated using the semi-empirical AM1-BCC method implemented in OpenBabel (version 3.1.1). The molecular structures were obtained by adding polar hydrogen atoms (pH 7.4) and geometry optimization (MMFF94) in RDKit (version 2024.03.1) [44].

### 3.7. Molecular Docking

Molecular docking was performed using AutoDock Vina (version 1.2.7, The Scripps Research Institute, San Diego, CA, USA). The receptor structure (PNP) was prepared from the PDB entry 7-deazahypoxanthine(7HX) using the co-crystallized ligand 5IU6 [45] (chain A) as a reference for the binding site definition. The grid box was centered at coordinates x = −19.683, y = 43.538, z = 22.013, with dimensions of 20 × 12 × 10 Å, covering the entire active site. The best-docked pose was selected based on the lowest binding energy (−4.88 kcal/mol) and visual inspection of the binding mode. The root-mean-square deviation (RMSD) value of 0.901 Å (≤2.0 Å) confirmed the correct ligand placement within the active site, validating the docking protocol. Molecular visualization and interaction analysis were performed using UCSF Chimera (version 1.16, Resource for Biocomputing, Visualization, and Informatics, San Francisco, CA, USA) [46].

## 4. Conclusions

Nucleoside derivatives of indazoles substituted at position 5 or 6 with bromine, pyrazole or pyrimidine fragments were synthesized for the first time using enzymatic transglycosylation using *E. coli* PNP. Despite significant structural differences between the synthesized bases and natural purines, all studied compounds, with the exception of derivative **6**, were effectively glycosylated by this enzyme. For three bases (**1**, **2** and **5**), the formation of N2-regioisomeric products was observed in the reaction mixtures, which complicated the isolation of the target N1-nucleosides. For indazole-pyrazole conjugates containing a second glycosylation center, the reaction resulted in the formation of the corresponding pyrazole nucleosides; however, bis-glycoside derivatives were not detected. The enzymatic transglycosylation of the studied heterocyclic bases proceeded with conversions of 65–100% (according to HPLC analysis). After chromatographic purification, the corresponding ribo- and 2′-deoxyribonucleosides were obtained in isolated yields of 19–94%. The yields were varying depending on the nature of the heterocyclic base and the ratio of regioisomeric products formed in the reaction.

A series of computational studies were conducted to elucidate the nature of the regioselectivity of enzymatic glycosylation and the reasons for the N2-regioisomer formation, as well as the exclusive reaction at the pyrazole center in compounds **3** and **4**. Calculation of partial atomic charges revealed that the nitrogen atom in the pyrazole fragment possesses a charge that favors the reaction in this direction. Molecular modeling methods allowed the identification of the preferred orientations of substrates in the active site of the enzyme, leading to the formation of N1- and N2-regioisomers of indazole. It also revealed that compound **6** participates in enhanced hydrophobic interactions, which likely prevents its enzymatic recognition. The obtained data allow us to conclude that the regioselectivity of glycosylation for the studied series of indazole derivatives is determined by several factors.

A comprehensive study of the biological activity of the synthesized compounds was performed. It includes an assessment of cytotoxicity, ADA inhibitory activity, and antimycobacterial properties. The synthesized nucleoside analogues did not show an inhibitory effect on the *E. coli* ADA or a cytotoxic effect on SH-SY5Y neuroblastoma cell lines, K-562 lymphoblastic cells, or HL-60 promyeloblastic cells. It was shown that indazole derivatives were able to inhibit mycobacterial growth. 2′-Deoxynucleoside **7** and ribonucleoside **12** inhibited the growth of 99.55% and 97.78% of *M. tuberculosis* culture at 100 μg/mL. The best anti-tuberculosis activity was shown for 5-(pyrimidin-5-yl)-1H-indazole **5** (99.40% inhibition of culture growth at a concentration of 100 μg/mL and 91.86% at a concentration of 50 μg/mL). These results indicate that the activity of parental indazole-pyrimidine heterocyclic base **5** was realized by mechanisms that do not imply intracellular conversion into the corresponding nucleosides. In contrast to conventional nucleoside antimetabolites, where the mechanism of action includes integration into nucleotide metabolism, the activity of indazole derivatives is likely attributed to the heterocyclic core itself. This is evidenced by the lower activity of nucleosides **11** and **12** compared to the starting base **5**, indicating that the sugar moiety either hinders target recognition or impedes cellular penetration. Obtained data confirm the potential of 5- and 6-substituted indazoles, both alone and as a part of corresponding nucleoside analogs, for further structural optimization in order to improve their functions.

## Figures and Tables

**Table 1:** Experimental data for the enzymatic synthesis of nucleosides ^a^.

Compound	Acceptor (mol wt)	Donor mol wt	Acceptor mg (mmol)	Donor mg (mmol)	Reaction Volume, mL	PNP ^b^, Units/ ml	UP ^b^, Units/ ml	Reaction Time, h	Conversion (HPLC Data), %	Yield, %
N1-(**7**) N2-(**7**)	1 (197.03)	dU ^c^	50 (0.254)	579 (2.537)	254	10.0	5.0	26	95	19 11
N1-(**8**) N2-(**8**)	1 (197.03)	Urd ^c^	50 (0.254)	620 (2.539)	254	10.0	5.0	192	96	42 12
N1-(**9**) N2-(**9**)	2 (197.03)	dU	50 (0.254)	579 (2.537)	254	10.0	5.0	12	100	42
N1-(**10**) N2-(**10**)	2 (197.03)	Urd	50 (0.254)	620 (2.539)	254	10.0	5.0	120	100	51 3
**11**	5 (196.20)	dU	20 (0.102)	93 (0.408)	102	3.2	4.0	336	74	41
**12**	5 (196.20)	Urd	50 (0.255)	622 (2.547)	255	10.0	5.0	480	65	47
**13**	3 (184.19)	dU	20 (0.109)	50 (0.219)	109	3.2	4.0	4	100	49
**14**	3 (184.19)	Urd	20 (0.109)	53.2 (0.218)	109	3.2	4.0	26	100	47
**15**	4 (184.19)	dU	20 (0.109)	50 (0.219)	109	3.2	4.0	8	100	93
**16**	4 (184.19)	Urd	20 (0.109)	53.2 (0.218)	109	3.2	4.0	26	100	94

^a^ Reactions were performed in 10 mM KH_2_PO_4_ (pH 7.0) at 50 °C under standard conditions. ^b^ Recombinant *E. coli* UP and PNP (1700 and 1400 units/mL, respectively) in 5 mM potassium phosphate buffer (pH 7.0) were used. ^c^ 2′-Deoxyuridine (dU), uridine (Urd).

**Table 2:** Molecular docking data.

Compound	Energy, kcal/mol	Interaction	Distance to Ser90, Å	Angle (Base/Phe159), °	Distance (Base/Phe159), Å	Experimental Confirmation of the Hypothesis
5-bromoindazole (**1**)	−5.69 −5.51	N1 N2	3.081 2.874	52.503 54.248	0.936 0.399	+ +
6- bromoindazole (**2**)	−5.49 −5.58	N1 N2	3.083 2.996	53.702 56.411	0.907 0.227	+ +
5-(1H-pyrazol-4-yl)-1H-indazole (**3**)	−7.27	N1 (indazole)	3.270	47.293	0.861	-
	−7.21	N1 (pyrazole)	3.245	61.588	0.083	+
6-(1H-pyrazol-4-yl)-1H-indazole (**4**)	−6.92	N1 (indazole)	3.051	48.945	1.201	-
	−7.04	N1 (pyrazole)	3.246	48.860	0.315	+
5-(pyrimidin-5-yl)-1H-indazole (**5**)	−7.15 −7.03	N1 N2	3.198 2.964	49.388 54.198	0.694 0.011	+ +
6-(pyrimidin-5-yl)-1H-indazole (**6**)	−6.40 −6.13	N1 N2	2.800 3.885	47.968 38.078	1.123 1.517	- -
7HX re-docking	−4.88	-	3.551	48.581	1.988	*
7HX experimental	-	-	4.253	50.522	1.641	*

* Inhibitor; + experimentally verified presence of the product in the reaction mixture.

**Table 3:** Antituberculosis activity of compounds.

	Compound		Number of MTb *, Cells/mL		
No.	Structure	Concentration, µg/mL	Mean	SD **	Inhibition, %
**1**	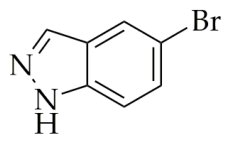	100 50 25 12.5 6.25 3.125	1.44 × 10^7^ 1.42 × 10^7^ 1.51 × 10^7^ 1.35 × 10^7^ 1.41 × 10^7^ 1.48 × 10^7^	1.79 × 10^6^ 1.91 × 10^6^ 8.98 × 10^5^ 9.43 × 10^5^ 1.26 × 10^6^ 1.40 × 10^6^	- - - - - -
**2**	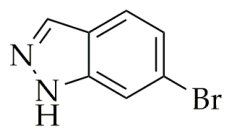	100 50 25 12.5 6.25 3.125	6.25 × 10^6^ 1.44 × 10^7^ 1.28 × 10^7^ 1.34 × 10^7^ 1.50 × 10^7^ 1.28 × 10^7^	2.10 × 10^6^ 1.00 × 10^6^ 7.02 × 10^5^ 1.46 × 10^6^ 6.71 × 10^5^ 1.40 × 10^6^	- - - - - -
**5**	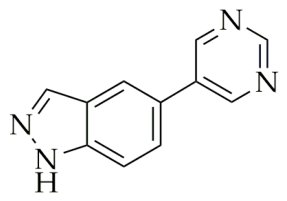	100 50 25 12.5 6.25 3.125	1.15 × 10^6^ 3.10 × 10^6^ 1.87 × 10^7^ 2.03 × 10^7^ 3.09 × 10^7^ 2.88 × 10^7^	7.29 × 10^4^ 2.10 × 10^5^ 1.40 × 10^6^ 1.39 × 10^6^ 7.09 × 10^5^ 2.16 × 10^6^	99.40 91.86 31.42 25.03 - -
**7**	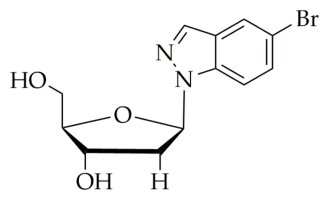	100 50 25 12.5 6.25 3.125	1.12 × 10^6^ 1.34 × 10^7^ 2.24 × 10^7^ 2.42 × 10^7^ 2.49 × 10^7^ 3.13 × 10^7^	7.73 × 10^4^ 1.04 × 10^6^ 1.46 × 10^6^ 1.66 × 10^6^ 5.71 × 10^5^ 2.35 × 10^6^	99.55 52.03 - - - -
**8**	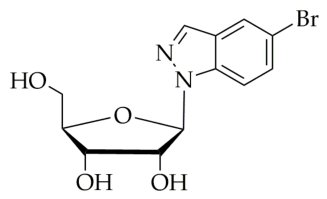	100 50 25 12.5 6.25 3.125	1.24 × 10^7^ 2.01 × 10^7^ 2.17 × 10^7^ 2.41 × 10^7^ 2.26 × 10^7^ 3.17 × 10^7^	1.45 × 10^6^ 6.42 × 10^5^ 1.71 × 10^6^ 2.62 × 10^6^ 1.64 × 10^6^ 3.29 × 10^6^	55.60 25.81 19.86 - - -
**10**	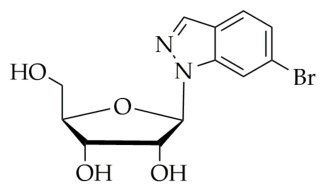	100 50 25 12.5 6.25 3.125	8.75 × 10^6^ 1.89 × 10^7^ 2.31 × 10^7^ 2.58 × 10^7^ 2.19 × 10^7^ 2.90 × 10^7^	2.05 × 10^5^ 1.18 × 10^6^ 2.11 × 10^6^ 2.85 × 10^6^ 2.36 × 10^6^ 2.80 × 10^6^	69.94 30.51 - - - -
**11**	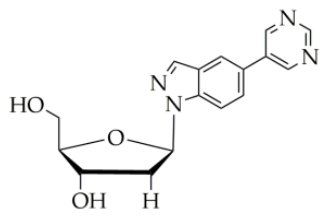	100 50 25 12.5 6.25 3.125	4.56 × 10^6^ 1.77 × 10^7^ 2.39 × 10^7^ 2.35 × 10^7^ 2.95 × 10^7^ 2.45 × 10^7^	4.37 × 10^5^ 9.52 × 10^5^ 1.11 × 10^6^ 1.58 × 10^6^ 2.21 × 10^6^ 2.46 × 10^6^	86.19 35.32 - - - -
**12**	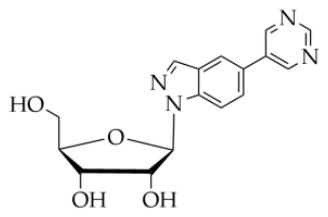	100 50 25 12.5 6.25 3.125	1.57 × 10^6^ 2.17 × 10^7^ 2.35 × 10^7^ 2.72 × 10^7^ 2.54 × 10^7^ 3.01 × 10^7^	1.03 × 10^5^ 2.33 × 10^6^ 2.24 × 10^6^ 2.21 × 10^6^ 2.45 × 10^6^ 2.78 × 10^6^	97.78 19.88 - - - -
Rifampicin		0.5	8.24 × 10^5^	7.06 × 10^4^	100
Control		-	2.68 × 10^7^	5.12 × 10^6^	-

* The estimated number of MTb cells determined by quantitative PCR. ** Standard deviation (SD). The experiments were performed 3 times.

## Data Availability

The data presented in this study are available within the article.

## Supplementary Materials

The following supporting information can be downloaded at: https://www.mdpi.com/article/10.3390/ijms27177551/s1.

## References

[1] Rodríguez-Villar K., Hernández-Campos A., Yépez-Mulia L., Sainz-Espuñes T.d.R., Soria-Arteche O., Palacios-Espinosa J.F., Cortés-Benítez F., Leyte-Lugo M., Varela-Petrissans B., Quintana-Salazar E.A. (2021). Design, Synthesis and Anticandidal Evaluation of Indazole and Pyrazole Derivatives. Pharmaceuticals.

[2] Singampalli A., Kumar P., Bandela R., Bellapukonda S.M., Nanduri S., Yaddanapudi V.M. (2025). Indazole—An Emerging Privileged Scaffold: Synthesis and Its Biological Significance. RSC Med. Chem..

[3] Boddapati S.N.M., Chalapaka B., Kola A.E., Jonnalagadda S.B. (2025). Recent Advances in Synthetic Strategies and Biological Properties of Indazole Scaffolds: A Review. Top. Curr. Chem..

[4] Zivi A., Cerbone L., Recine F., Sternberg C.N. (2012). Safety and Tolerability of Pazopanib in the Treatment of Renal Cell Carcinoma. Expert Opin. Drug Saf..

[5] Scott L.J. (2017). Niraparib: First Global Approval. Drugs.

[6] Moore P.K., Babbedge R.C., Wallace P., Gaffen Z.A., Hart S.L. (1993). 7-Nitro Indazole, an Inhibitor of Nitric Oxide Synthase, Exhibits Anti-Nociceptive Activity in the Mouse without Increasing Blood Pressure. Br. J. Pharmacol..

[7] Claramunt R.M., López C., Pérez-Medina C., Pérez-Torralba M., Elguero J., Escames G., Acuña-Castroviejo D. (2009). Fluorinated Indazoles as Novel Selective Inhibitors of Nitric Oxide Synthase (NOS): Synthesis and Biological Evaluation. Bioorg. Med. Chem..

[8] Nanda S.S., Yi D.K., Panda O.P., Chigurupati S., Mohapatra T.K., Hossain M.I. (2022). Impact of Indazole Scaffold as Antibacterial and Antifungal Agent. Curr. Top. Med. Chem..

[9] Khandazhinskaya A., Kondrashova E., Sokhraneva V., Novikova O., Velikorodnaya Y., Gorshenin A., Andreevskaya S., Smirnova T., Moroz M., Kirillov I. (2025). New Indazole Derivatives as Potential Scaffolds for the Development of Anticancer, Antiviral, and Anti-Tuberculosis Chemotherapeutic Compounds. Curr. Med. Chem..

[10] Burke A., Di Filippo M., Spiccio S., Schito A.M., Caviglia D., Brullo C., Baumann M. (2023). Antimicrobial Evaluation of New Pyrazoles, Indazoles and Pyrazolines Prepared in Continuous Flow Mode. Int. J. Mol. Sci..

[11] Karrouchi K., Radi S., Ramli Y., Taoufik J., Mabkhot Y.N., Al-aizari F.A., Ansar M. (2018). Synthesis and Pharmacological Activities of Pyrazole Derivatives: A Review. Molecules.

[12] Rocha S., Silva J., Silva V.L.M., Silva A.M.S., Corvo M.L., Freitas M., Fernandes E. (2024). Pyrazoles Have a Multifaceted Anti-Inflammatory Effect Targeting Prostaglandin E2, Cyclooxygenases and Leukocytes’ Oxidative Burst. Int. J. Biochem. Cell Biol..

[13] Reddy T.S., Kulhari H., Reddy V.G., Bansal V., Kamal A., Shukla R. (2015). Design, Synthesis and Biological Evaluation of 1,3-Diphenyl-1H-Pyrazole Derivatives Containing Benzimidazole Skeleton as Potential Anticancer and Apoptosis Inducing Agents. Eur. J. Med. Chem..

[14] Alam A.M. (2022). Antibacterial Pyrazoles: Tackling Resistant Bacteria. Future Med. Chem..

[15] Bennani F.E., Doudach L., Cherrah Y., Ramli Y., Karrouchi K., Ansar M., Faouzi M.E.A. (2020). Overview of Recent Developments of Pyrazole Derivatives as an Anticancer Agent in Different Cell Line. Bioorg. Chem..

[16] Sacha T., Saglio G. (2018). Nilotinib in the Treatment of Chronic Myeloid Leukemia. Future Oncol..

[17] Vuorinen K., Gao F., Oury T.D., Kinnula V.L., Myllärniemi M. (2007). Imatinib mesylate inhibits fibrogenesis in asbestos-induced interstitial pneumonia. Exp. Lung Res..

[18] Zhai X., Li W., Li J., Jia W., Jing W., Tian Y., Xu S., Li Y., Zhu H., Yu J. (2021). Therapeutic Effect of Osimertinib plus Cranial Radiotherapy Compared to Osimertinib Alone in NSCLC Patients with EGFR-Activating Mutations and Brain Metastases: A Retrospective Study. Radiat. Oncol..

[19] Bowman C., Dolton M., Ma F., Cheeti S., Kuruvilla D., Sane R., Kassir N., Chen Y. (2024). Understanding CYP3A4 and P-gp Mediated Drug–Drug Interactions through PBPK Modeling–Case Example of Pralsetinib. CPT Pharmacomet. Syst. Pharmacol..

[20] Gupta S., Vingerhoets J., Fransen S., Tambuyzer L., Azijn H., Frantzell A., Paredes R., Coakley E., Nijs S., Clotet B. (2011). Connection Domain Mutations in HIV-1 Reverse Transcriptase Do Not Impact Etravirine Susceptibility and Virologic Responses to Etravirine-Containing Regimens. Antimicrob. Agents Chemother..

[21] Masikini P., Mpondo B.C.T. (2015). HIV Drug Resistance Mutations Following Poor Adherence in HIV-Infected Patient: A Case Report. Clin. Case Rep..

[22] Wong C.K., Harding G.K., Ronald A.R., Hoban S. (1975). Trimethoprim-Resistant Enterobacteriaceae in Urinary Tract Infection. Can. Med. Assoc. J..

[23] Krievins D., Brandt R., Hawser S., Hadvary P., Islam K. (2009). Multicenter, Randomized Study of the Efficacy and Safety of Intravenous Iclaprim in Complicated Skin and Skin Structure Infections. Antimicrob. Agents Chemother..

[24] Dhindsa R.S., Zoghbi A.W., Krizay D.K., Vasavda C., Goldstein D.B. (2021). A Transcriptome-Based Drug Discovery Paradigm for Neurodevelopmental Disorders. Ann. Neurol..

[25] Perrone D., Marchesi E., Preti L., Navacchia M.L. (2021). Modified Nucleosides, Nucleotides and Nucleic Acids via Click Azide-Alkyne Cycloaddition for Pharmacological Applications. Molecules.

[26] Kazimierczuk Z., Lönnberg H., Vilpo J., Pfleiderer W. (1989). Nucleosides, XLIV1 Synthesis, Properties and Biological Activity of Indazole Nucleosides. Nucleosides Nucleotides Nucleotides Acids.

[27] Seela F., Bourgeois W. (1991). Stereoselective Synthesis of Indazole 2′-Deoxy-β-D-ribonucleosides: Glycosylation of the Nucleobase Anion. Helv. Chim. Acta.

[28] Seela F., Peng X. (2004). Regioselective Synthesis of Indazole N1- and N2-(Beta-D-Ribonucleosides). Nucleosides Nucleotides Nucleotides Acids.

[29] Preobrazhenskaya M.N., Korbukh I.A. (1995). ChemInform Abstract: The Synthesis and Reactions of Pyrrole, Pyrazole, Triazole, Indole, Indazole, and Benzotriazole Nucleosides and Nucleotides. ChemInform.

[30] Vichier-Guerre S., Ku T.C., Pochet S., Seley-Radtke K.L. (2020). An Expedient Synthesis of Flexible Nucleosides through Enzymatic Glycosylation of Proximal and Distal Fleximer Bases. Chembiochem.

[31] Khandazhinskaya A., Eletskaya B., Fateev I., Kharitonova M., Konstantinova I., Barai V., Azhayev A., Hyvonen M.T., Keinanen T.A., Kochetkov S. (2021). Novel Fleximer Pyrazole-Containing Adenosine Analogues: Chemical, Enzymatic and Highly Efficient Biotechnological Synthesis. Org. Biomol. Chem..

[32] Esipov R.S., Gurevich A.I., Chuvikovsky D.V., Chupova L.A., Muravyova T.I., Miroshnikov A.I. (2002). Overexpression of Escherichia Coli Genes Encoding Nucleoside Phosphorylases in the pET/Bl21(DE3) System Yields Active Recombinant Enzymes. Protein Expr. Purif..

[33] Khandazhinskaya A., Eletskaya B., Mironov A., Konstantinova I., Efremenkova O., Andreevskaya S., Smirnova T., Chernousova L., Kondrashova E., Chizhov A. (2023). New Flexible Analogues of 8-Aza-7-Deazapurine Nucleosides as Potential Antibacterial Agents. Int. J. Mol. Sci..

[34] Eletskaya B.Z., Mironov A.F., Fateev I.V., Berzina M.Y., Antonov K.V., Smirnova O.S., Zatsepina A.B., Arnautova A.O., Abramchik Y.A., Paramonov A.S. (2024). Enzymatic Transglycosylation Features in Synthesis of 8-Aza-7-Deazapurine Fleximer Nucleosides by Recombinant *E. Coli* PNP: Synthesis and Structure Determination of Minor Products. Biomolecules.

[35] Vichier-Guerre S., Dugué L., Bonhomme F., Pochet S. (2016). Expedient and Generic Synthesis of Imidazole Nucleosides by Enzymatic Transglycosylation. Org. Biomol. Chem..

[36] Stachelska-Wierzchowska A., Wierzchowski J., Górka M., Bzowska A., Stolarski R., Wielgus-Kutrowska B. (2020). Tricyclic Nucleobase Analogs and Their Ribosides as Substrates and Inhibitors of Purine-Nucleoside Phosphorylases III. Aminopurine Derivatives. Molecules.

[37] Stachelska-Wierzchowska A., Wierzchowski J. (2024). Chemo-Enzymatic Generation of Highly Fluorescent Nucleoside Analogs Using Purine-Nucleoside Phosphorylase. Biomolecules.

[38] Büchel G.E., Stepanenko I.N., Hejl M., Jakupec M.A., Keppler B.K., Heffeter P., Berger W., Arion V.B. (2012). Osmium(IV) Complexes with 1H- and 2H-Indazoles: Tautomer Identity versus Spectroscopic Properties and Antiproliferative Activity. J. Inorg. Biochem..

[39] Arnautova A.O., Aleksakhina I.A., Zorina E.A., Berzina M.Y., Fateev I.V., Eletskaya B.Z., Antonov K.V., Smirnova O.S., Paramonov A.S., Kayushin A.L. (2025). New Benzimidazole 3’-Deoxynucleosides: Synthesis and Antiherpes Virus Properties. Biomolecules.

[40] Fateev I.V., Kharitonova M.I., Antonov K.V., Konstantinova I.D., Stepanenko V.N., Esipov R.S., Seela F., Temburnikar K.W., Seley-Radtke K.L., Stepchenko V.A. (2015). Recognition of Artificial Nucleobases by *E. Coli* Purine Nucleoside Phosphorylase versus Its Ser90Ala Mutant in the Synthesis of Base-Modified Nucleosides. Chemistry.

[41] Verma S.K., Dubey A.L., Singh P.A., Tewerson S.L., Sharma D. (2008). Adenosine Deaminase (ADA) Level in Tubercular Pleural Effusion. Lung India.

[42] Kutryb-Zajac B., Mierzejewska P., Slominska E.M., Smolenski R.T. (2020). Therapeutic Perspectives of Adenosine Deaminase Inhibition in Cardiovascular Diseases. Molecules.

[43] Eletskaya B.Z., Gruzdev D.A., Krasnov V.P., Levit G.L., Kostromina M.A., Paramonov A.S., Kayushin A.L., Muzyka I.S., Muravyova T.I., Esipov R.S. (2019). Enzymatic Synthesis of Novel Purine Nucleosides Bearing a Chiral Benzoxazine Fragment. Chem. Biol. Drug Des..

[44] Jakalian A., Bush B.L., Jack D.B., Bayly C.I. (2000). Fast, Efficient Generation of High-Quality Atomic Charges. AM1-BCC Model: I. Method. J. Comput. Chem..

[45] Timofeev V.I., Zhukhlistova N.E., Abramchik Y.A., Fateev I.I., Kostromina M.A., Muravieva T.I., Esipov R.S., Kuranova I.P. (2018). Crystal Structure of *Escherichia Coli* Purine Nucleoside Phosphorylase in Complex with 7-Deazahypoxanthine. Acta Crystallogr. Sect. F. Struct. Biol. Commun..

[46] Pettersen E.F., Goddard T.D., Huang C.C., Couch G.S., Greenblatt D.M., Meng E.C., Ferrin T.E. (2004). UCSF Chimera—A Visualization System for Exploratory Research and Analysis. J. Comput. Chem..

