# Supplementary material for: Exploring Novel Indazole and Pyrazole Nucleoside Analogues: Enzymatic Synthesis, Characterization, and Biological Evaluation
Source: Int J Mol Sci. 2026 Aug 24;27(17):7551. doi: 10.3390/ijms27177551 (PMC13566874; doi:10.3390/ijms27177551)
Supplement: Supplementary file 1 [file ijms-27-07551-s001.zip › ijms-4486535-supplementary.pdf]

Supporting Information

Exploring Novel Indazole and Pyrazole Nucleoside Analogues: Enzymatic Synthesis, Characterization, and Biological Evaluation

Anton F. Mironov [1,2], Barbara Z. Eletskaya[1], Konstantin V. Antonov[1], Alexander S. Paramonov[1], Roman S. Esipov[1], Irina D. Konstantinova[1], Sofya N. Andreevskaya[3], Tatiana G. Smirnova[3], Vera A. Sokhraneva[4], Iulia S. Zhivotova[4], Inna L. Karpenko[4], Sergei N. Kochetkov[4], Anastasia L. Khandazhinskaya[4] and Elena S. Matyugina[4]*

[1] Shemyakin-Ovchinnikov Institute of Bioorganic Chemistry, Russian Academy of Sciences, Mklukho-Maklaya St. 16/10, 117997 Moscow, Russia; anton-mironov1999@inbox.ru (A.F.M.); fraubarusya@gmail.com (B.Z.E.); antonov.kant@yandex.ru (K.V.A.); a.s.paramonov@gmail.com (A.S.P.); esipov@ibch.ru (R.S.E.); kid1968@yandex.ru (I.D.K.)

[2] Institute of Pharmacy and Biotechnology, Peoples' Friendship University of Russia Named after Patrice Lumumba, Miklukho-Maklaya St. 6, 117198 Moscow, Russia

[3] Central Tuberculosis Research Institute, 2 Yauzskaya Alley, 107564 Moscow, Russia; andsofia@mail.ru (S.N.A.); s_tatka@mail.ru (T.G.S.)

[4] Engelhardt Institute of Molecular Biology, Russian Academy of Sciences, Vavilov St. 32, 119991 Moscow, Russia; ilkzkil@gmail.com (I.L.K.); sokhraneva.v@mail.ru (V.A.S.); zhivotovajulia51@gmail.com (I.S.Z.); snk1952@gmail.com (S.N.K.); khandazhinskaya@bk.ru (A.L.K.)

* Correspondence: matyugina@gmail.com

Table of Content

Content	Page number
List of abbreviations	2
Table of physicochemical properties of nucleoside **7-16**.	3
NMR spectra, HMRS and HPLC data of compounds **7-16**.	5-56
HPLC data of reaction mixtures	57-69
ADA *E. coli* inhibition data	70

List of abbreviations

Abbreviation	Full form
**ADA**	Adenosine deaminase
**AM1-BCC**	Austin Model 1 – Bond Charge Correction
**CETSA**	Cellular thermal shift assay
**CFU**	Colony-forming unit
**CRediT**	Contributor Roles Taxonomy
**DNA**	Deoxyribonucleic acid
**DMSO**	Dimethyl sulfoxide
**DMF**	Dimethylformamide
**DMEM**	Dulbecco's Modified Eagle Medium
**dU**	2′-Deoxyuridine
**ESI-MS**	Electrospray ionization mass spectrometry
**FBS**	Fetal bovine serum
**IC_{50}**	Half-maximal inhibitory concentration
**HPLC**	High-performance liquid chromatography
**HRMS**	High-resolution mass spectrometry
**LNDT**	*L. leichmannii* nucleoside deoxyribosyltransferase
**LC-MS**	Liquid chromatography–mass spectrometry
**MTb**	*Mycobacterium tuberculosis*
**NOS**	Nitric oxide synthase
**NMR**	Nuclear magnetic resonance
**PNP**	Purine nucleoside phosphorylase
**RMSD**	Root-mean-square deviation
**RPMI**	Roswell Park Memorial Institute medium
**SD**	Standard deviation
**TFA**	Trifluoroacetic acid
**Urd**	Uridine
**UP**	Uridine phosphorylase
**VEGFR**	Vascular endothelial growth factor receptor

Table S1. Physicochemical properties of nucleoside **7-16**.

**Compound**	**Yield (%), (mg)**	**N1 and N2 isomer content (%)**	**Purity (%)**	**RT, min**	**UV λmax, nm (ε)**	**HRMS: m/z calcd [M+H]⁺**	**HRMS: m/z found [M+H]⁺**
(N1)-**7**	19<br>(15.4)	N1 – 64	99.25 (N1)	10.383<br>(N1)	304 (2582),<br>279 (3083),<br>232 (1894)	196.9709<br>(base)<br>198.9694<br>(base)<br>313.0182<br>315.0167	196.9720<br>315.0154
N2)-**7**	11<br>(8.7)	N2 – 36	99.66 (N2)	10.565<br>(N2)	299 (n/d)<br>253 (n/d)<br>228 (n/d)		196.9709<br>313.0159
(N1)-**8**	42<br>(34.8)	N1 – 77	98.34 (N1)	9.424<br>(N1)	299 (2704),<br>254 (3394),<br>229 (5519)	196.9709<br>(base)<br>198.9694<br>(base)<br>329.0131<br>331.0112	196.9718<br>331.0092
(N2)-**8**	12<br>(10.4)	N2 – 23	99.65 (N2)	9.601<br>(N2)	300 (n/d),<br>269 (n/d),<br>224 (n/d)		196.9719<br>331.0094
(N1)-**9**<br>+<br>(N2)-**9**	42<br>(33.3)	N1 – 79<br>N2 – 21	n/d	10.395<br>(N1+N2)	290 (n/d),<br>276 (n/d),<br>265 (n/d),<br>228 (n/d)	196.9709<br>(base)<br>198.9694<br>(base)<br>313.0188<br>315.0167	196.9712<br>315.0141
(N1)-**10**	51<br>(42.2)	N1 – 94	99.12 (N1)	9.202<br>(N1)	291 (3888),<br>260 (5964),<br>228 (2998)	196.9709<br>(base)<br>198.9694<br>(base)<br>329.0131<br>331.0112	196.9716<br>331.0090
(N2)-**10**	3<br>(2.7)	N2 – 6	99.34 (N2)	9.456<br>(N2)	301 (n/d),<br>285 (n/d),<br>230 (n/d).		196.9716<br>329.0110<br>331.0091
**11**	41<br>(12.5)	N1 – 97<br>N2 – 3	98.26 (N1)<br>n/d (N2)	7.201<br>(N1)	279 (9096),<br>244<br>(27955),<br>197<br>(19175)	197.0827<br>(base)<br>313.1295	197.0822<br>313.1261
**12**	47<br>(39.1)	N1 – 94<br>N2 – 6	99.02 (N1)<br>n/d (N2)	6.597<br>(N1)	273 (9360),<br>242<br>(26962),<br>225<br>(13186)	197.0827<br>(base)<br>329.1244	197.0801<br>329.1236
**13**	49<br>(12.0)	N1 –100	98.01	7.003	299 (2712),<br>237<br>(27242),<br>211<br>(21413)	185.0822<br>(base)<br>301.1295	185.0798<br>301.1290
**14**	47<br>(16.1)	N1 –100	99.47	6.166	299 (3057),<br>236<br>(28176),<br>209<br>(23790)	185.0822<br>(base)<br>317.1244	185.0844<br>317.1241
**15**	93<br>(30.4)	N1 –100	98.85	7.583	282<br>(13083),<br>234<br>(27356),<br>200<br>(26109)	185.0822<br>(base)<br>301.1295	185.0830<br>301.1269

**16**	94 (32.4)	N1 –100	98.38	7.007	282 (12751), 234 (27319), 200 (23718)	185.0822 (base) 317.1244	317.1242

NMR spectra, HMRS and HPLC data of compounds 7-16

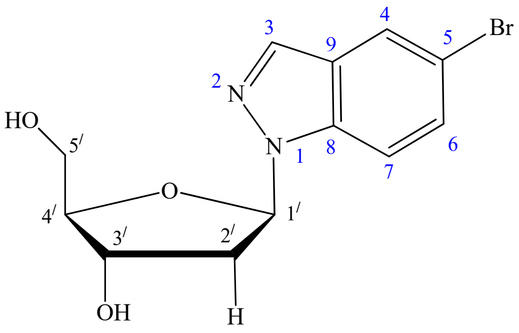

(N1)-**7**

N1-(β-D-2'deoxyribofuranosyl)-5-bromo-indazole ((N1)-7)

^{1}H NMR: δ=8.14, (s, 1H-weak, H-3 indazole),8.02 (br.d., J = 1.75 Hz, 1H-weak, H-4 indazole),7.80 (d., J = 8.97 Hz, 1H-weak , H-7 indazole), 7.53 (br.dd, J = 1.98 Hz; 10.86 Hz, 1H-weak , H-6 indazole), 6.66 (t, J = 5.08 Hz; 11.85 Hz, 1H, H-1′), 5.27 (d, J = 4.57 Hz 1H, OH-3′), 4.69 (t, J = 5.59 Hz; 11.34 Hz, 1H, OH-5′), 4.45 (m, 1H, H-3′), 3.84 (m, 1H, H-4′), 3.48 (m, 1H, H_a-5′), 3.32 (m, 1H, H_b-5′, overlapped by the solvent signal), 2.90 (m, 1H, H_a-2′), 2.30 (m, 1H, H_b-2′). ^{13}C NMR: δ= 138.19 (C8 indazole), 133.30 (C3 indazole), 128.93 (C6 indazole), 125.51 (C9 indazole), 123.01 (C4 indazole), 113.10 (C5 indazole), 111.87 (C7 indazole), 87.31 (C4′), 85.55 (C1′), 70.71 (C3′), 62.04 (C5′), 38.02 (C2′). ^{15}N NMR: δ = 316.65 (N2), 199.48 (N1).

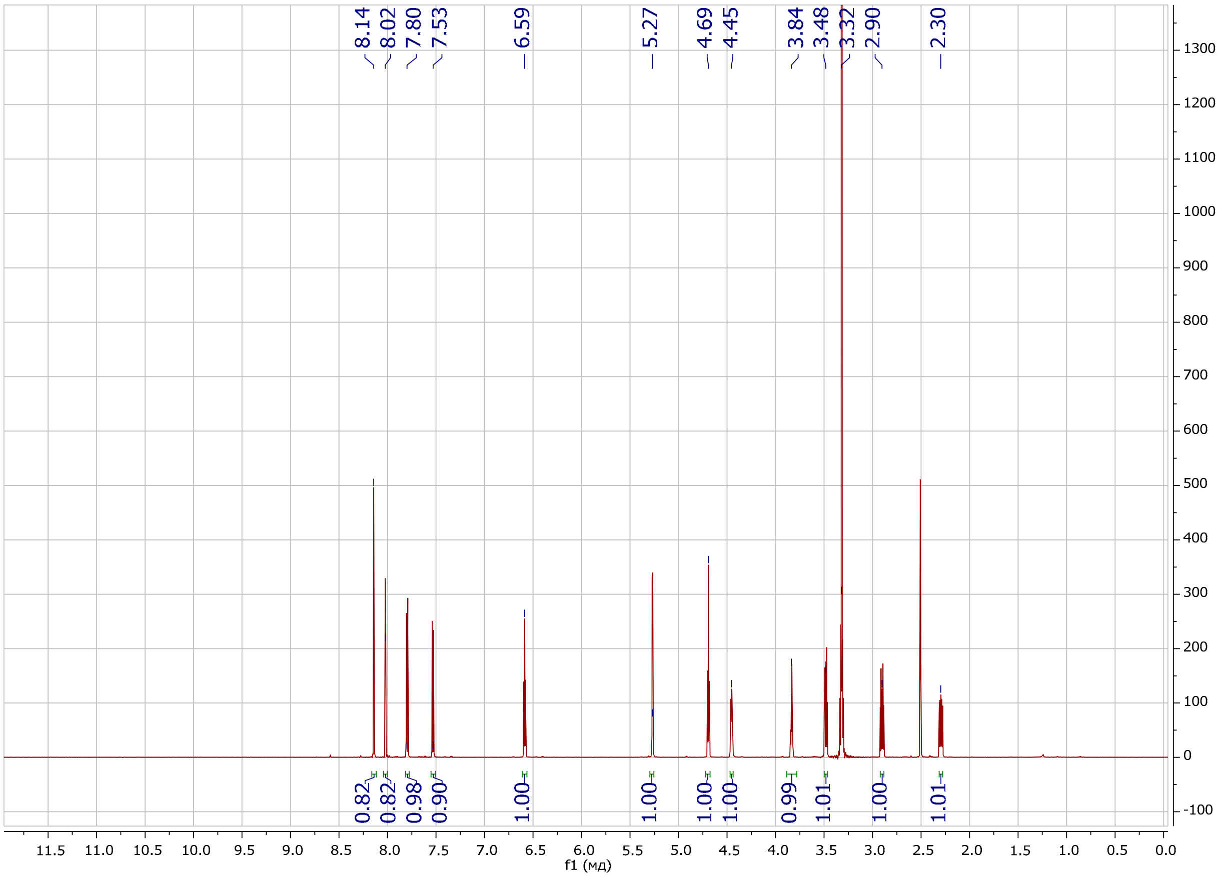

Figure S1. The ^{1}H NMR spectrum of N1-(β-D-2'deoxyribofuranosyl)-5-bromo-indazole (N1)-**7** (700 MHz, DMSO-d_6, 303 K).

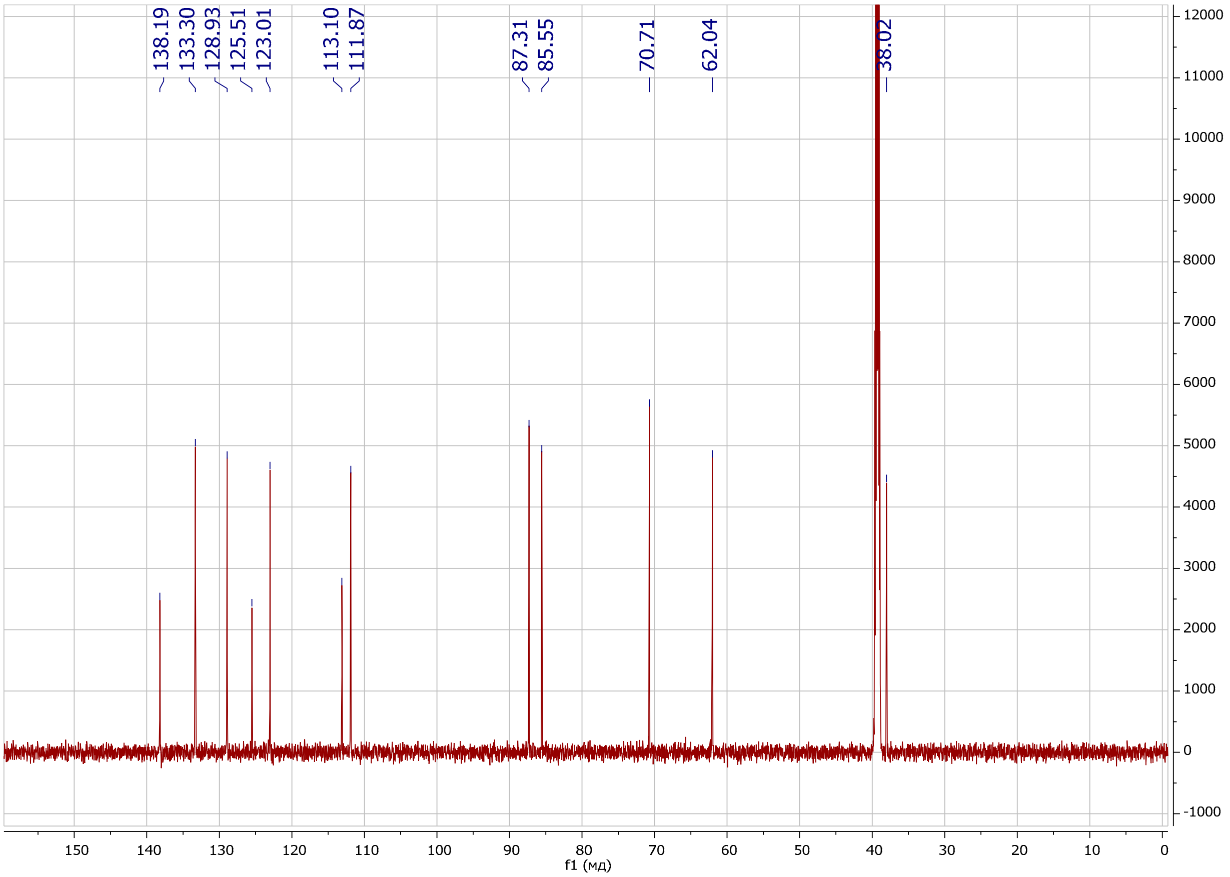

Figure S2. The ^{13}C NMR spectrum of N1-(β-D-2'deoxyribofuranosyl)-5-bromo-indazole (N1)-**7** (176 MHz, DMSO-d_6, 303 K).

Display Report

Analysis Info

Analysis Name	D:\Data\Ikar\2026 april\strangeweek\5-Br-Indz-2etdRib N1(8) 240426_32_01_6577.d	Acquisition Date	4/24/2026 3:02:02 PM
Method	ik-deltasmall-1600-positive .m	Operator	BDAL@DE
Sample Name	5-Br-Indz-2etdRib N1(8) 240426	Instrument	compact 8255754.20088
Comment			

Acquisition Parameter

Source Type	ESI	Ion Polarity	Positive	Set Nebulizer	0.4 Bar
Focus	Active	Set Capillary	4500 V	Set Dry Heater	180 °C
Scan Begin	50 m/z	Set End Plate Offset	-500 V	Set Dry Gas	4.0 l/min
Scan End	1600 m/z	Set Charging Voltage	2000 V	Set Divert Valve	Source
		Set Corona	0 nA	Set APCI Heater	0 °C

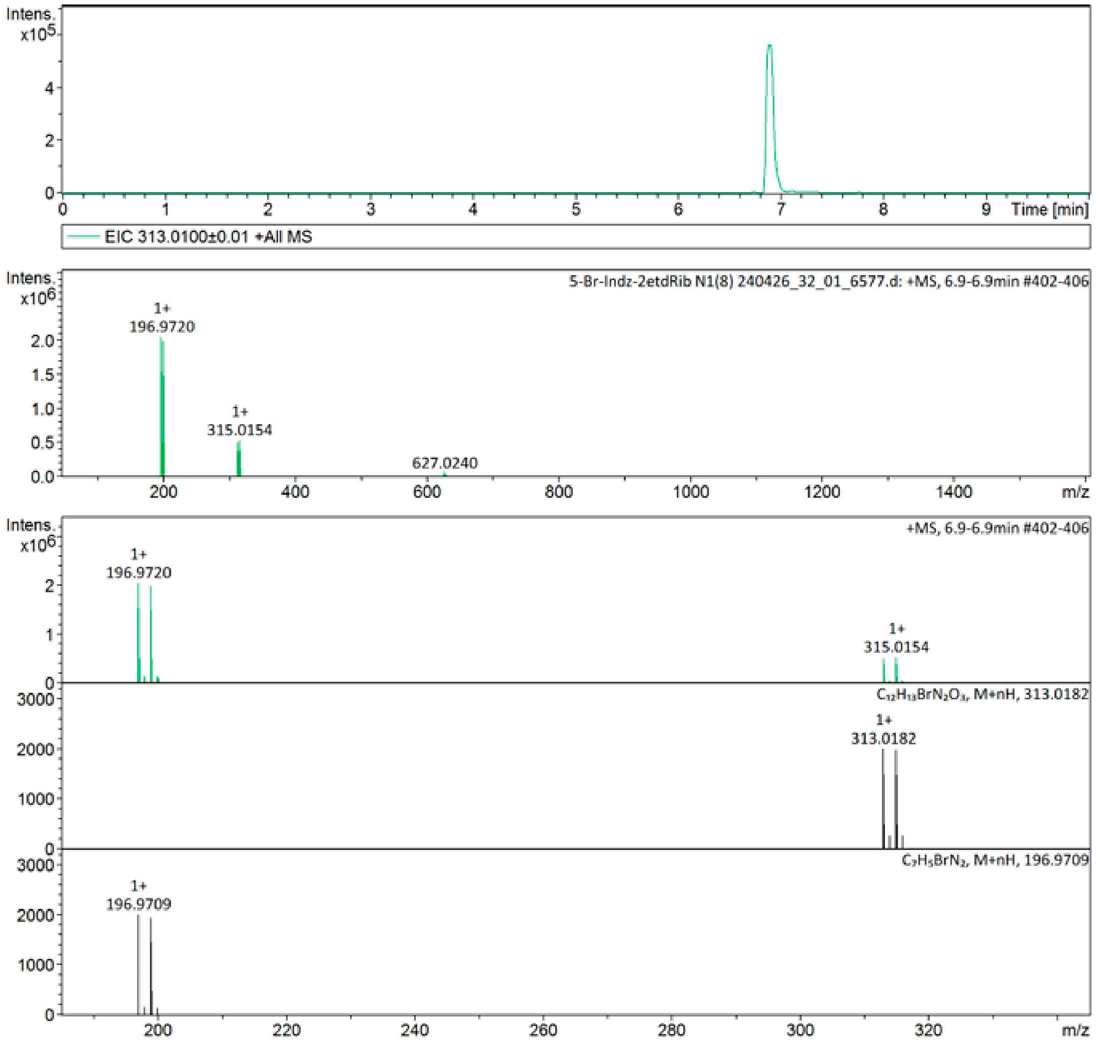

5-Br-Indz-2etdRib N1(8) 240426_32_01_6577.d

Bruker Compass DataAnalysis 4.3 printed: 4/24/2026 3:36:42 PM by: BDAL@DE Page 1 of 1

Figure S3. The HRMS data of (**N**1)-**7.**

IBCH RAS
Project Name Defaults
Reported by User: Breeze user (Breeze)

Breeze 2 HPLC System

SAMPLE INFORMATION

Sample Name:	5-br-indz-2'dr purity pê 1	Acquired By:	Breeze
Sample Type:	Unknown	Date Acquired:	10.11.2025 13:59:59 GMT-4
Vial:	1	Acq. Method:	100B_dual_280nm_05ml
Injection #:	1	Date Processed:	10.11.2025 14:23:01 GMT-4
Injection Volume:	10.00 ul	Channel Name:	W2489 ChB
Run Time:	20.00 Minutes	Channel Desc.:	W2489 ChB 280nm
Column Type:		Sample Set Name	ê

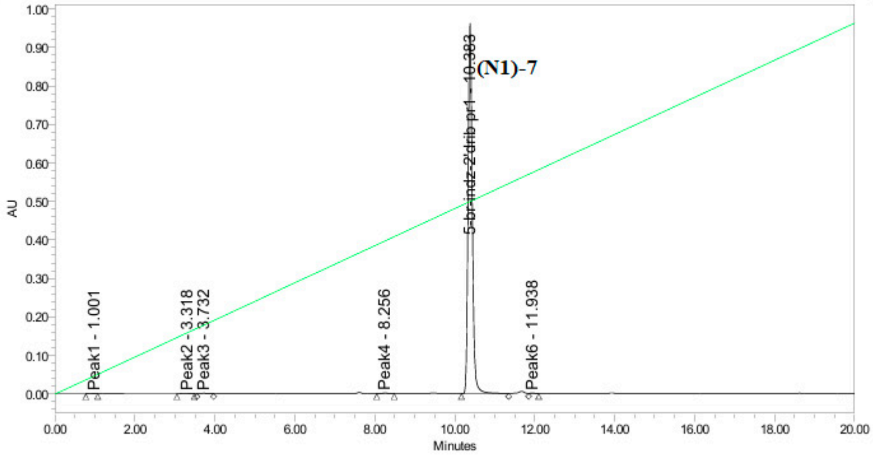

	Peak Name	RT (min)	Area (µV*sec)	% Area	Height (µV)	% Height
1	Peak1	1.001	5183	0.07	551	0.06
2	Peak2	3.318	12726	0.18	1063	0.11
3	Peak3	3.732	14804	0.21	1298	0.13
4	Peak4	8.256	11970	0.17	1688	0.18
5	5-br-indz-2'drib pr1	10.383	7032766	99.25	958281	99.39
6	Peak6	11.938	8652	0.12	1258	0.13

Report Method: Gradient Overlay Report ASC
Page: 1 of 1

Printed: 10.11.2025
14:23:26 Etc/GMT-4

Figure S4. The HPLC data of (N1)-**7.**

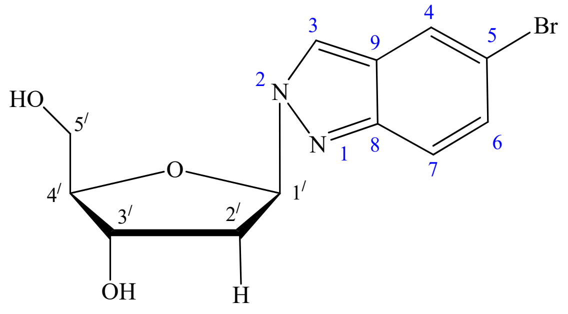

(N2)-7

N2-(β-D-2'deoxyribofuranosyl)-5-bromo-indazole ((N2)-7).

^{1}H NMR: δ=8.59, (s, 1H, H-3 indazole), 7.98 (br.d., J = 1.65 Hz, 1H-weak, H-4 indazole),7.61 (d., J = 9.30 Hz, 1H, H-7 indazole), 7.34 (br.dd, J = 1.70 Hz; 11.18 Hz, 1H, H-6 indazole), 6.40 (t, J = 6.27 Hz; 12.39 Hz, 1H, H-1′), 5.31 (s, 1H, OH-3′), 4.92 (s, 1H, OH-5′), 4.43 (m, 1H, H-3′), 3.93 (m, 1H, H-4′), 3.61 (m, 1H, H_a-5′), 3.50 (m, 1H, H_b-5′), 2.66 (m, 1H, H_a-2′), 2.40 (m, 1H, H_b-2′).

^{13}C NMR: δ= 146.54 (C8 indazole), 129.40 (C6 indazole), 123.35 (C4 indazole), 123.01 (C3 indazole), 122.53 (C9 indazole), 119.89 (C7 indazole), 114.08 (C5 indazole), 90.87 (C1′), 88.60 (C4′), 70.72 (C3′), 62.04 (C5′), 40.85 (C2′).

^{15}N NMR: δ = 243.46 (N2).

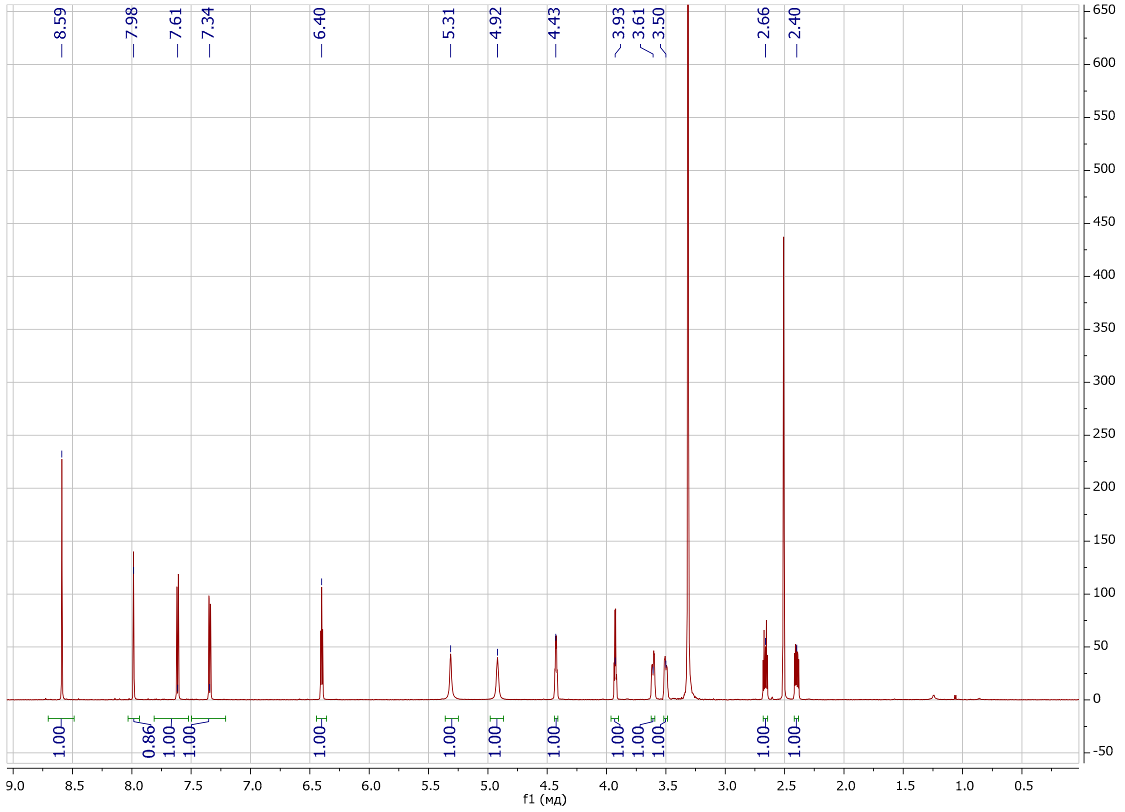

Figure S5. The ^{1}H NMR spectrum of N2-(β-D-2'deoxyribofuranosyl)-5-bromo-indazole (N2)-**7** (700 MHz, DMSO-d_6, 303 K).

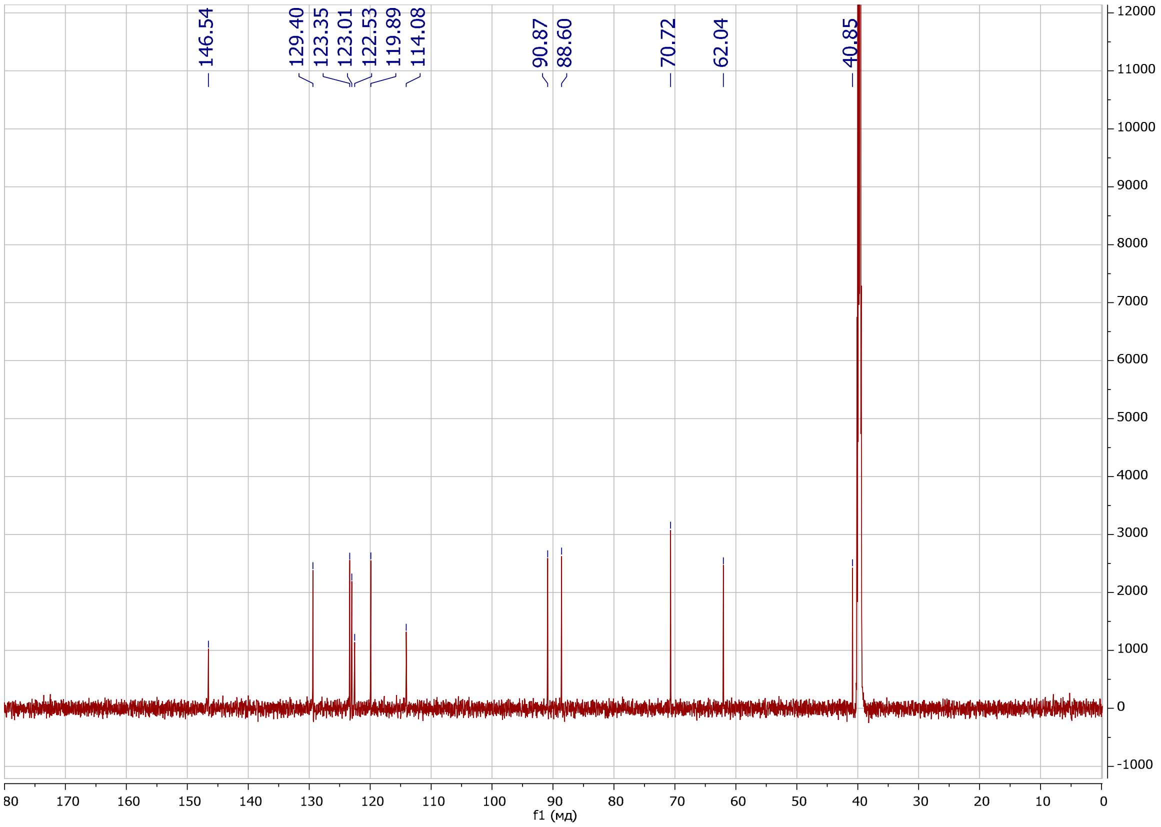

Figure S6. The ^{13}C NMR spectrum of N2-(β-D-2'deoxyribofuranosyl)-5-bromo-indazole (N2)**-7** (176 MHz, DMSO-d_6, 303 K).

Display Report

Analysis Info

		Acquisition Date	4/24/2026 3:34:59 PM
Analysis Name	D:\Data\Ikar\2026 april\strangeweek\5Br-Indz-2etdRib N2(12) 240426_34_01_6579.d		
Method	ik-deltasmall-1600-positive .m	Operator	BDAL@DE
Sample Name	5Br-Indz-2etdRib N2(12) 240426	Instrument	compact 8255754.20088
Comment			

Acquisition Parameter

Source Type	ESI	Ion Polarity	Positive	Set Nebulizer	0.4 Bar
Focus	Active	Set Capillary	4500 V	Set Dry Heater	180 °C
Scan Begin	50 m/z	Set End Plate Offset	-500 V	Set Dry Gas	4.0 l/min
Scan End	1600 m/z	Set Charging Voltage	2000 V	Set Divert Valve	Source
		Set Corona	0 nA	Set APCI Heater	0 °C

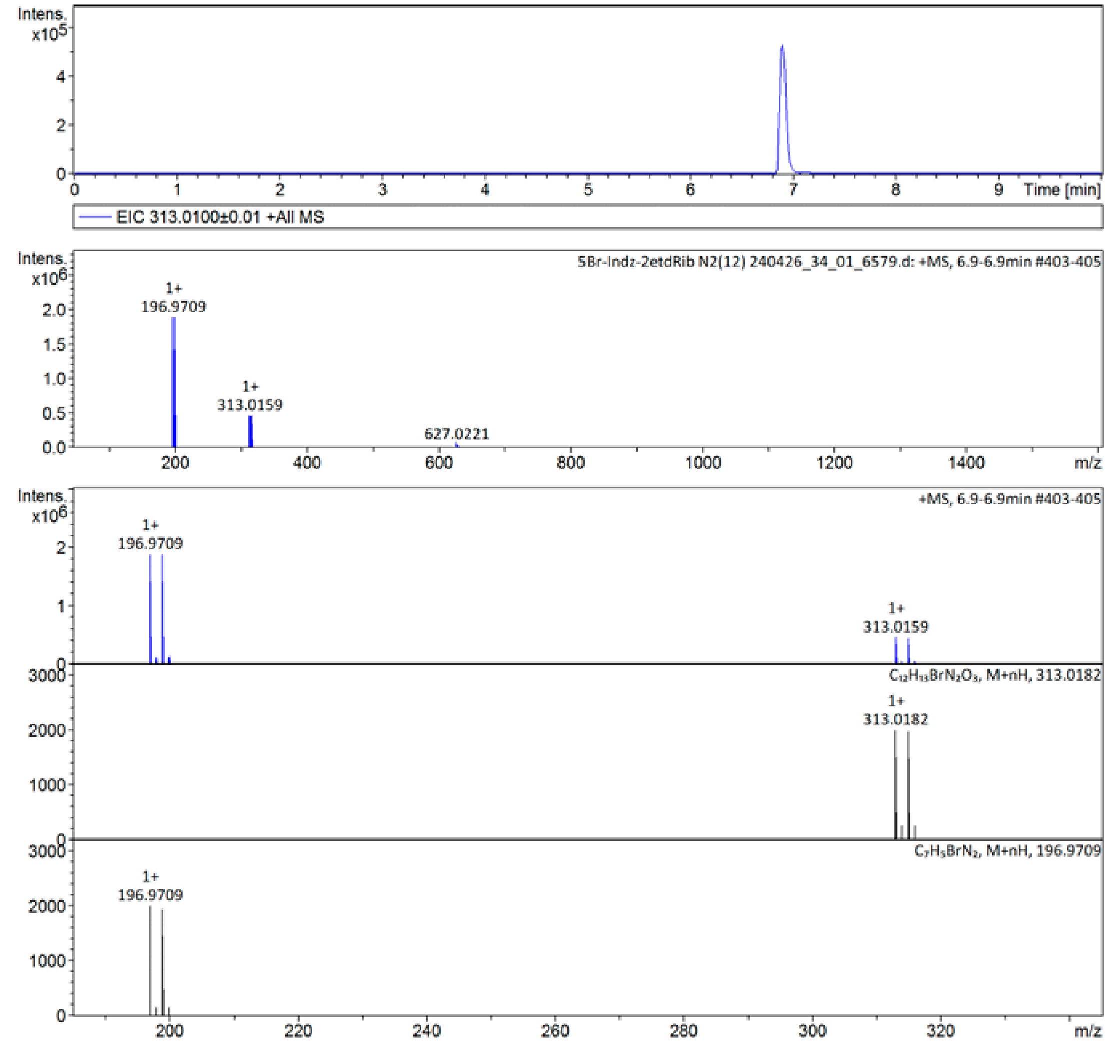

5Br-Indz-2etdRib N2(12) 240426_34_01_6579.d

Bruker Compass DataAnalysis 4.3 printed: 4/24/2026 3:54:29 PM by: BDAL@DE Page 1 of 1

Figure S7. The HRMS data of (N2)-**7**.

IBCH RAS

Project Name Defaults

Reported by User: Breeze user (Breeze)

Breeze 2 HPLC System

SAMPLE INFORMATION

Sample Name:	5-br-indz-2'dr purity pê 2	Acquired By:	Breeze
Sample Type:	Unknown	Date Acquired:	10.11.2025 14:33:03 GMT-4
Vial:	1	Acq. Method:	100B_dual_280nm_05ml
Injection #:	1	Date Processed:	10.11.2025 14:56:27 GMT-4
Injection Volume:	10.00 ul	Channel Name:	W2489 ChA
Run Time:	20.00 Minutes	Channel Desc.:	W2489 ChA 254nm
Column Type:		Sample Set Name	í

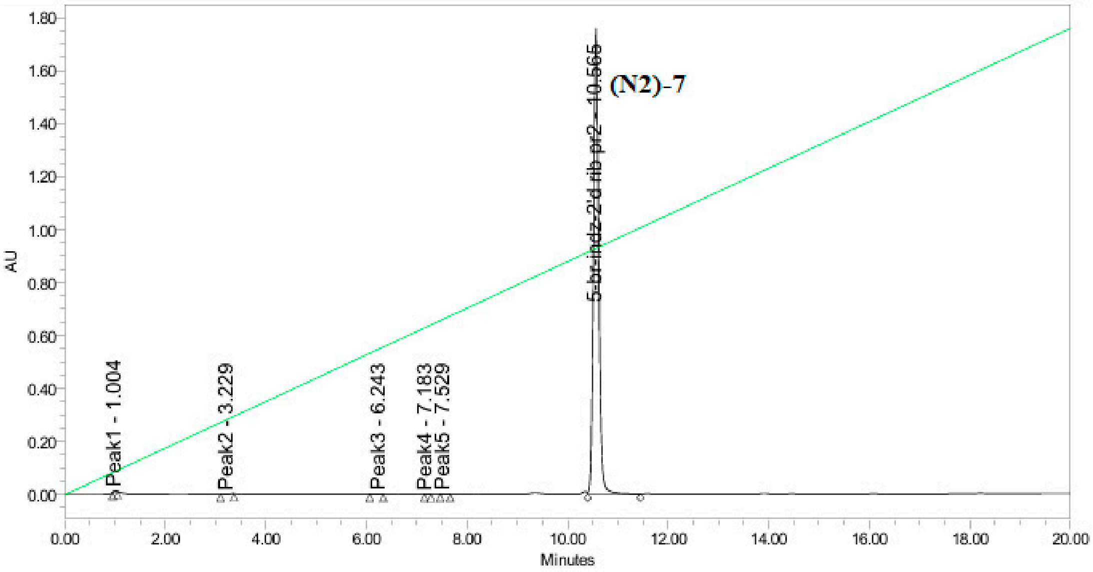

	Peak Name	RT (min)	Area (µV*sec)	% Area	Height (µV)	% Height
1	Peak1	1.004	18649	0.15	4928	0.28
2	Peak2	3.229	11227	0.09	-1149	0.07
3	Peak3	6.243	7441	0.06	681	0.04
4	Peak4	7.183	790	0.01	193	0.01
5	Peak5	7.529	4599	0.04	892	0.05
6	5-br-indz-2'd rib pr2	10.565	12566133	99.66	1750898	99.55

Report Method: Gradient Overlay Report ASC Printed: 10.11.2025

Page: 1 of 1 14:56:55 Etc/GMT-4

Figure S8. The HPLC data of (N2)-**7.**

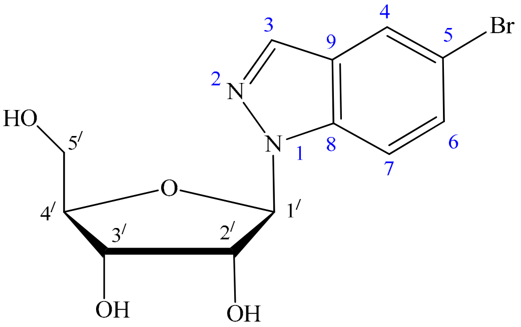

(N1)-**8**

N1-(β-D-ribofuranosyl)-5-bromo-indazole ((N1)-8).

^{1}H NMR: δ= 8.16, (s, 1H-weak, H-3 indazole), 8.03 (br.d., J = 1.65 Hz, 1H-weak, H-4 indazole), 7.81 (d., J = 8.24 Hz, 1H, H-7 indazole), 7.53 (br.dd, J = 1.93 Hz; 10.96 Hz, 1H, H-6 indazole), 6.09 (d, J = 4.54 Hz; 1H, H-1′), 5.35 (d, J = 5.58 Hz 1H, OH-2′), 5.13 (d, J = 5.06 Hz, 1H, OH-3′), 4.75 (t, J = 4.70 Hz; 14.46 Hz, 1H, OH-5′), 4.62 (q, J = 5.24 Hz; 15.09 Hz, 1H, H-2′), 4.21 (q, J = 5.01 Hz; 14.30 Hz, 1H, H-3′), 3.93 (q, J = 4.85 Hz; 14.30 Hz, 1H, H-4′), 3.56 (m, 1H, H_a-5′), 3.42 (m, 1H, H_b-5′).

^{13}C NMR: δ = 138.56 (C8 indazole), 133.75 (C3 indazole), 129.15 (C6 indazole), 125.58 (C9 indazole), 123.19 (C4 indazole), 113.26 (C5 indazole), 111.92 (C7 indazole), 90.14 (C1′), 84.90 (C4′), 73.19 (C2′), 70.55 (C3′), 62.05 (C5′).

^{15}N NMR: δ = 318.29 (N2), 196.13 (N1).

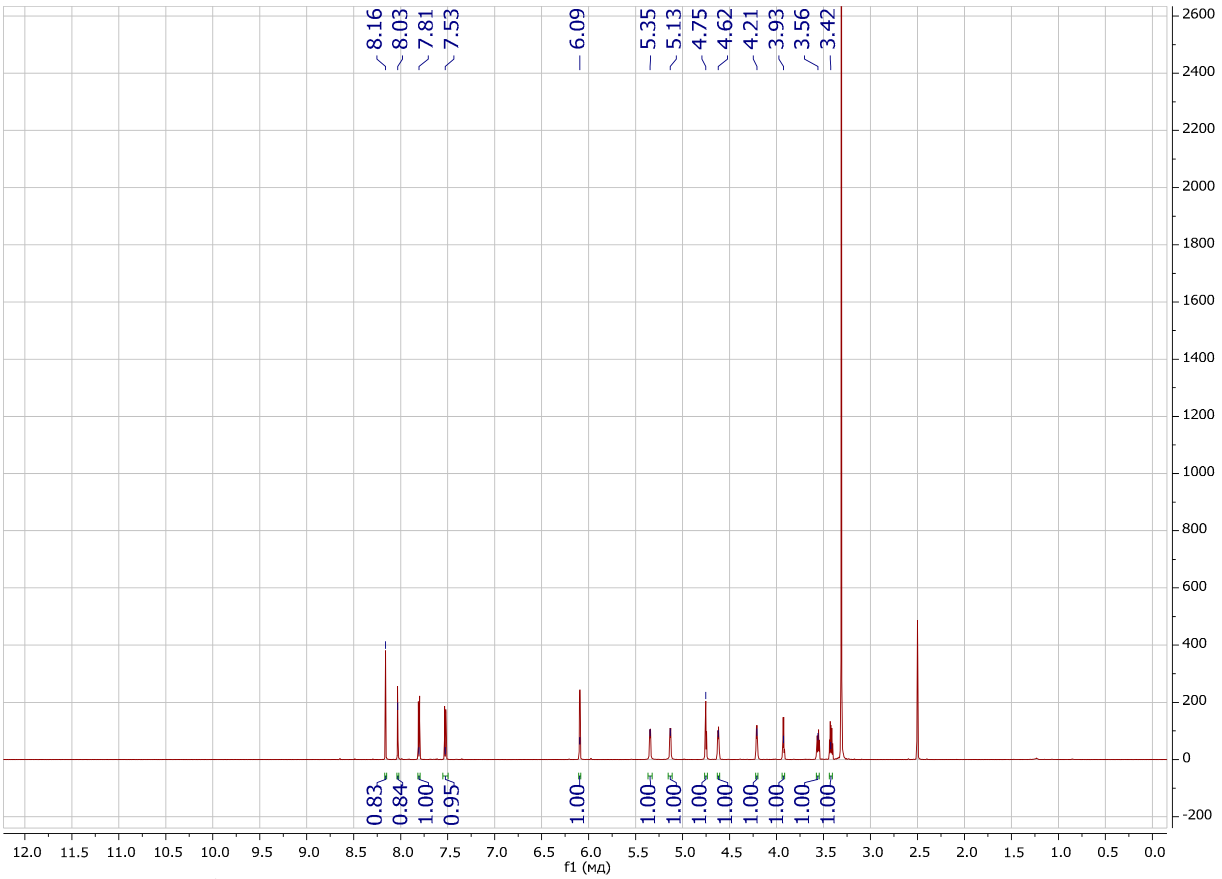

Figure S9. The ^{1}H NMR spectrum of N1-(β-D-ribofuranosyl)-5-bromo-indazole (N1)-**8** (700 MHz, DMSO-d_6, 303 K).

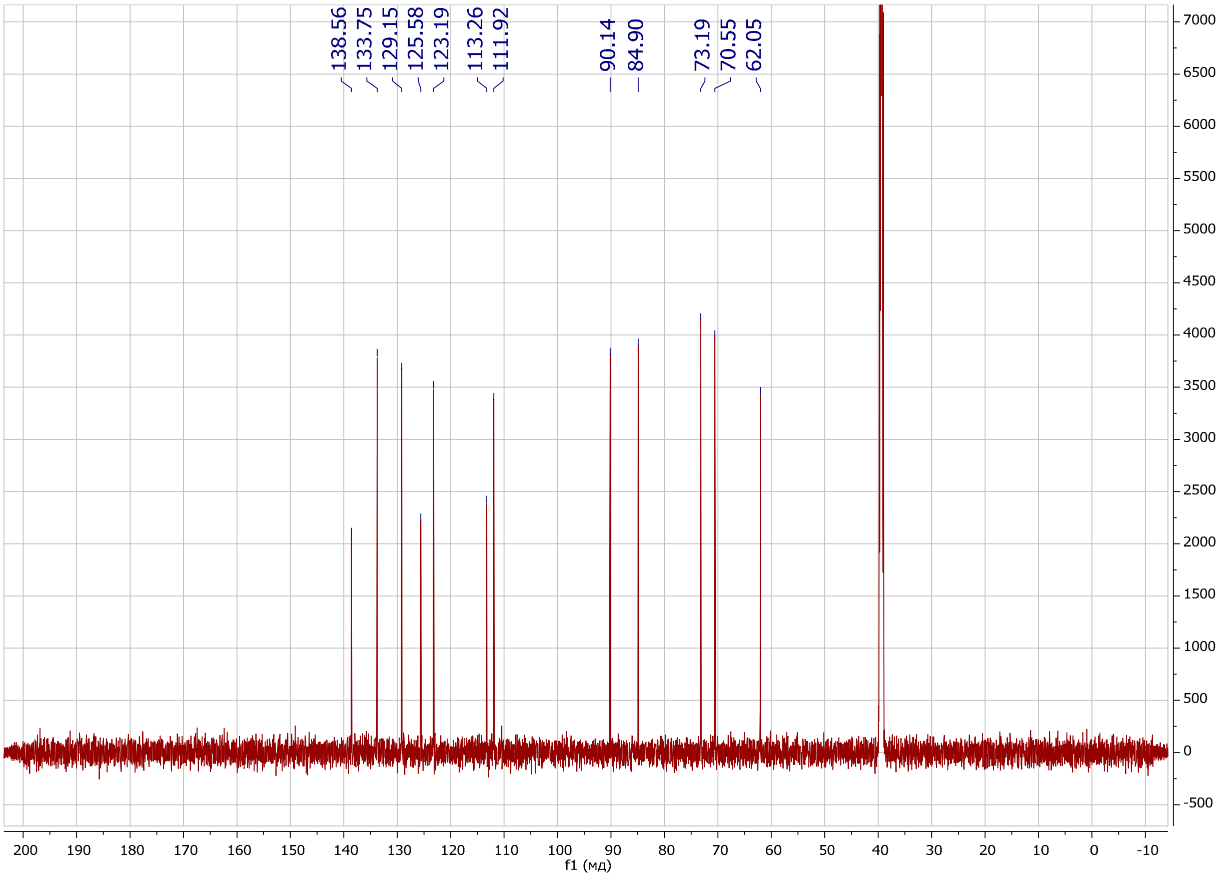

Figure S10. The ^{13}C NMR spectrum of N1-(β-D-ribofuranosyl)-5-bromo-indazole (N1)-**8** (176 MHz, DMSO-d_6, 303 K).

Display Report

Analysis Info

Analysis Name	D:\Data\Ikar\2026 april\strangeweek\5-Br-Indz-Rib N1 (7) 240426_25_01_6570.d	Acquisition Date	4/24/2026 1:06:00 PM
Method	ik-deltasmall-1600-positive .m	Operator	BDAL@DE
Sample Name	5-Br-Indz-Rib N1 (7) 240426	Instrument	compact 8255754.20088
Comment			

Acquisition Parameter

Source Type	ESI	Ion Polarity	Positive	Set Nebulizer	0.4 Bar
Focus	Active	Set Capillary	4500 V	Set Dry Heater	180 °C
Scan Begin	50 m/z	Set End Plate Offset	-500 V	Set Dry Gas	4.0 l/min
Scan End	1600 m/z	Set Charging Voltage	2000 V	Set Divert Valve	Source
		Set Corona	0 nA	Set APCI Heater	0 °C

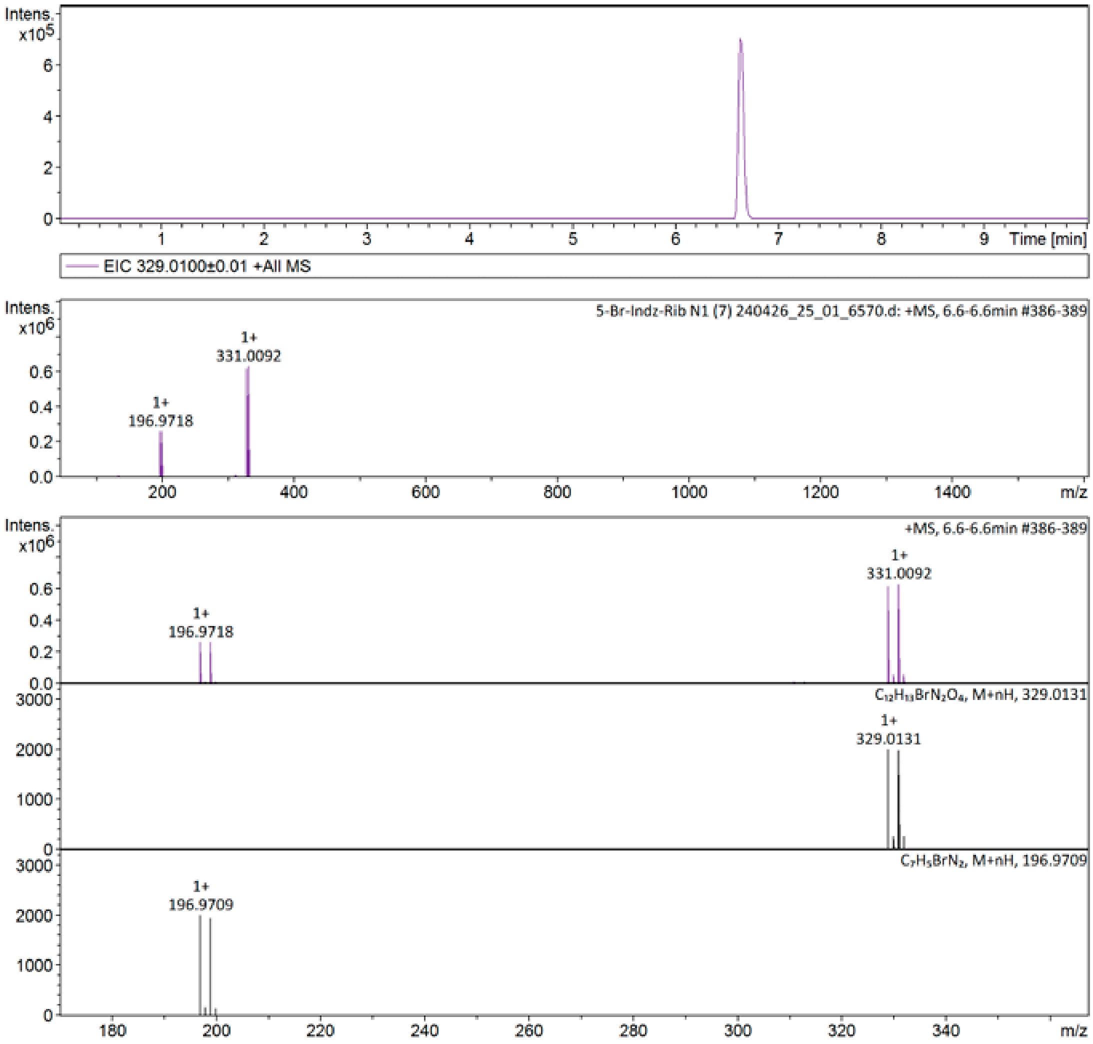

5-Br-Indz-Rib N1 (7) 240426_25_01_6570.d

Bruker Compass DataAnalysis 4.3 printed: 4/24/2026 1:57:43 PM by: BDAL@DE Page 1 of 1

Figure S11. The HMRS data of (N1)-**8.**

IBCH RAS
Project Name Fleximer
Reported by User: Breeze user (Breeze)

Breeze 2 HPLC System

SAMPLE INFORMATION

Sample Name:	5-br-indz-rib prod 1 purity	Acquired By:	Breeze
Sample Type:	Unknown	Date Acquired:	19.08.2025 19:01:40 GMT-4
Vial:	1	Acq. Method:	100B_dual_280nm_05ml
Injection #:	1	Date Processed:	19.08.2025 19:32:45 GMT-4
Injection Volume:	10.00 ul	Channel Name:	W2489 ChA
Run Time:	20.00 Minutes	Channel Desc.:	W2489 ChA 254nm
Column Type:		Sample Set Name	m

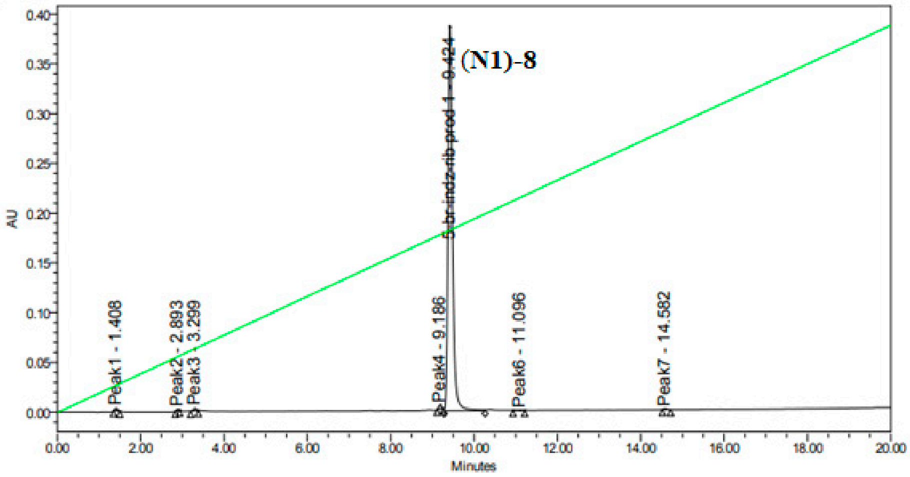

	Peak Name	RT (min)	Area (µV*sec)	% Area	Height (µV)	% Height
1	Peak1	1.408	11169	0.39	2156	0.54
2	Peak2	2.893	3988	0.14	1046	0.26
3	Peak3	3.299	11899	0.41	2345	0.59
4	Peak4	9.186	17399	0.60	3726	0.93
5	5-br-indz-rib prod 1	9.424	2850220	98.34	390195	97.54
6	Peak6	11.096	603	0.02	-50	0.01
7	Peak7	14.582	3053	0.11	511	0.13

Report Method: Gradient Overlay Report ASC
Page: 1 of 1

Printed: 19.08.2025
19:33:44 Etc/GMT-4

Figure S12. The HPLC data of (N1)-**8.**

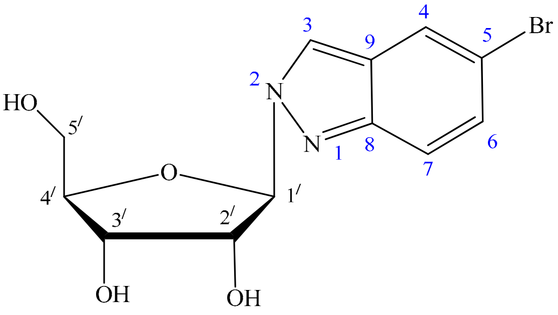

(N2)-**8**

N2-(β-D-ribofuranosyl)-5-bromo-indazole ((N2)-8).

^{1}H NMR: δ= 8.65, (s, 1H, H-3 indazole), 8.00 (br.d., *J* = 1.40 Hz, 1H-weak, H-4 indazole), 7.62 (d., *J* = 9.12 Hz, 1H, H-7 indazole), 7.35 (dd, *J* = 1.99 Hz; 10.77 Hz, 1H, H-6 indazole), 5.98 (d, *J* = 3.64 Hz; 1H, H-1′), 5.58 (m, 1H, OH-2′),

5.20 (m, 1H, OH-3′), 5.00 (t, *J* = 5.27 Hz; 10.17 Hz, 1H, OH-5′), 4.39 (m, 1H, H-2′), 4.19 (m, 1H, H-3′), 4.02 (q, *J* = 4.72 Hz; 13.61 Hz, 1H, H-4′), 3.71 (m, 1H, H_a-5′), 3.57 (m, 1H, H_b-5′).

^{13}C NMR: δ = 146.07 (C8 indazole), 128.88 (C6 indazole), 122.74 (C4 indazole), 122.69 (C3 indazole), 121.91 (C9 indazole), 119.27 (C7 indazole), 113.55 (C5 indazole), 94.54 (C1′), 85.08 (C4′), 75.18 (C2′), 69.78 (C3′), 61.01 (C5′).

^{15}N NMR: δ = 239.51 (N2).

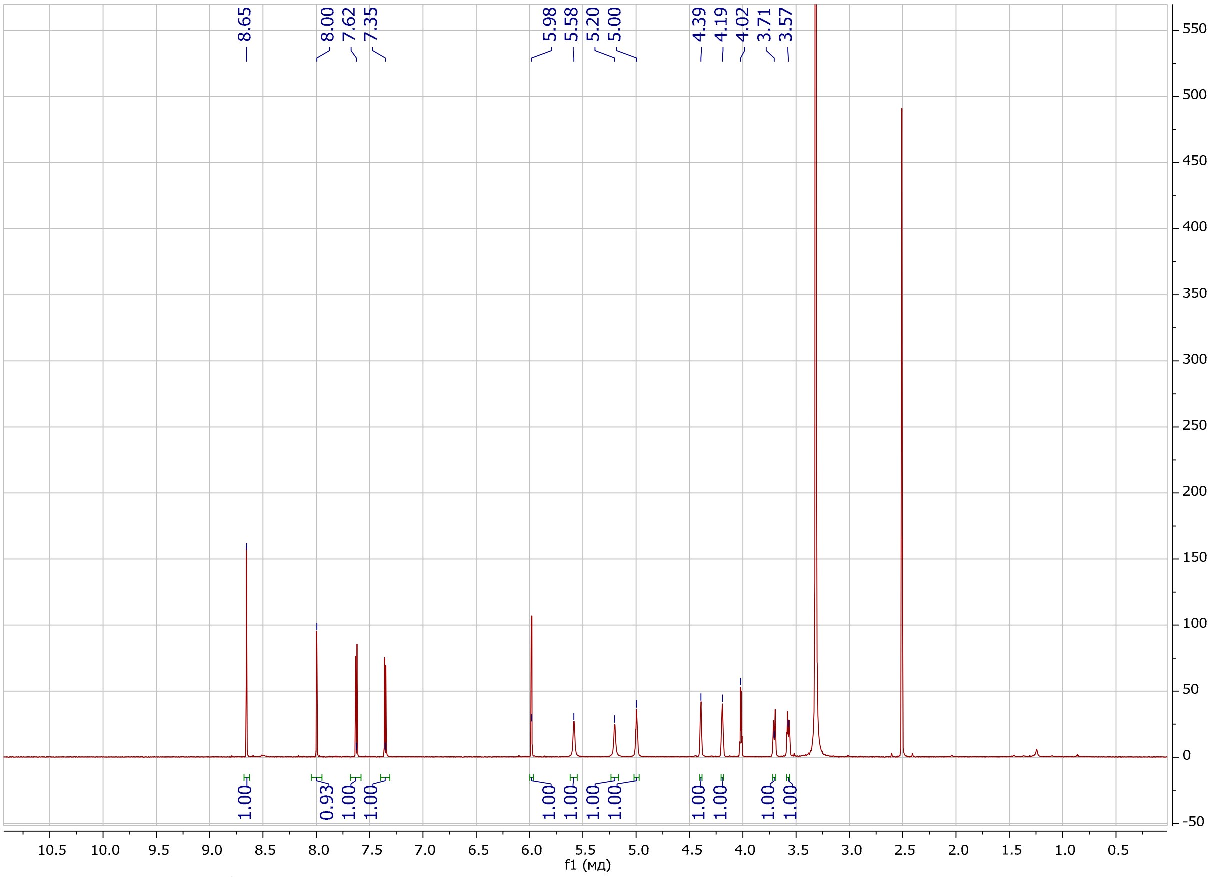

Figure S13. The ^{1}H NMR spectrum of N2-(β-D-ribofuranosyl)-5-bromo-indazole (N2)-**8** (700 MHz, DMSO-d_6, 303 K).

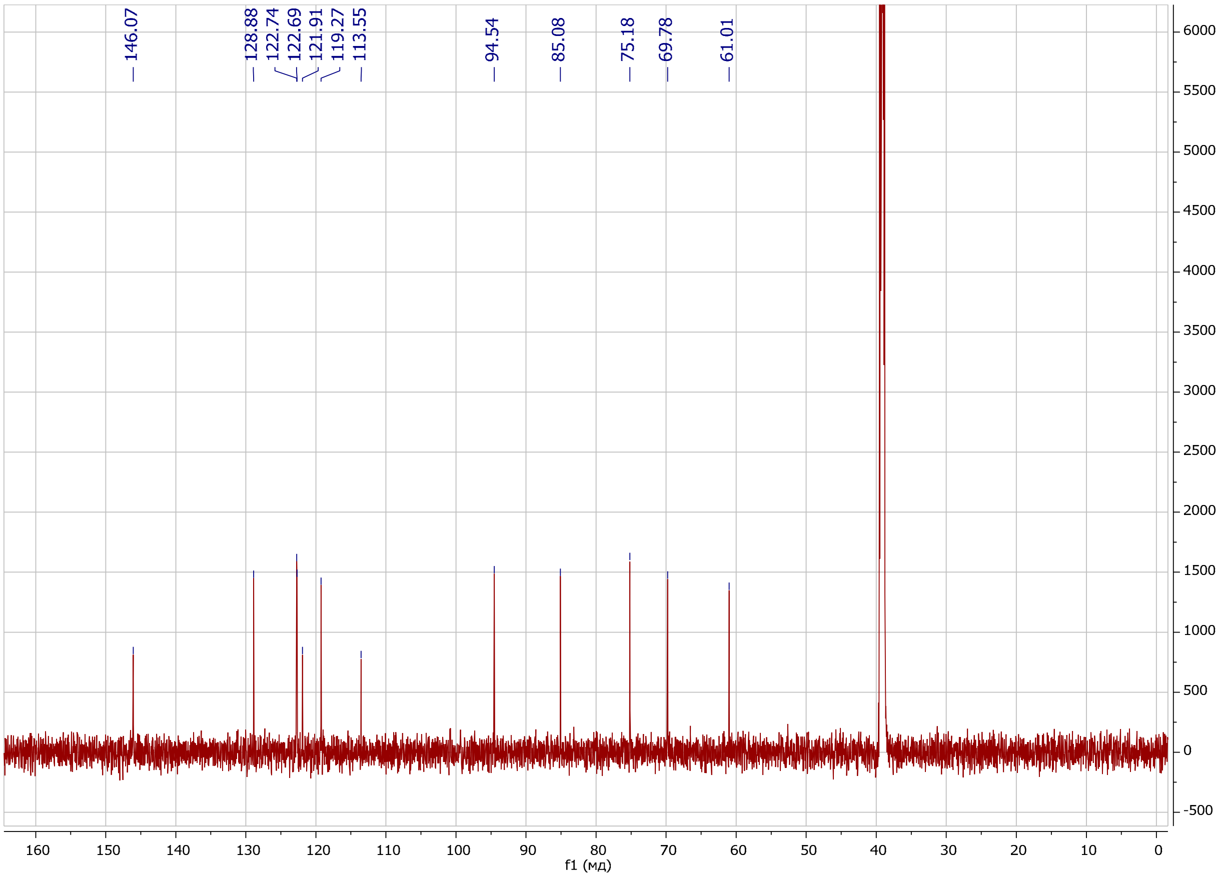

Figure S14. The ^{13}C NMR spectrum of N2-(β-D-ribofuranosyl)-5-bromo-indazole (N2)-**8** (176 MHz, DMSO-d_6, 303 K).

Display Report

Analysis Info

Analysis Name	D:\Data\Ikar\2026 april\strangeweek\5-Br-Indz-Rib N2(11) 240426_27_01_6572.d	Acquisition Date	4/24/2026 1:38:57 PM
Method	ik-deltasmall-1600-positive .m	Operator	BDAL@DE
Sample Name	5-Br-Indz-Rib N2(11) 240426	Instrument	compact 8255754.20088
Comment			

Acquisition Parameter

Source Type	ESI	Ion Polarity	Positive	Set Nebulizer	0.4 Bar
Focus	Active	Set Capillary	4500 V	Set Dry Heater	180 °C
Scan Begin	50 m/z	Set End Plate Offset	-500 V	Set Dry Gas	4.0 l/min
Scan End	1600 m/z	Set Charging Voltage	2000 V	Set Divert Valve	Source
		Set Corona	0 nA	Set APCI Heater	0 °C

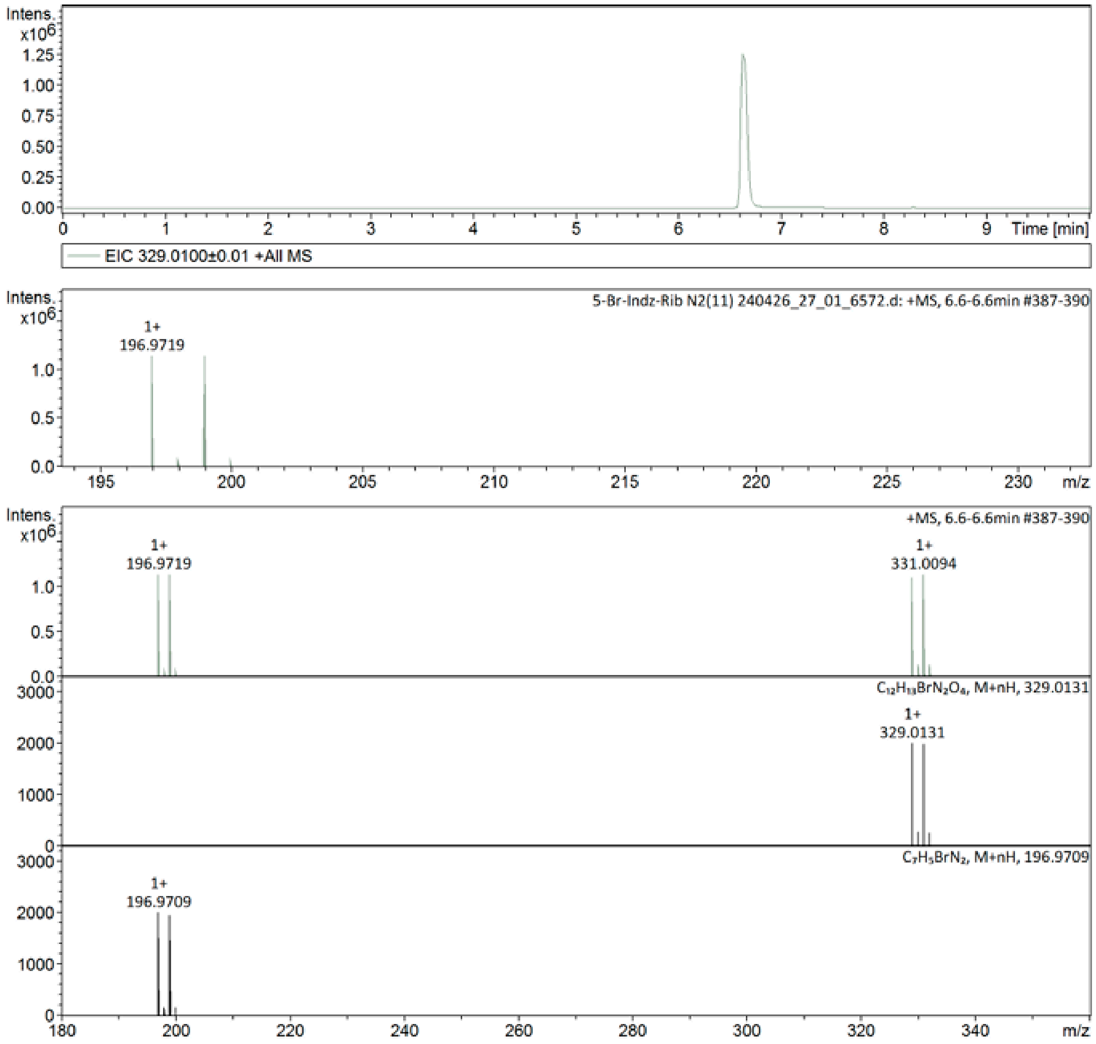

5-Br-Indz-Rib N2(11) 240426_27_01_6572.d

Bruker Compass DataAnalysis 4.3 printed: 4/24/2026 2:09:23 PM by: BDAL@DE Page 1 of 1

Figure S15. The HMRS data of (N2)-**8**.

IBCH RAS

Project Name Defaults

Reported by User: Breeze user (Breeze)

Breeze 2 HPLC System

SAMPLE INFORMATION

Sample Name:	5-Br-indz-rib prod II purity	Acquired By:	Breeze
Sample Type:	Unknown	Date Acquired:	19.09.2025 15:31:02 GMT-4
Vial:	1	Acq. Method:	100B_dual_280nm_05ml
Injection #:	1	Date Processed:	19.09.2025 15:56:12 GMT-4
Injection Volume:	5.00 ul	Channel Name:	W2489 ChB
Run Time:	20.00 Minutes	Channel Desc.:	W2489 ChB 280nm
Column Type:		Sample Set Name	5

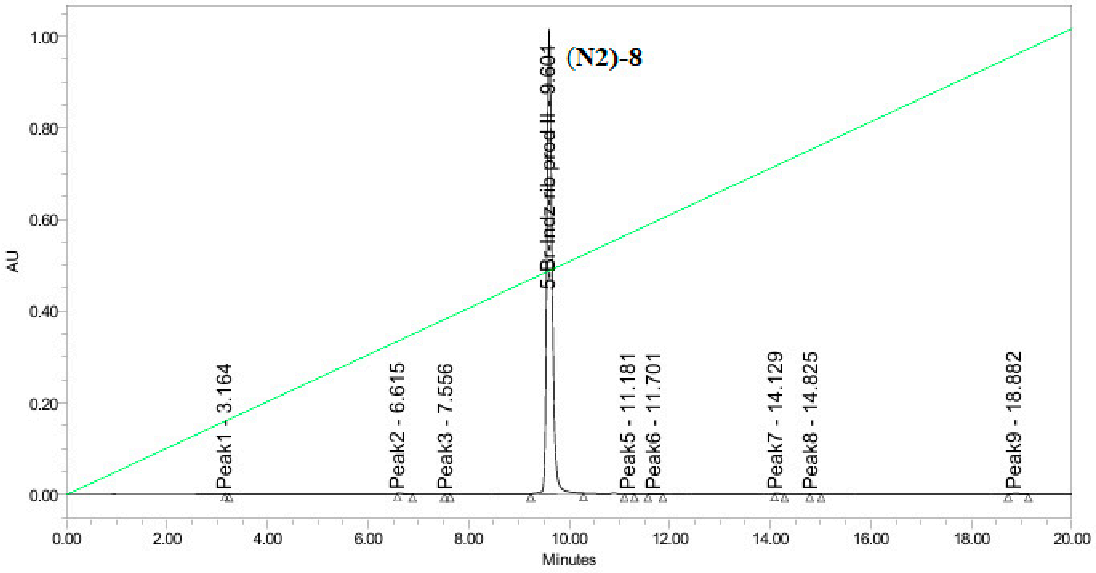

	Peak Name	RT (min)	Area (µV*sec)	% Area	Height (µV)	% Height
1	Peak1	3.164	926	0.01	219	0.02
2	Peak2	6.615	3002	0.04	584	0.06
3	Peak3	7.556	1627	0.02	388	0.04
4	5-Br-Indz-rib prod II	9.601	7268400	99.65	1010018	99.61
5	Peak5	11.181	998	0.01	167	0.02
6	Peak6	11.701	1785	0.02	235	0.02
7	Peak7	14.129	3274	0.04	631	0.06
8	Peak8	14.825	1296	0.02	235	0.02

Report Method: Gradient Overlay Report ASC Printed: 19.09.2025 15:56:38 Etc/GMT-4

Page: 1 of 2

Figure S16. The HPLS data of (N2)-**8**.

N1-(β-D-2'deoxyribofuranosyl)-6-bromo-indazole ((N1)-9).
N2-(β-D-2'deoxyribofuranosyl)-6-bromo-indazole ((N2)-9).

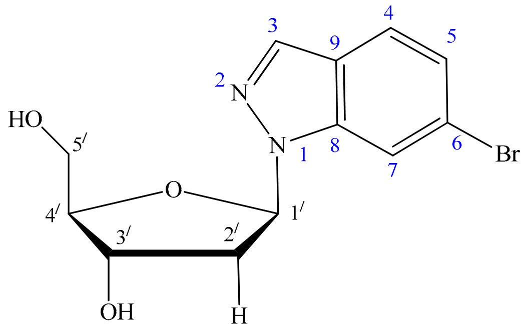

(N1)-9

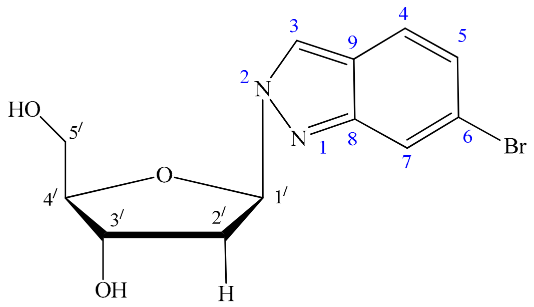

(N2)-9

^{1}H NMR: δ=8.64, (s, 1H-0.25, H-3 indazole **N2-isomer**), 8.17 (s, 1H-0.75, H-3 indazole **N1-isomer**), 8.13 (s., 1H-0.91, H-7 indazole **N1-isomer**),7.88 (s, 1H-0.21, H-7 indazole **N2-isomer**), 7.73 (d., *J* = 8.52 Hz, 1H-0.87, H-4 indazole **N1-isomer**), 7.71 (d., *J* = 9.11 Hz, 1H-0.24, H-4 indazole **N2-isomer**), 7.31 (br.dd, *J* = 1.50 Hz; 10.11 Hz, 1H-0.87 , H-5 indazole **N1-isomer**), 7.15 (br.dd, *J* = 1.69 Hz; 10.56 Hz, 1H-0.87 , H-5 indazole **N2-isomer**), 6.59 (t, *J* = 6.29 Hz; 12.78 Hz, 1H, H-1′ **N1-isomer**), 6.38 (t, *J* = 5.96 Hz; 12.10 Hz, 1H-0.24, H-1′ **N2-isomer**), 5.31 (s., 1H-0.25, OH-3′ **N2-isomer**),), 5.25 (s., 1H, OH-3′ **N1-isomer**), 4.91 (s, 1H-0.25, H-5′3′ **N2-isomer**), 4.70 (m, 1H, H-5′ **N1-isomer**), 4.45 (m, 1H, H-3′ **N1-isomer**), 4.41 (m, 1H-0.25, H-3′ **N2-isomer**), 3.92 (m, 1H-0.26, H-4′ **N2-isomer**), 3.82 (m, 1H, H-4′ **N1-isomer**), 3.60 (m, 1H-0.26, H_a-5′ **N2-isomer**), 3.50 (m, 1H-0.25, H_b-5′ **N2-isomer** overlapped by the signal 3.48 **N1-isomer**), 3.48 (m, 1H, H_a-5′ **N1-isomer**), 3.32 (m, 1H, H_b-5′ overlapped by the water signal **N1-isomer**), 2.88 (m, 1H, H_a-2′ **N1-isomer**),), 2.65 (m, 1H-0.25, H_a-2′ **N2-isomer**), 2.39 (m, 1H-0.25, H_b-2′ **N2-isomer**). 2.27 (m, 1H, H_b-2′ **N1-isomer**).

^{13}C NMR (**N1-isomer**): δ = 140.93 (C8 indazole), 134.96 (C3 indazole), 123.52 (C5 indazole), 122.95 (C9 indazole), 123.21 (C4 indazole), 120.67 (C6 indazole), 113.49 (C7 indazole), 88.03 (C4′), 86.29 (C1′), 71.45 (C3′), 62.80 (C5′), 38.79 (C2′).

^{13}C NMR (**N2-isomer**): δ = 148.30 (C8 indazole), 129.83 (C6 indazole), 124.34 (C5 indazole), 123.52 (C4 indazole), 122.95 (C3 indazole), 119.18 (C9 indazole), 119.09 (C7 indazole), 90.38 (C1′), 88.10 (C4′), 70.19 (C3′), 61.53 (C5′), 40.35 (C2′).

^{15}N NMR: δ = 316.62 (N2 **N1-isomer**), 243.84 (N2 **N2-isomer**), 198.89 (N1 **N1-isomer**).

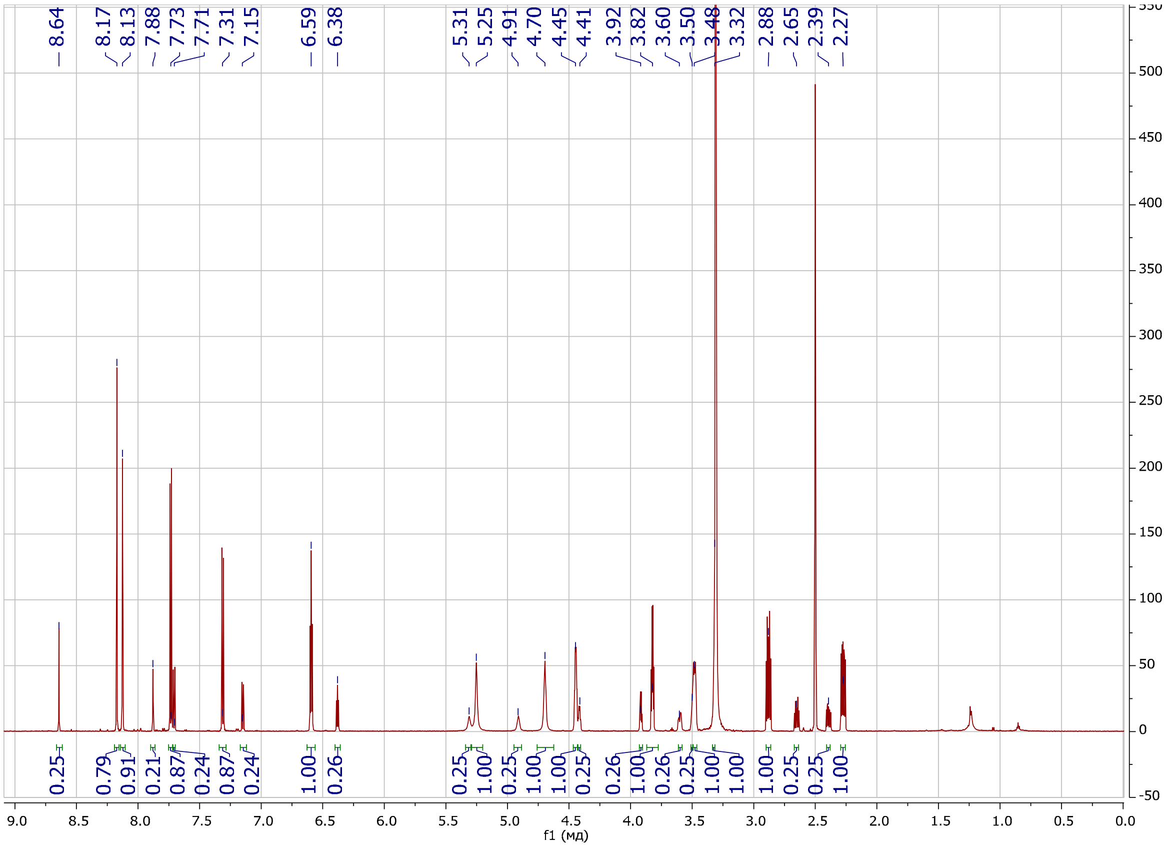

Figure S17. The ^{1}H NMR spectrum of N1-(β-D-2'deoxyribofuranosyl)-6-bromo-indazole (N1)-**9** and N2-(β-D-2'deoxyribofuranosyl)-6-bromo-indazole (N2)-**9** (700 MHz, DMSO-d_6, 303 K).

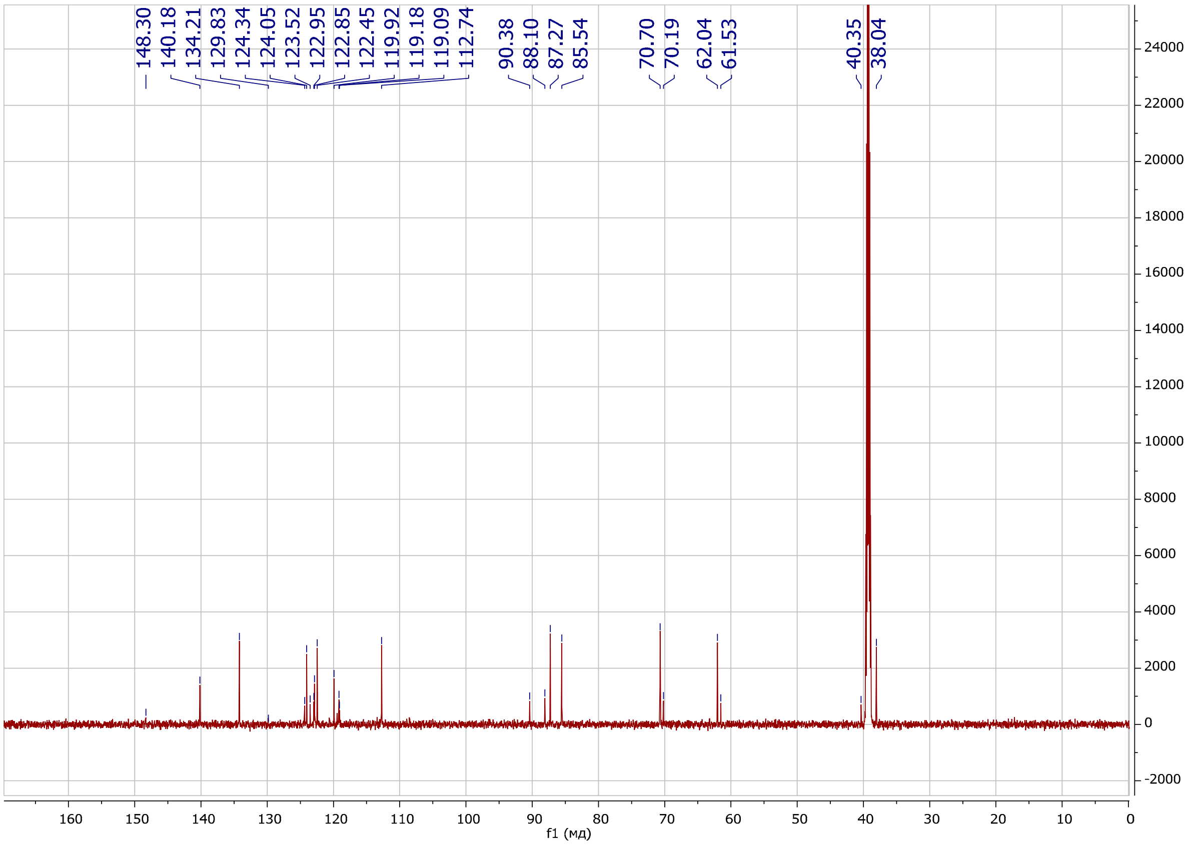

Figure S18. The ^{13}C NMR spectrum of N1-(β-D-2'deoxyribofuranosyl)-6-bromo-indazole (N1)-**9** and N2-(β-D-2'deoxyribofuranosyl)-6-bromo-indazole (N2)-**9** (176 MHz, DMSO-d_6, 303 K).

Display Report

Analysis Info

Analysis Name	D:\Data\Ikar\2026 april\strangeweek\6BrIndz2etdRib N12 (10) 240426_33_01_6578.d	Acquisition Date	4/24/2026 3:18:31 PM
Method	ik-deltasmall-1600-positive .m	Operator	BDAL@DE
Sample Name	6BrIndz2etdRib N12 (10) 240426	Instrument	compact 8255754.20088
Comment			

Acquisition Parameter

Source Type	ESI	Ion Polarity	Positive	Set Nebulizer	0.4 Bar
Focus	Active	Set Capillary	4500 V	Set Dry Heater	180 °C
Scan Begin	50 m/z	Set End Plate Offset	-500 V	Set Dry Gas	4.0 l/min
Scan End	1600 m/z	Set Charging Voltage	2000 V	Set Divert Valve	Source
		Set Corona	0 nA	Set APCI Heater	0 °C

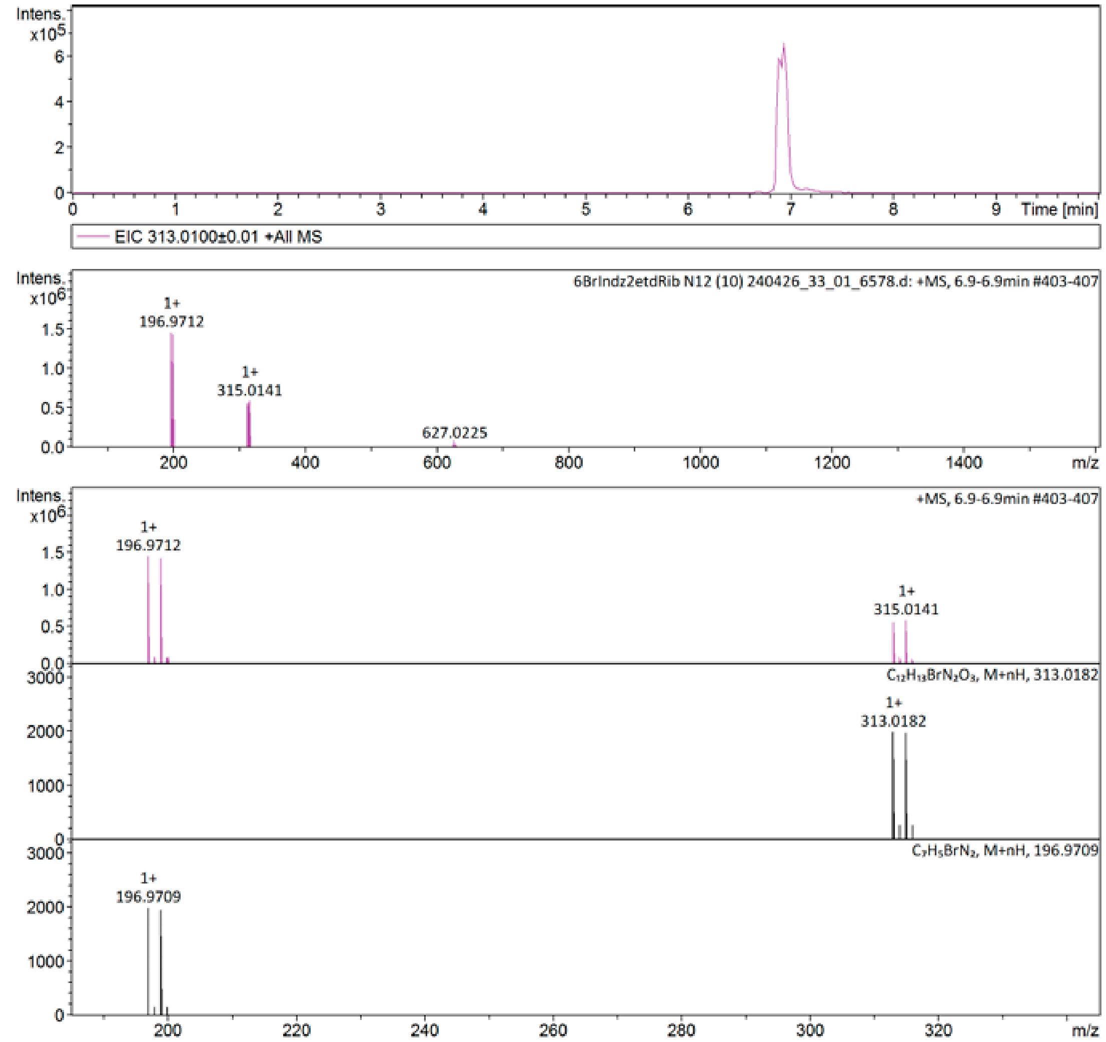

6BrIndz2etdRib N12 (10) 240426_33_01_6578.d

Bruker Compass DataAnalysis 4.3 printed: 4/24/2026 3:41:40 PM by: BDAL@DE Page 1 of 1

Figure S19. The HMRS data of (N1)-**9**+(N2)-**9**.

IBCH RAS

Project Name Flexímer

Reported by User: Breeze user (Breeze)

Breeze 2 HPLC System

SAMPLE INFORMATION

Sample Name:	6-br-indz-2'drib purity	Acquired By:	Breeze
Sample Type:	Unknown	Date Acquired:	18.08.2025 15:02:55 GMT-4
Vial:	1	Acq. Method:	100B_dual_280nm_05ml
Injection #:	1	Date Processed:	18.08.2025 19:07:23 GMT-4
Injection Volume:	10.00 ul	Channel Name:	W2489 ChB
Run Time:	20.00 Minutes	Channel Desc.:	W2489 ChB 280nm
Column Type:		Sample Set Name	v

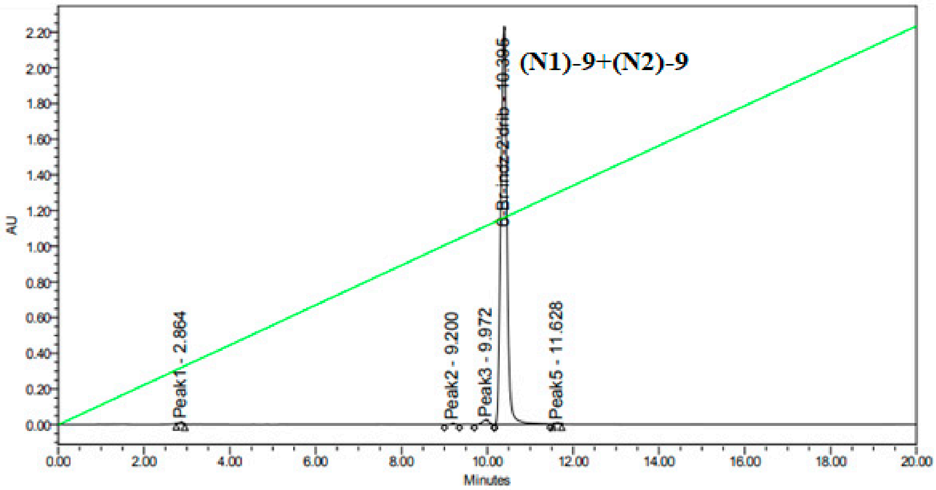

	Peak Name	RT (min)	Area (µV*sec)	% Area	Height (µV)	% Height
1	Peak1	2.864	62965	0.28	10238	0.45
2	Peak2	9.200	41070	0.18	5567	0.24
3	Peak3	9.972	275871	1.24	26232	1.15
4	6-Br-indz-2'drib	10.395	21819171	98.12	2232373	97.89
5	Peak5	11.628	38211	0.17	6085	0.27

Report Method: Gradient Overlay Report ASC

Printed: 18.08.2025 19:07:46 Etc/GMT-4

Page: 1 of 1

Figure S20. The chromatogram of (N1)-**9**+(N2)-**9.**

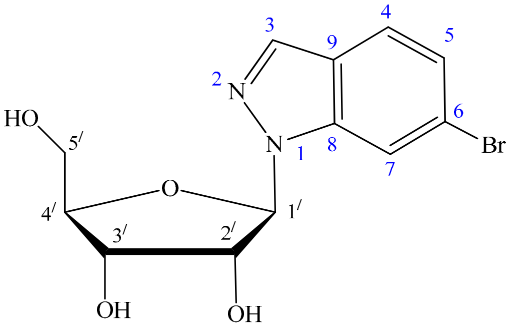

(N1)-**10**

N1-(β-D-ribofuranosyl)-6-bromo-indazole ((N1)-10).

^{1}H NMR: δ = 8.20, (s, 1H-weak, H-3 indazole),8.16 (br.s., 1H-weak, H-7 indazole), 7.75 (d., *J* = 8.65 Hz, 1H-weak, H-4 indazole), 7.33 (br.dd, *J* = 1.45 Hz; 10.07 Hz, 1H-weak, H-6 indazole), 6.12 (d, *J* = 4.56 Hz; 1H, H-1′), 5.32 (s, 1H, OH-2′), 5.11 (s, Hz, 1H, OH-3′), 4.78 (t, *J* = 5.64 Hz; 11.00 Hz, 1H, OH-5′), 4.60 (m, 1H, H-2′), 4.21 (m, 1H, H-3′), 3.94 (q, *J* = 4.61 Hz; 14.50 Hz, 1H, H-4′), 3.57 (m, 1H, H_a-5′), 3.43 (m, 1H, H_b-5′).

^{13}C NMR: δ = 140.72 (C8 indazole), 134.86 (C3 indazole), 124.48 (C5 indazole), 123.18 (C9 indazole), 122.85 (C4 indazole), 120.40 (C6 indazole), 113.09 (C7 indazole), 90.51 (C1′), 85.11 (C4′), 73.41 (C2′), 70.78 (C3′), 62.28 (C5′).

^{15}N NMR: δ = 316.65 (N2), 199.48 (N1).

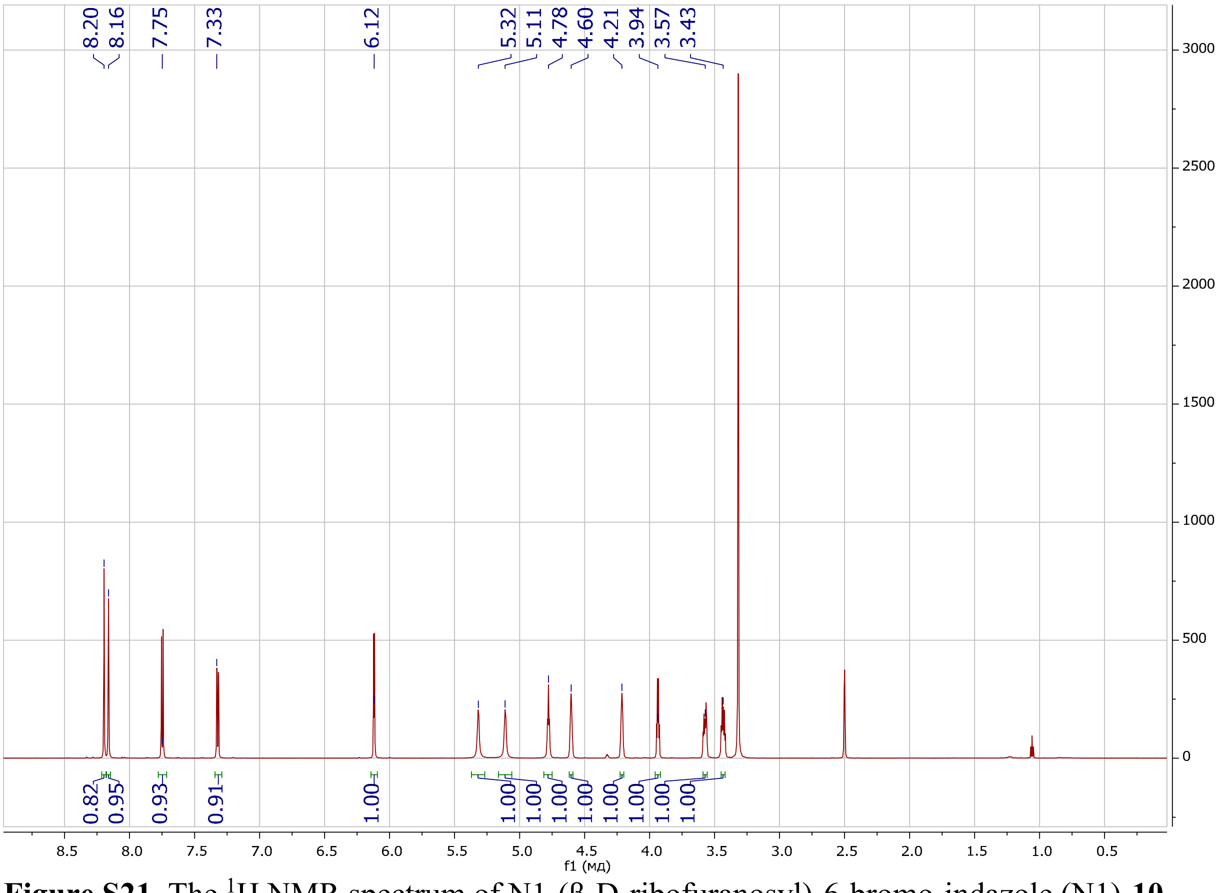

Figure S21. The ^{1}H NMR spectrum of N1-(β-D-ribofuranosyl)-6-bromo-indazole (N1)-**10** (700 MHz, DMSO-d_6, 303 K).

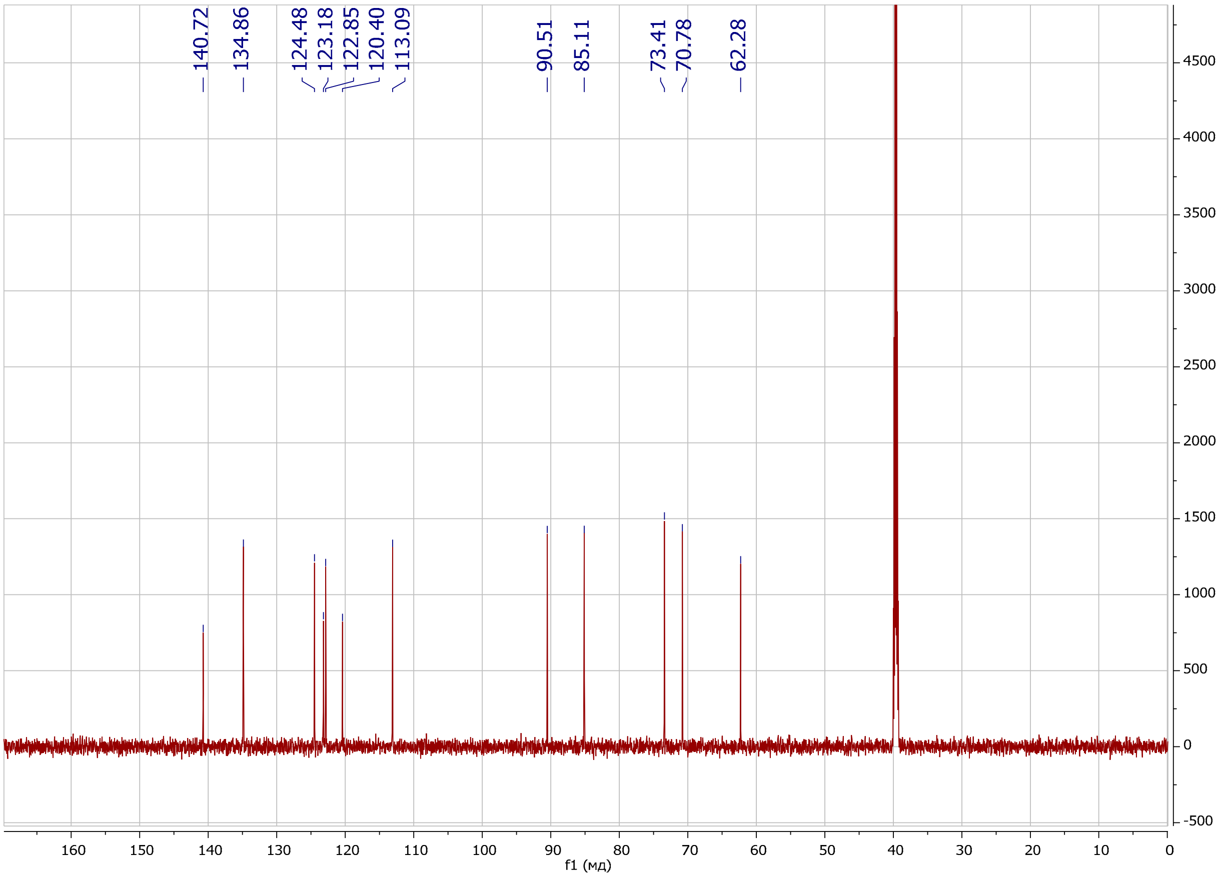

Figure S22. The ^{13}C NMR spectrum of N1-(β-D-ribofuranosyl)-6-bromo-indazole (N1)-**10** (176 MHz, DMSO-d_6, 303 K).

Display Report

Analysis Info

		Acquisition Date	4/24/2026 1:22:31 PM
Analysis Name	D:\Data\Ikar\2026 april\strangeweek\6-Br-Indz-Rib N1(9) 240426_26_01_6571.d		
Method	ik-deltasmall-1600-positive .m	Operator	BDAL@DE
Sample Name	6-Br-Indz-Rib N1(9) 240426	Instrument	compact 8255754.20088
Comment			

Acquisition Parameter

Source Type	ESI	Ion Polarity	Positive	Set Nebulizer	0.4 Bar
Focus	Active	Set Capillary	4500 V	Set Dry Heater	180 °C
Scan Begin	50 m/z	Set End Plate Offset	-500 V	Set Dry Gas	4.0 l/min
Scan End	1600 m/z	Set Charging Voltage	2000 V	Set Divert Valve	Source
		Set Corona	0 nA	Set APCI Heater	0 °C

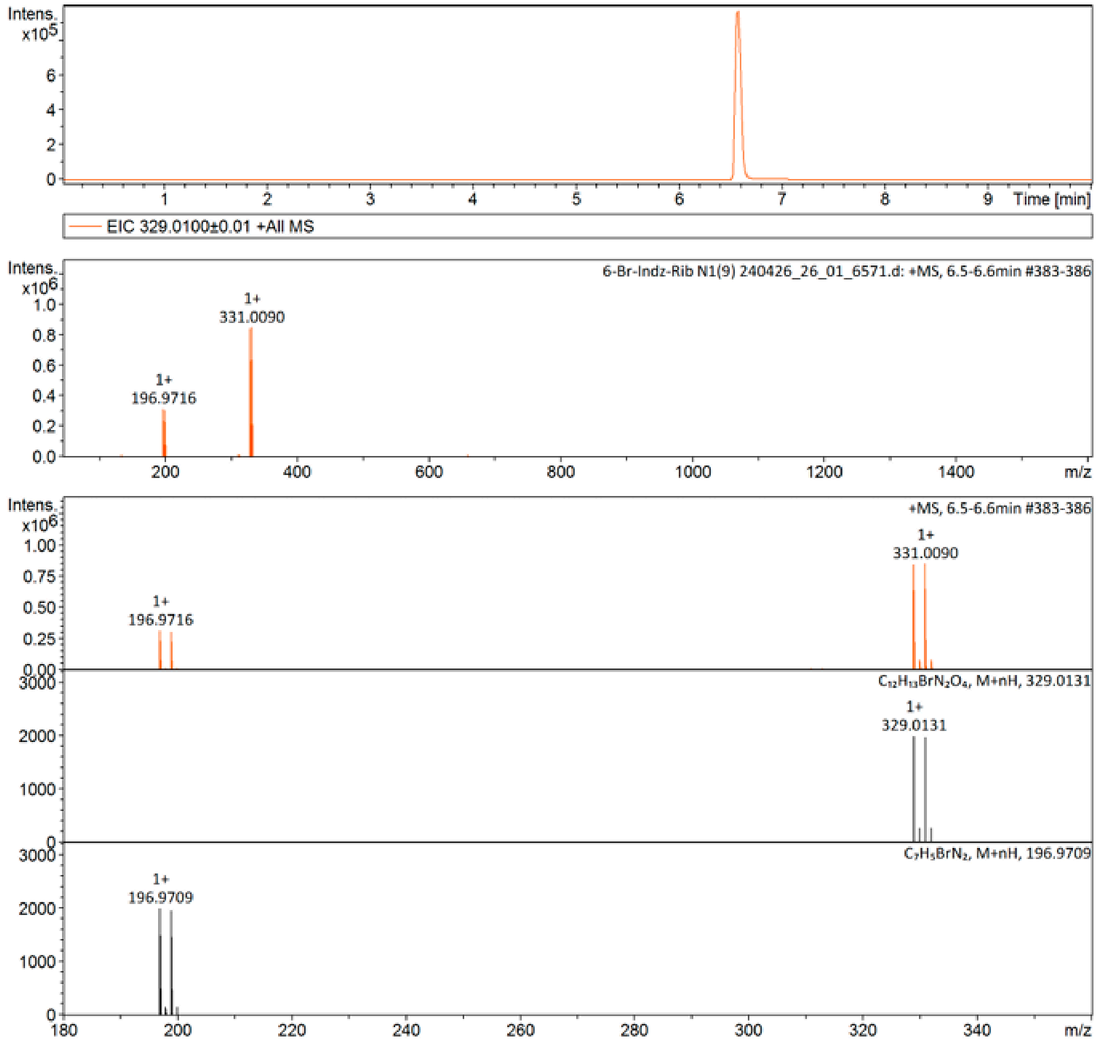

6-Br-Indz-Rib N1(9) 240426_26_01_6571.d

Bruker Compass DataAnalysis 4.3 printed: 4/24/2026 2:03:13 PM by: BDAL@DE Page 1 of 1

Figure S23. The HMRS data of (N1)-**10**.

IBCH RAS

Project Name Fleximer

Reported by User: Breeze user (Breeze)

Breeze 2 HPLC System

SAMPLE INFORMATION

Sample Name:	6-br-indz-rib prod 1 purity	Acquired By:	Breeze
Sample Type:	Unknown	Date Acquired:	18.08.2025 14:31:03 GMT-4
Vial:	1	Acq. Method:	100B_dual_280nm_05ml
Injection #:	1	Date Processed:	19.08.2025 19:28:23 GMT-4
Injection Volume:	10.00 ul	Channel Name:	W2489 ChB
Run Time:	20.00 Minutes	Channel Desc.:	W2489 ChB 280nm
Column Type:		Sample Set Name	i

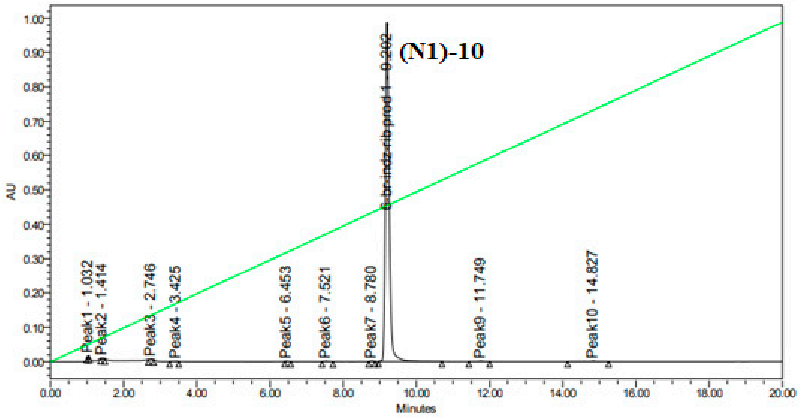

	Peak Name	RT (min)	Area (µV*sec)	% Area	Height (µV)	% Height
1	Peak1	1.032	12982	0.18	5220	0.52
2	Peak2	1.414	7142	0.10	1584	0.16
3	Peak3	2.746	10447	0.14	1873	0.19
4	Peak4	3.425	3417	0.05	442	0.04
5	Peak5	6.453	2003	0.03	385	0.04
6	Peak6	7.521	5546	0.08	766	0.08
7	Peak7	8.780	4984	0.07	915	0.09
8	6-br-indz-rib prod 1	9.202	7145522	99.12	982686	98.69

Report Method: Gradient Overlay Report ASC Printed: 19.08.2025

Page: 1 of 1 19:28:53 Etc/GMT-4

Figure S24. The HPLS data of (N1)-**10**.

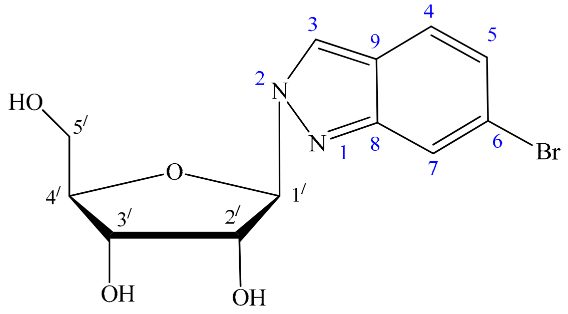

(N2)-**10**

N2-(β-D-ribofuranosyl)-6-bromo-indazole ((N2)-10)

^{1}H NMR: δ= 8.72, (s, 1H, H-3 indazole), 7.90 (s., 1H-weak, H-7 indazole), 7.72 (d., J = 8.87 Hz, 1H, H-4 indazole), 7.16 (dd, J = 1.69 Hz; 10.39 Hz, 1H, H-5 indazole), 5.97 (d, J = 3.58 Hz; 1H, H-1′), 5.58 (m, 1H, OH-2′),

5.19 (m, 1H, OH-3′), 4.99 (t, J = 5.44 Hz; 10.84 Hz, 1H, OH-5′), 4.39 (m, 1H, H-2′), 4.19 (m, 1H, H-3′), 4.02 (q, J = 4.64 Hz; 13.90 Hz, 1H, H-4′), 3.71 (m, 1H, H_a-5′), 3.58 (m, 1H, H_b-5′).

^{13}C NMR: δ = 148.43 (C8 indazole), 124.39 (C6 indazole), 123.79 (C4 indazole), 122.95 (C3 indazole), 119.42 (C9 indazole), 119.15 (C7 indazole), 119.15 (C5 indazole), 94.67 (C1′), 85.14 (C4′), 75.27 (C2′), 69.82 (C3′), 61.07 (C5′).

^{15}N NMR: δ = 239.00 (N2).

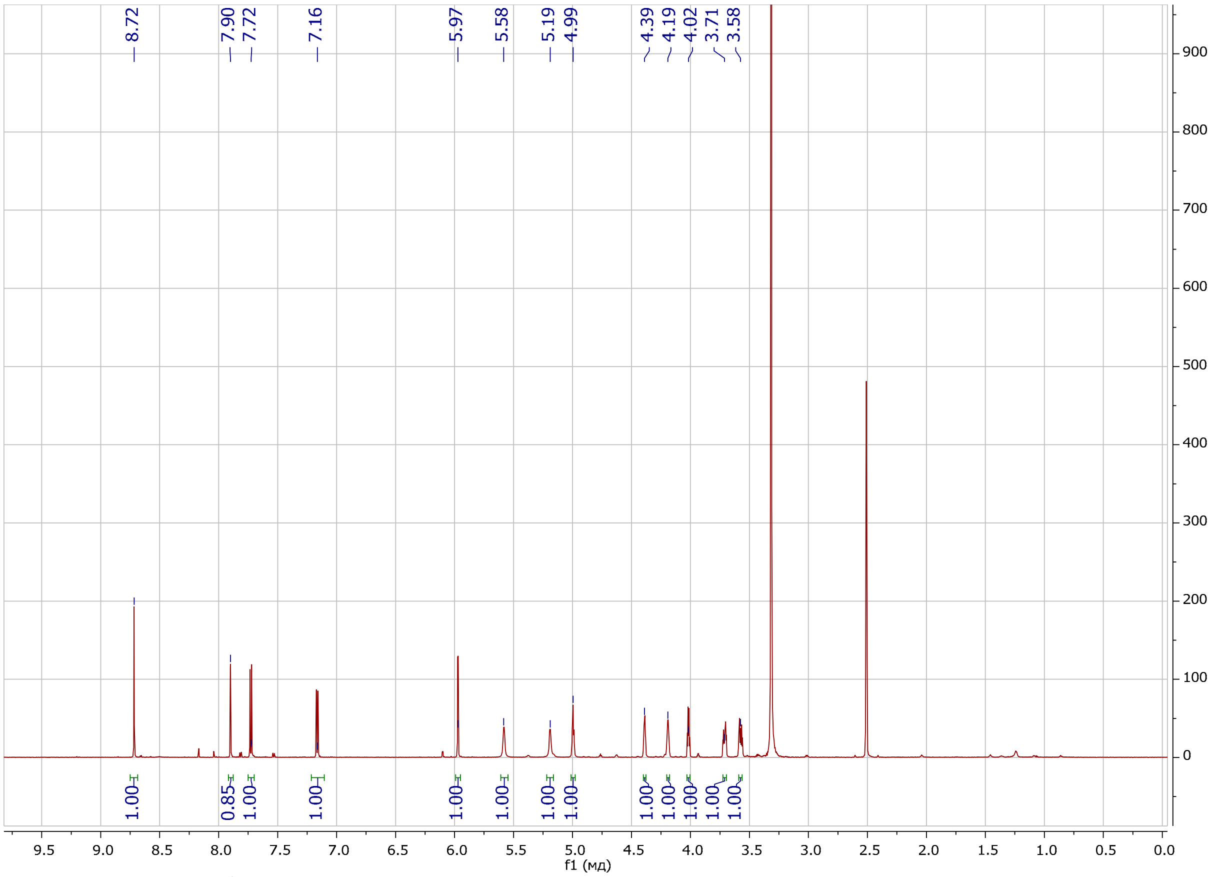

Figure S25. The ^{1}H NMR spectrum of N2-(β-D-ribofuranosyl)-6-bromo-indazole (N2)-**10** (700 MHz, DMSO-d_6, 303 K).

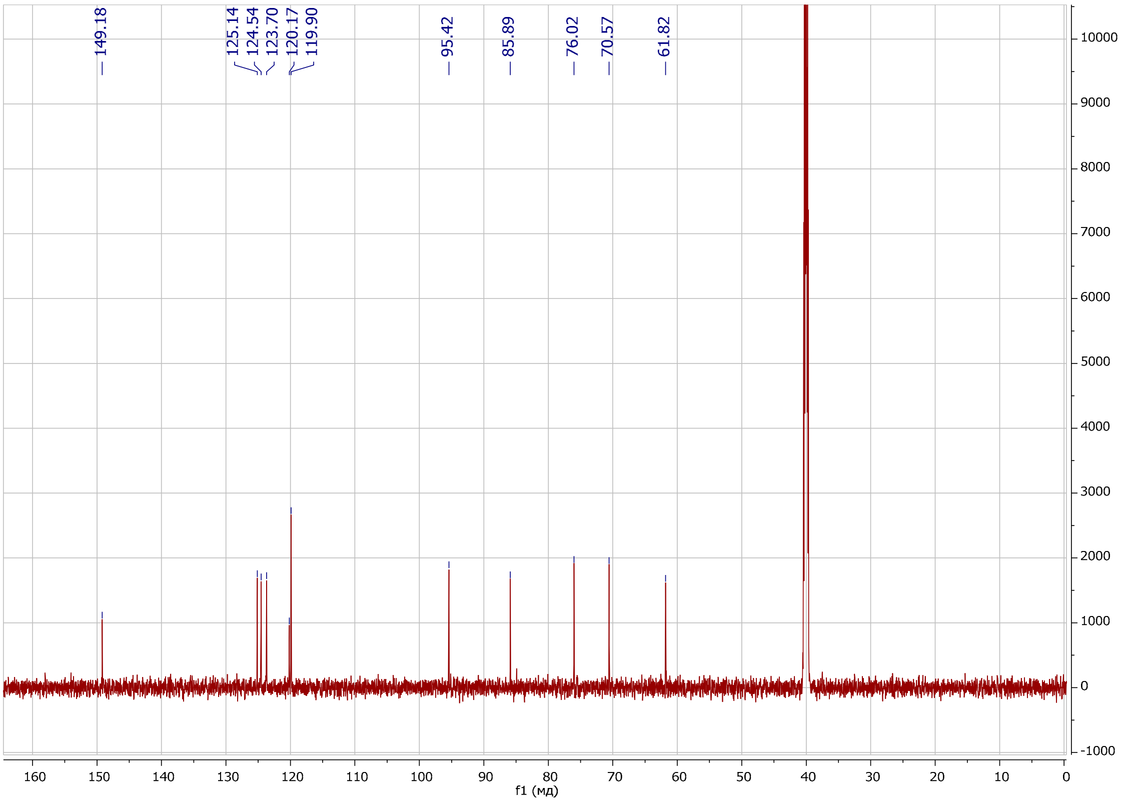

Figure S26. The ^{13}C NMR spectrum of N2-(β-D-ribofuranosyl)-6-bromo-indazole (N2)-**10** (176 MHz, DMSO-d_6, 303 K).

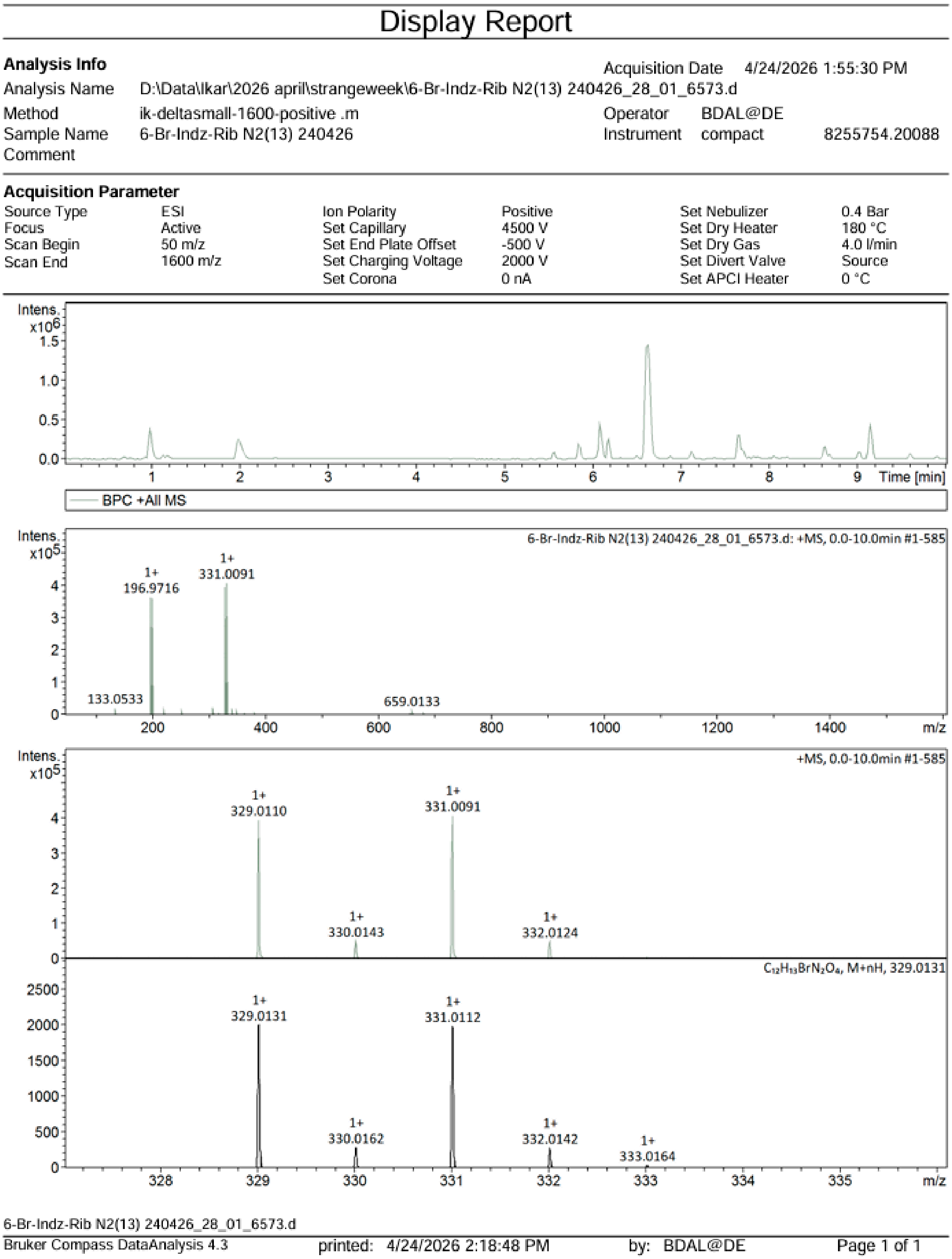

Figure S27. The HMRS data of (N2)-**10**.

IBCH RAS

Project Name Fleximer

Reported by User: Breeze user (Breeze)

Breeze 2 HPLC System

SAMPLE INFORMATION

Sample Name:	6-br-indz-rib prod 2 purity	Acquired By:	Breeze
Sample Type:	Unknown	Date Acquired:	19.08.2025 19:33:26 GMT-4
Vial:	1	Acq. Method:	100B_dual_280nm_05ml
Injection #:	1	Date Processed:	19.08.2025 19:57:35 GMT-4
Injection Volume:	10.00 ul	Channel Name:	W2489 ChB
Run Time:	20.00 Minutes	Channel Desc.:	W2489 ChB 280nm
Column Type:		Sample Set Name	b

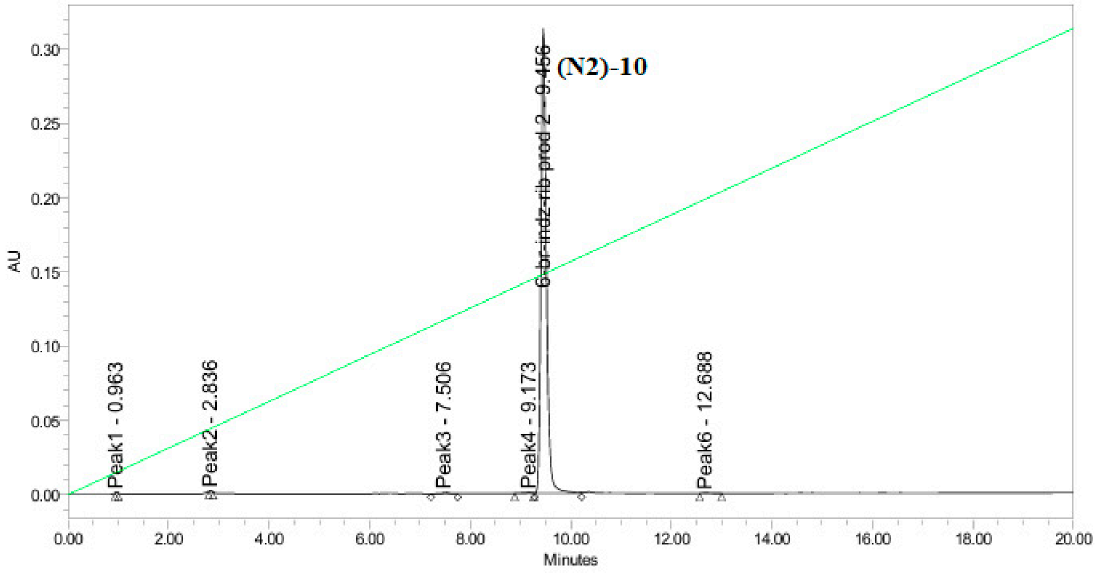

	Peak Name	RT (min)	Area (µV*sec)	% Area	Height (µV)	% Height
1	Peak1	0.963	681	0.03	270	0.09
2	Peak2	2.836	1987	0.08	627	0.20
3	Peak3	7.506	7391	0.31	813	0.26
4	Peak4	9.173	3706	0.16	441	0.14
5	6-br-indz-rib prod 2	9.456	2334135	99.34	313922	99.25
6	Peak6	12.688	1670	0.07	211	0.07

Report Method: Gradient Overlay Report ASC

Page: 1 of 1

Printed: 19.08.2025 19:58:16 Etc/GMT-4

Figure S28. The HPLS data of (N2)-**10**.

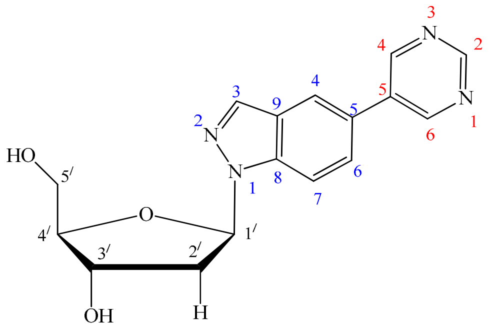

(N1)-**11**

N1-(β-D-2'deoxyribofuranosyl)-5-(pyrimidin-5-yl)-indazole ((N1)-11).

^{1}H NMR: δ = 9.20, (s, 2H, H-4, H-6 pyrimidine), 9.18 (s, 1H, H-2 pyrimidine), 8.26 (s, 1H, H-4 indazole), 8.24 (s., 1H, H-3 indazole), 7.98 (d., J = 8.87 Hz, 1H, H-7 indazole), 7.87 (dd, J = 1.45 Hz; 8.87 Hz, 1H, H-6 indazole), 6.66 (t, J = 6.13 Hz; 12.61 Hz, 1H, H-1′), 5.29 (d, J = 4.79 Hz 1H, OH-3′), 4.72 (t, J = 5.77 Hz; 11.37 Hz, 1H, H-5′), 4.49 (m, 1H, H-3′), 3.86 (m, 1H, H-4′) 3.51 (m, 1H, Ha-5′), 3.34 (m, 1H, Hb-5′), 2.95 (m, 1H, Ha-2′),2.32 (m, 1H, Hb-2′).

^{13}C NMR: δ = 155.66 (C2 pyrimidine), 154.58 (C4, C6 pyrimidine), 139.52 (C8 indazole), 134.63 (C4 indazole), 133.40 (C5 pyrimidine), 126.83 (C9 indazole), 125.54 (C6 indazole), 124.73 (C5 indazole), 119.49 (C3 indazole), 110.98 (C7 indazole), 87.41 (C4′), 85.70 (C1′), 70.91 (C3′), 62.23 (C5′), 38.17 (C2′).

^{15}N NMR: δ = 316.09 (N2 indazole), 295.09 (N1, N3 pyrimidine), 198.90 (N1 indazole).

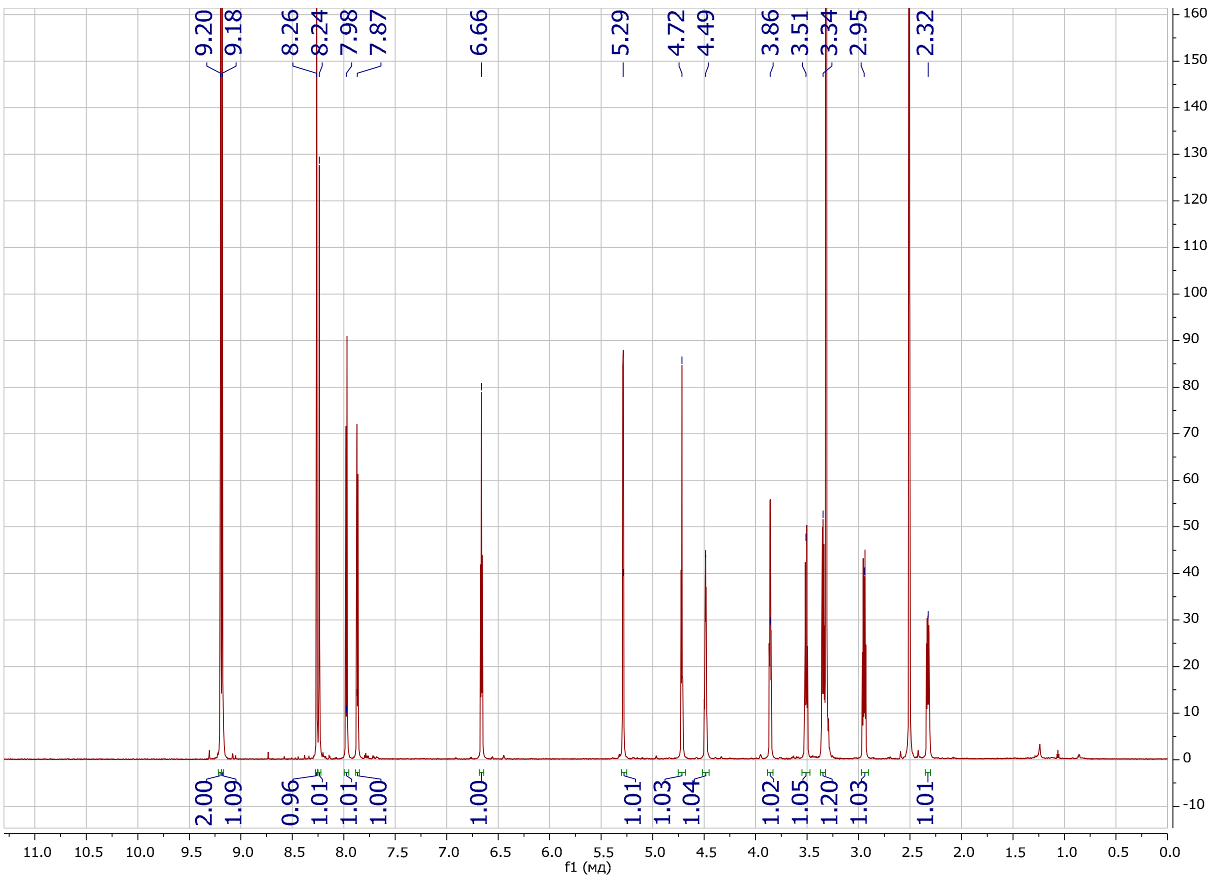

Figure S29. The ^{1}H NMR spectrum of N1-(β-D-2'deoxyribofuranosyl)-5-(pyrimidin-5-yl)-indazole (**N**1)-**11** (700 MHz, DMSO-d_6, 303 K).

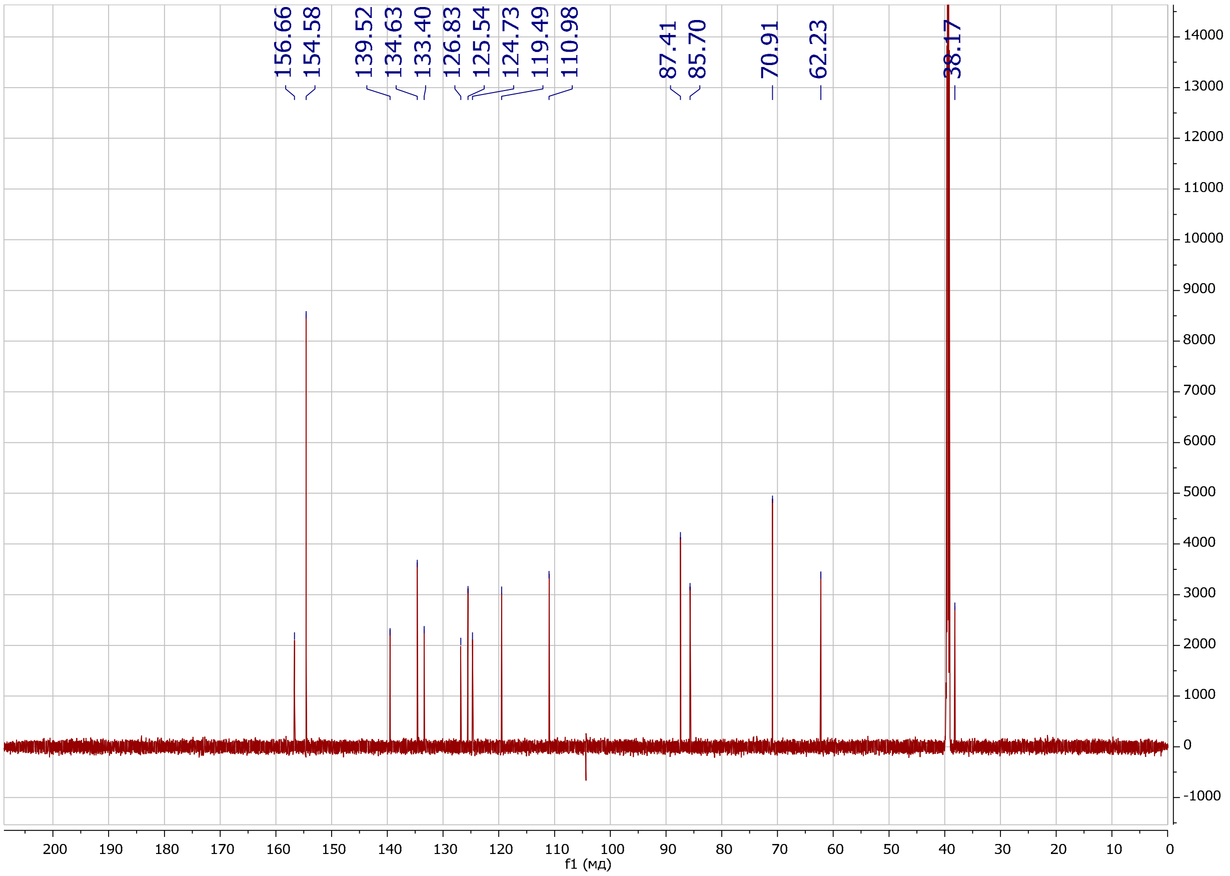

Figure S30. The ^{13}C NMR spectrum of N1-(β-D-2'deoxyribofuranosyl)-5-(pyrimidin-5-yl)-indazole (N1)-**11** (176 MHz, DMSO-d_6, 303 K).

Display Report

Analysis Info

Analysis Name	D:\Data\Ikar\2026 april\strangeweek\LN 270 2et drib(6) 240426_31_01_6576.d	Acquisition Date	4/24/2026 2:45:20 PM
Method	ik-deltasmall-1600-positive .m	Operator	BDAL@DE
Sample Name	LN 270 2et drib(6) 240426	Instrument	compact 8255754.20088
Comment			

Acquisition Parameter

Source Type	ESI	Ion Polarity	Positive	Set Nebulizer	0.4 Bar
Focus	Active	Set Capillary	4500 V	Set Dry Heater	180 °C
Scan Begin	50 m/z	Set End Plate Offset	-500 V	Set Dry Gas	4.0 l/min
Scan End	1600 m/z	Set Charging Voltage	2000 V	Set Divert Valve	Source
		Set Corona	0 nA	Set APCI Heater	0 °C

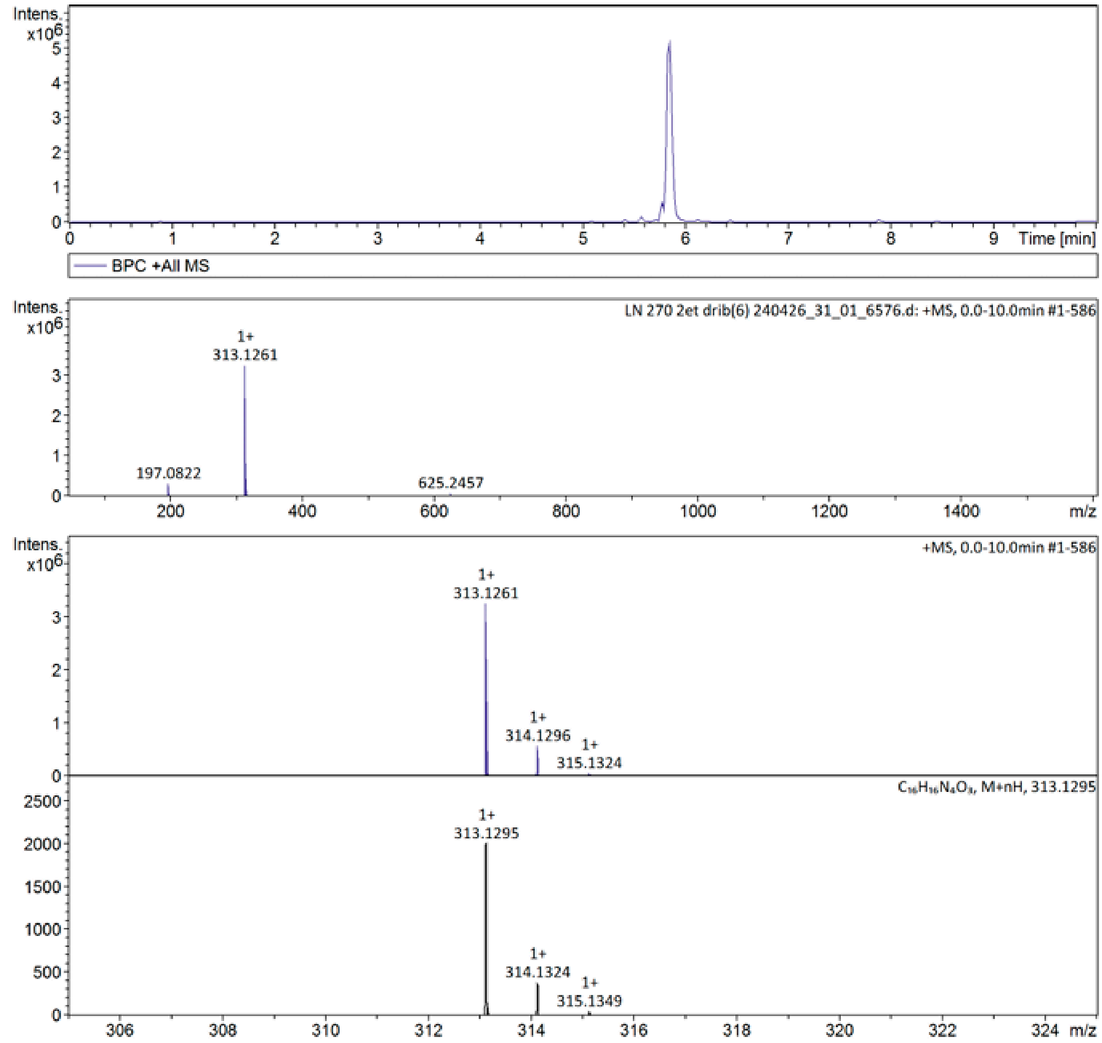

LN 270 2et drib(6) 240426_31_01_6576.d

Bruker Compass DataAnalysis 4.3 printed: 4/24/2026 3:26:21 PM by: BDAL@DE Page 1 of 1

Figure S31. The HMRS data of (N1)-**11.**

IBCH RAS

Project Name Defaults

Reported by User: Breeze user (Breeze)

Breeze 2 HPLC System

SAMPLE INFORMATION

Sample Name:	LN-270-2dRib purity	Acquired By:	Breeze
Sample Type:	Unknown	Date Acquired:	07.11.2023 13:42:05 GMT-4
Vial:	16	Acq. Method:	100B_dual_280nm_05ml
Injection #:	1	Date Processed:	10.11.2025 13:53:10 GMT-4
Injection Volume:	5.00 ul	Channel Name:	W2489 ChB
Run Time:	30.00 Minutes	Channel Desc.:	W2489 ChB 280nm
Column Type:		Sample Set Name	

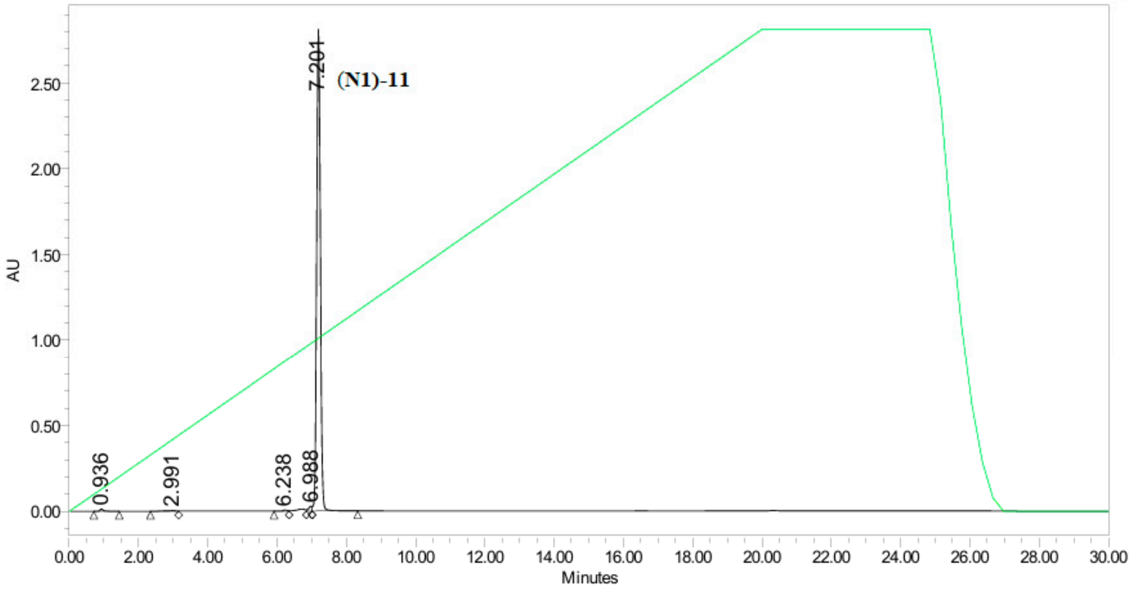

	RT (min)	Area (µV*sec)	% Area	Height (µV)	% Height
1	0.936	78402	0.37	12935	0.45
2	2.991	60886	0.29	4325	0.15
3	6.238	50012	0.23	6353	0.22
4	6.988	182132	0.85	28253	0.99
5	7.201	20979023	98.26	2809228	98.19

Report Method: Gradient Overlay Report ASC

Page: 1 of 1

Printed: 10.11.2025 13:53:35 Etc/GMT-4

Figure S32. The HPLS data of (N1)-**11.**

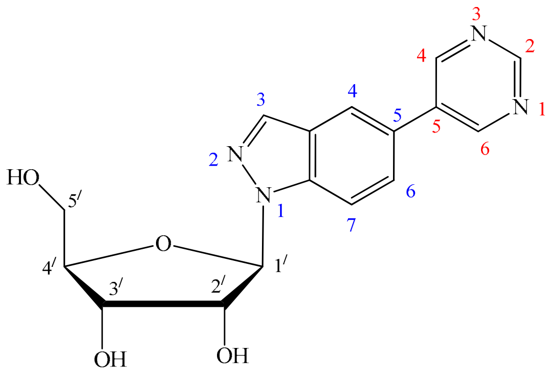

(N1)-**12**

N1-(β-D-ribofuranosyl)-5-(pyrimidin-5-yl)-indazole ((N1)-12).

^{1}H NMR: δ = 9.20, (s, 2H, H-4, H-6 pyrimidine), 9.18 (s, 1H, H-2 pyrimidine),8.29, (s, 1H, H-4 indazole), 8.25 (s, 1H, H-3 indazole), 7.99 (d., J = 8.82 Hz, 1H, H-7 indazole), 7.87 (dd, J = 1.72 Hz; 10.41 Hz, 1H, H-6 indazole), 6.18 (d, J = 4.54 Hz; 1H, H-1′), 5.37 (d, J = 5.88 Hz 1H, OH-2′), 5.15 (d, J = 5.20 Hz, 1H, OH-3′), 4.78 (t, J = 5.74 Hz; 11.33 Hz, 1H, OH-5′), 4.67 (q, J = 5.24 Hz; 15.09 Hz, 1H, H-2′), 4.25 (q, J = 5.05 Hz; 14.26 Hz, 1H, H-3′), 3.96 (q, J = 4.61 Hz; 14.50 Hz, 1H, H-4′), 3.60 (m, 1H, Ha-5′), 3.45 (m, 1H, Hb-5′).

^{13}C NMR: δ = 156.80 (C2 pyrimidine), 154.71 (C4, C6 pyrimidine), 139.89 (C8 indazole), 135.07 (C4 indazole), 133.51 (C5 pyrimidine), 127.00 (C9 indazole), 125.77 (C6 indazole), 124.80 (C5 indazole), 119.66 (C3 indazole), 111.05 (C7 indazole), 90.35 (C1′), 85.00 (C4′), 73.36 (C2′), 70.74 (C3′), 62.24 (C5′).

^{15}N NMR: δ = 317.86 (N2 indazole), 295.03 (N1, N3 pyrimidine), 195.24 (N1 indazole).

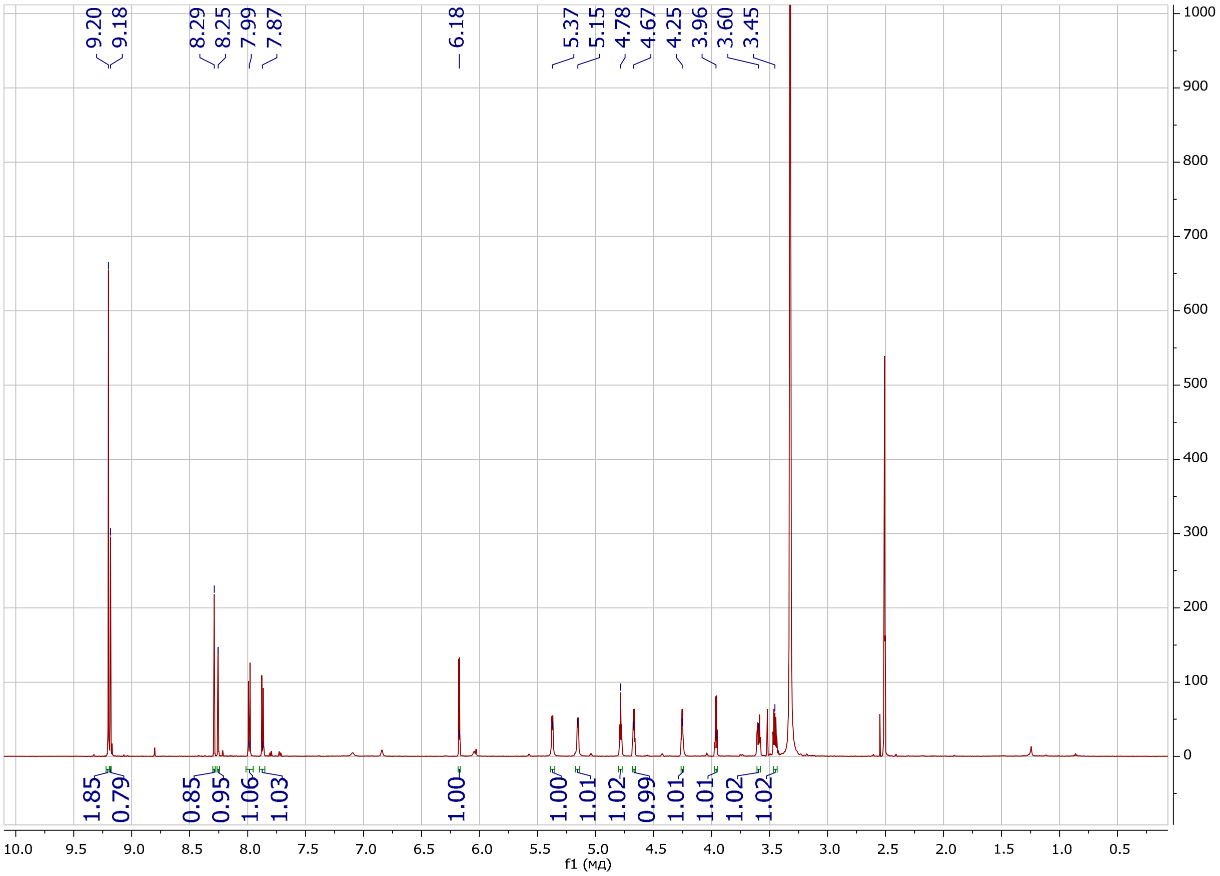

Figure S33. The ^{1}H NMR spectrum of N1-(β-D-ribofuranosyl)-5-(pyrimidin-5-yl)-indazole (**N**1)-**12** with minor N2 product (700 MHz, DMSO-d_6, 303 K).

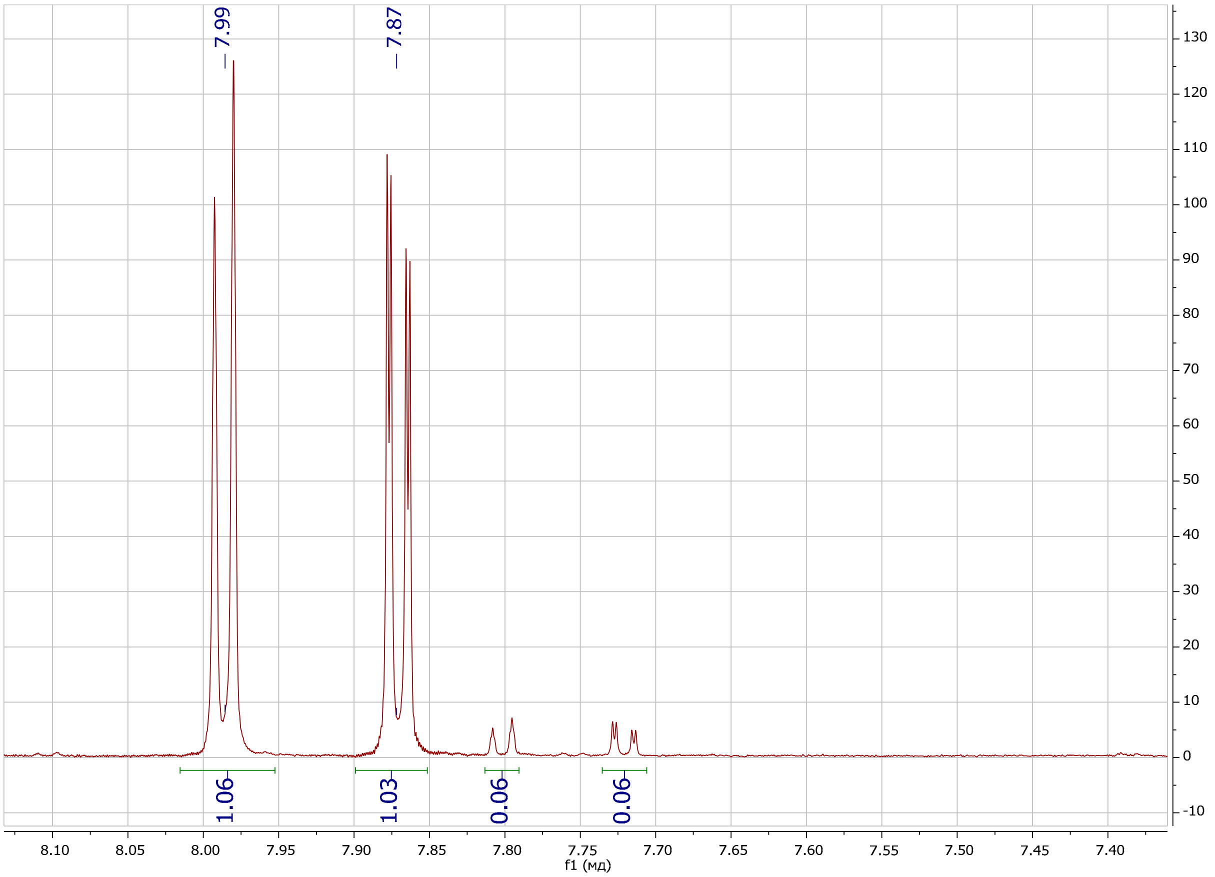

Figure S34. The fragment of ^{1}H NMR spectrum of (N1)-**12** with minor N2 product (700 MHz, DMSO-d_6, 303 K).

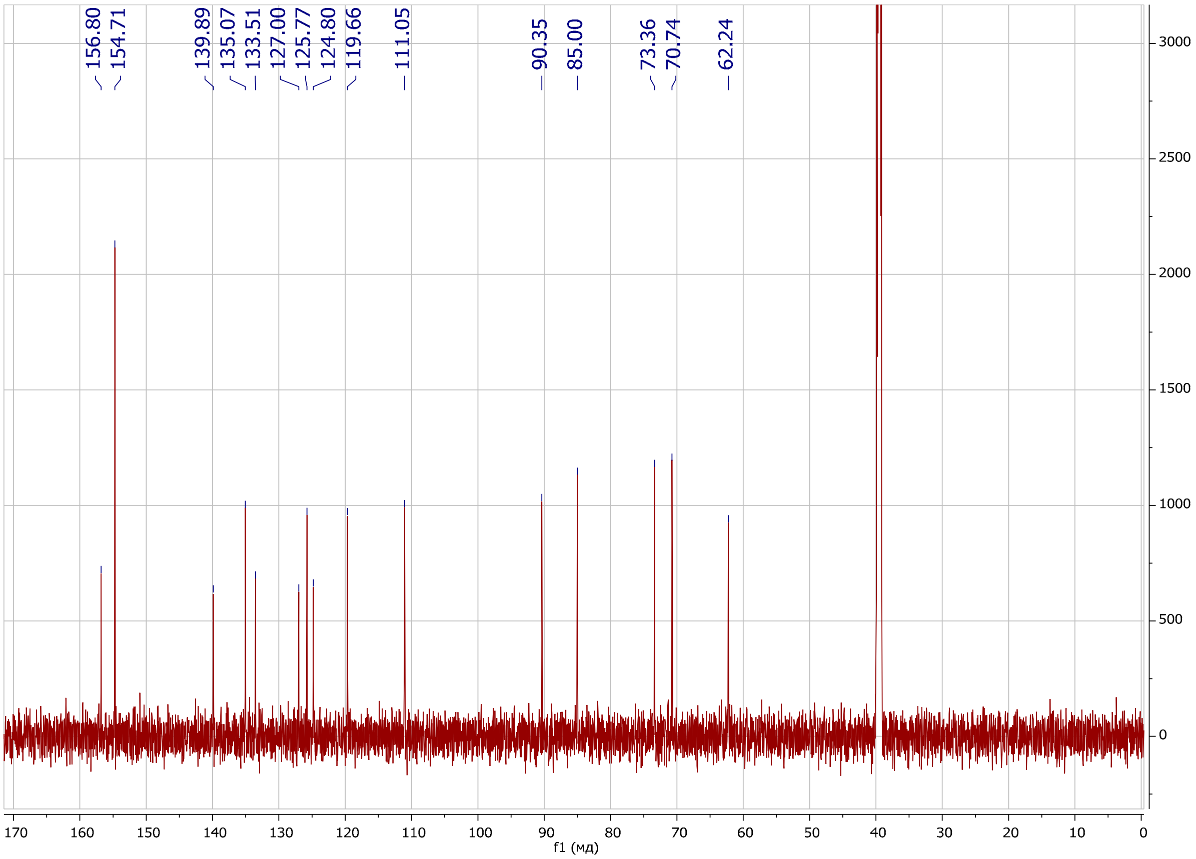

Figure S35. The ^{13}C NMR spectrum of of N1-(β-D-ribofuranosyl)-5-(pyrimidin-5-yl)-indazole (N1)-**12** (176 MHz, DMSO-d_6, 303 K).

Display Report

Analysis Info

Analysis Name	D:\Data\Ikar\2026 april\winterweek\LN 270 Rib(5)-2 270426_23_01_6591.d	Acquisition Date	4/27/2026 4:30:18 PM
Method	ik-deltasmall-1600-positive .m	Operator	BDAL@DE
Sample Name	LN 270 Rib(5)-2 270426	Instrument	compact 8255754.20088
Comment			

Acquisition Parameter

Source Type	ESI	Ion Polarity	Positive	Set Nebulizer	0.4 Bar
Focus	Active	Set Capillary	4500 V	Set Dry Heater	180 °C
Scan Begin	50 m/z	Set End Plate Offset	-500 V	Set Dry Gas	4.0 l/min
Scan End	1600 m/z	Set Charging Voltage	2000 V	Set Divert Valve	Source
		Set Corona	0 nA	Set APCI Heater	0 °C

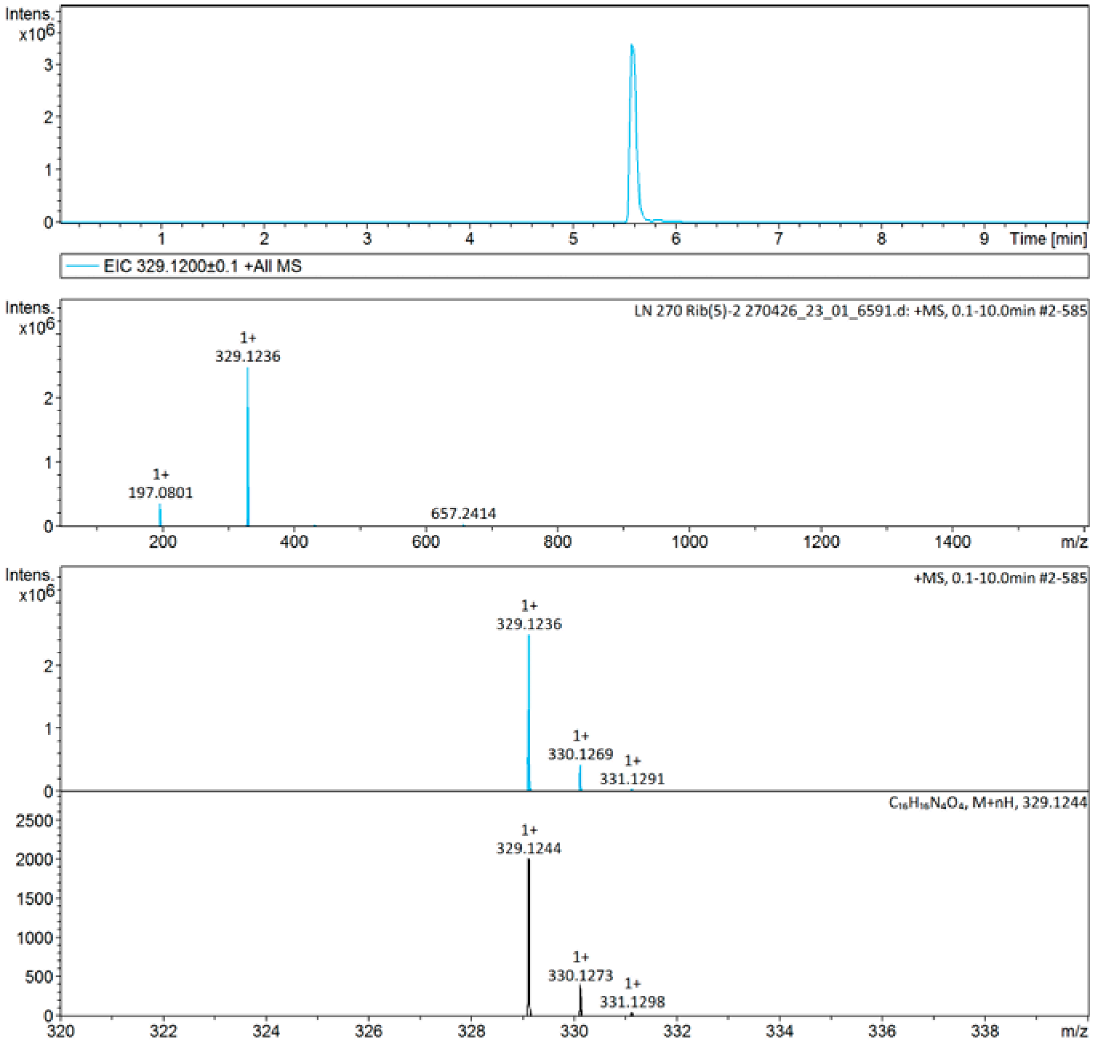

LN 270 Rib(5)-2 270426_23_01_6591.d

Bruker Compass DataAnalysis 4.3 printed: 4/27/2026 5:21:07 PM by: BDAL@DE Page 1 of 1

Figure S36. The HMRS data of (N1)-**12.**

IBCH RAS

Project Name Fleximer

Reported by User: Breeze user (Breeze)

SAMPLE INFORMATION

Sample Name:	LN-270-rib purity	Acquired By:	Breeze
Sample Type:	Unknown	Date Acquired:	02.06.2025 15:24:16 GMT-4
Vial:	1	Acq. Method:	100B_dual_280nm_05ml
Injection #:	1	Date Processed:	10.11.2025 13:40:15 GMT-4
Injection Volume:	10.00 ul	Channel Name:	W2489 ChA
Run Time:	20.00 Minutes	Channel Desc.:	W2489 ChA 254nm
Column Type:		Sample Set Name	g

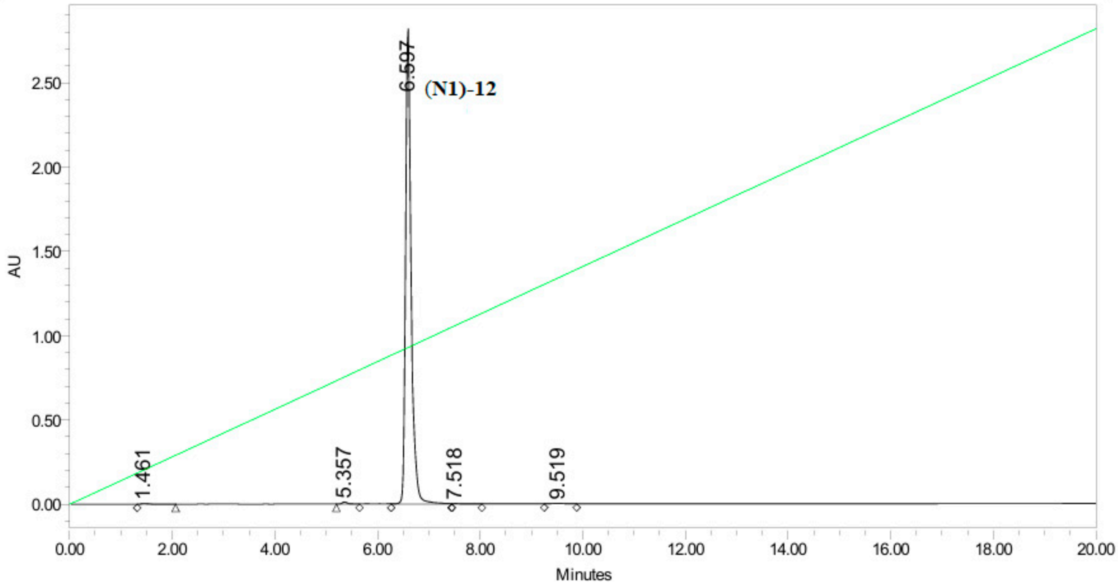

	RT (min)	Area (µV*sec)	% Area	Height (µV)	% Height
1	1.461	33394	0.15	3888	0.14
2	5.357	83248	0.39	11803	0.42
3	6.597	21365619	99.02	2814970	99.24
4	7.518	48462	0.22	2864	0.10
5	9.519	47171	0.22	3012	0.11

Report Method: Gradient Overlay Report ASC

Printed: 10.11.2025 13:41:11 Etc/GMT-4

Page: 1 of 1

Figure S37. The HPLC data of (N1)-**12.**

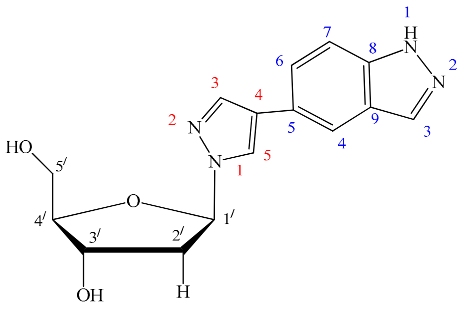

13

N1-(β-D-2'deoxyribofuranosyl)-5-(pyrazol-4-yl)-1H-indazole (13)

^{1}H NMR: δ =12.98, (s, 1H-weak, N1-H-1 indazole), 8.35 (s, 1H, H-5 pyrazole), 8.05 (s, 1H, H-3 indazole), 7.97 (s., 1H, H-3 pyrazole), 7.95 (s., 1H, H-4 indazole), 7.60 (dd, J = 1.53 Hz; 8.36 Hz, 1H, H-6 indazole), 7.54 (d, J = 8.59 Hz 1H, H-7 indazole), 6.12 (t, J = 6.17 Hz; 13.01 Hz, 1H, H-1′), 5.25 (s, 1H, OH-3′), 4.85 (s, 1H, OH-5′), 4.39 (m, 1H, H-3′), 3.85 (q, J = 5.09 Hz; 8.30 Hz, 1H, H-4′), 3.55 (m, 1H, Ha-5′), 3.47 (m, 1H, Hb-5′), 2.65 (m, 1H, Ha-2′), 2.27 (m, 1H, Hb-2′).

^{13}C NMR: δ = 139.40 (C9 indazole), 137.32 (C3 pyrazole), 133.76 (C3 indazole), 126.44 (C5 pyrazole), 125.18 (C6 indazole), 125.11 (C8 indazole), 123.84 (C5 indazole), 123.36 (C4 pyrazole), 116.44 (C4 indazole), 111.04 (C7 indazole), 89.68 (C1′), 88.25 (C4′), 71.35 (C3′), 62.68 (C5′), 40.34 (C2′).

^{15}N NMR: δ = 315.66 (N2 indazole), 303.83 (N2 pyrazole), 225.05 (N1 pyrazole), 185.35 (N1 indazole).

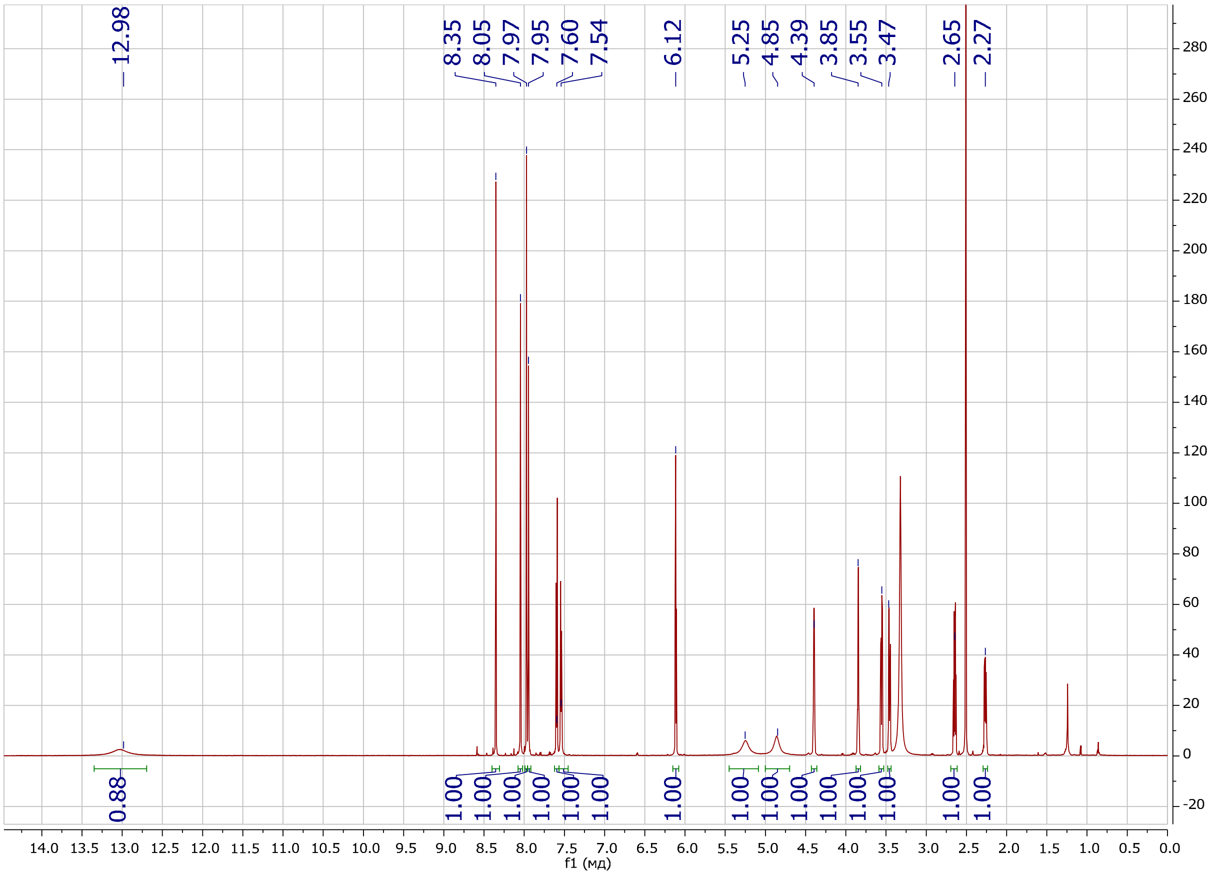

Figure S38. The ^{1}H NMR spectrum of N1-(β-D-2'deoxyribofuranosyl)-5-(pyrazol-4-yl)-1H-indazole (**13**) (700 MHz, DMSO-d_6, 303 K).

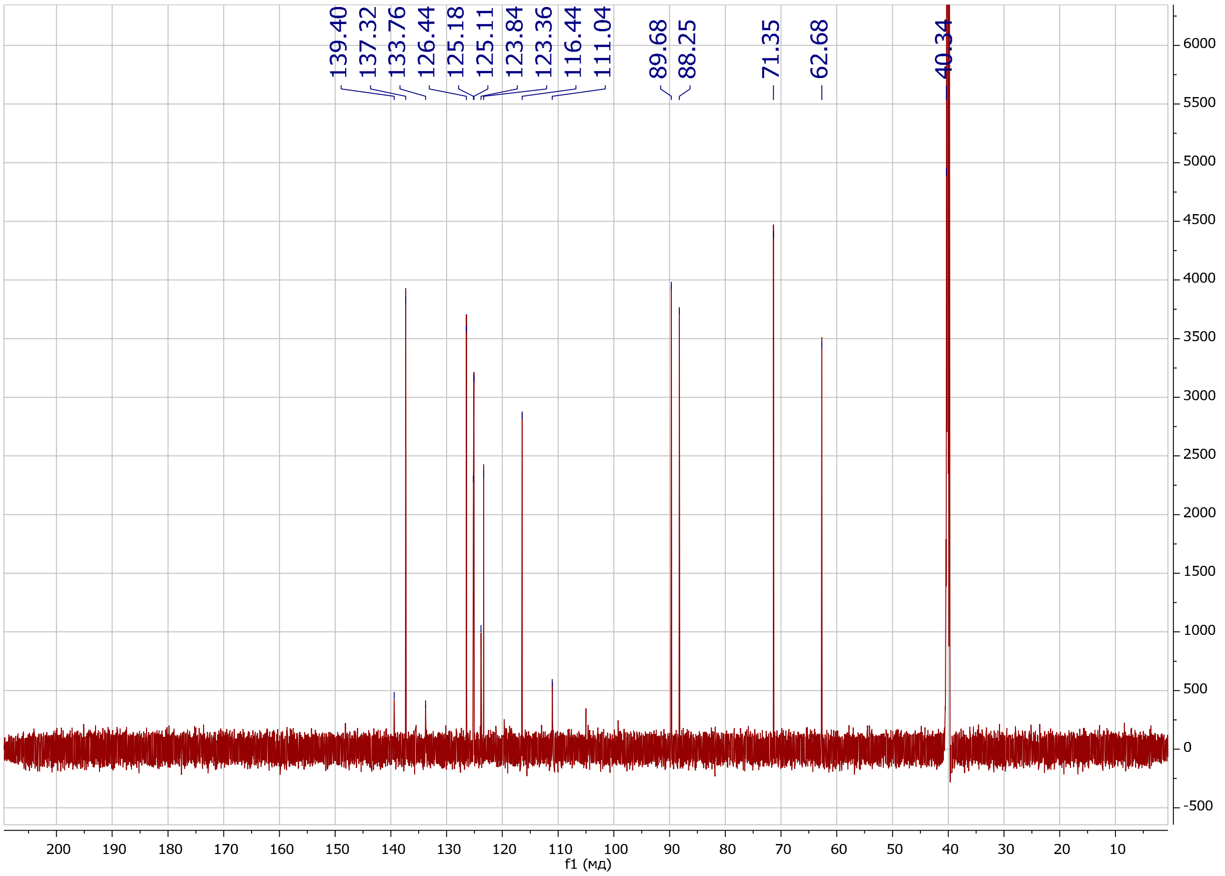

Figure S39. The ^{13}C NMR spectrum of N1-(β-D-2'deoxyribofuranosyl)-5-(pyrazol-4-yl)-1H-indazole (**13**) (176 MHz, DMSO-d_6, 303 K).

Display Report

Analysis Info

Analysis Name	D:\Data\Ikar\2026 april\winterweek\LN 295-2etdRib(4)-2 270426_24_01_6592.d	Acquisition Date	4/27/2026 4:46:46 PM
Method	ik-deltasmall-1600-positive .m	Operator	BDAL@DE
Sample Name	LN 295-2etdRib(4)-2 270426	Instrument	compact 8255754.20088
Comment			

Acquisition Parameter

Source Type	ESI	Ion Polarity	Positive	Set Nebulizer	0.4 Bar
Focus	Active	Set Capillary	4500 V	Set Dry Heater	180 °C
Scan Begin	50 m/z	Set End Plate Offset	-500 V	Set Dry Gas	4.0 l/min
Scan End	1600 m/z	Set Charging Voltage	2000 V	Set Divert Valve	Source
		Set Corona	0 nA	Set APCI Heater	0 °C

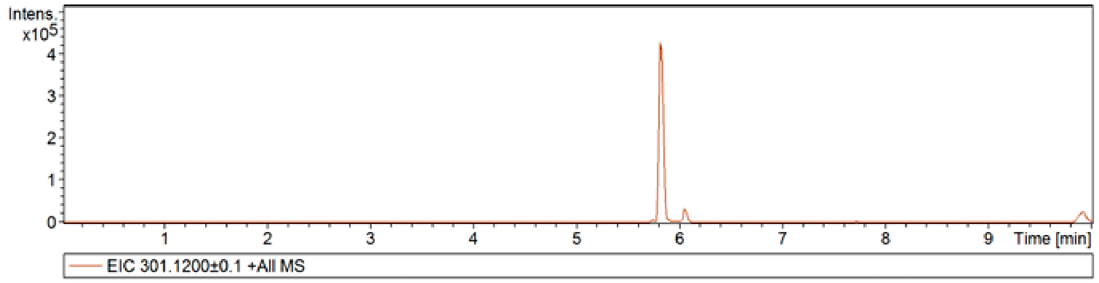

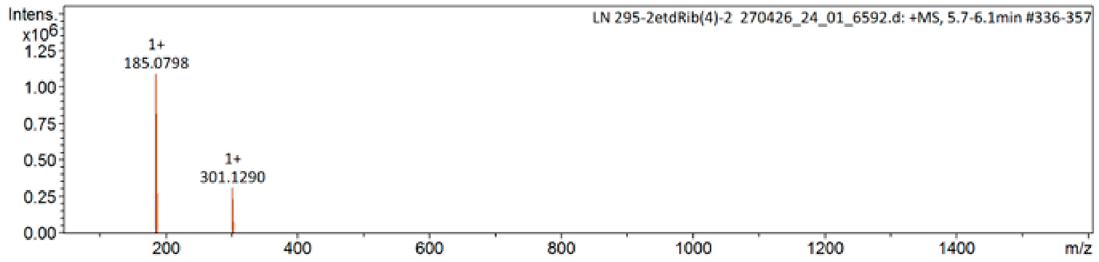

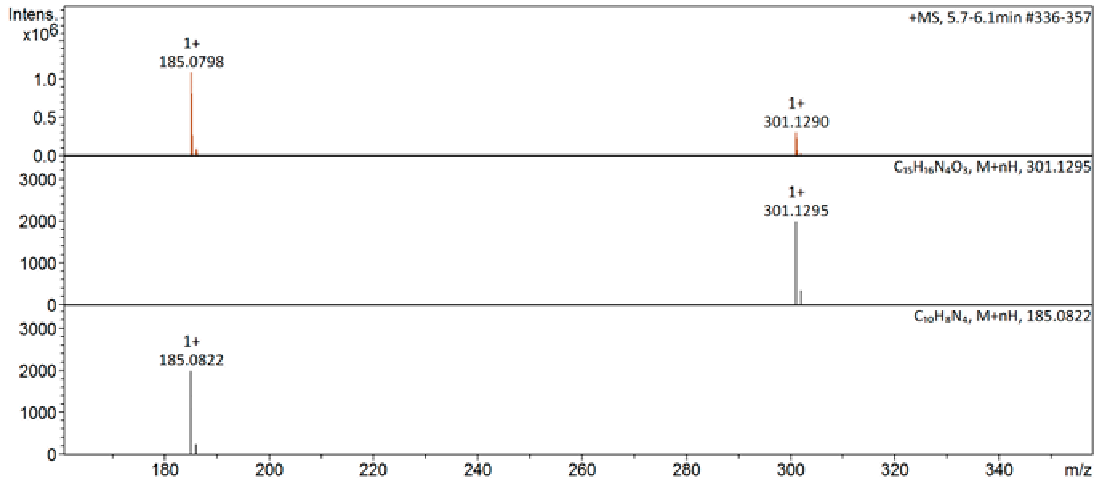

LN 295-2etdRib(4)-2 270426_24_01_6592.d

Bruker Compass DataAnalysis 4.3 printed: 4/27/2026 5:17:44 PM by: BDAL@DE Page 1 of 1

Figure S40. The HMRS data of **13**.

IBCH RAS

Project Name Defaults

Reported by User: Breeze user (Breeze)

SAMPLE INFORMATION

Sample Name:	LN-295-2dRib purity	Acquired By:	Breeze
Sample Type:	Unknown	Date Acquired:	07.11.2023 14:23:31 GMT-4
Vial:	17	Acq. Method:	100B_dual_280nm_05ml
Injection #:	1	Date Processed:	10.11.2025 14:15:23 GMT-4
Injection Volume:	10.00 ul	Channel Name:	W2489 ChA
Run Time:	30.00 Minutes	Channel Desc.:	W2489 ChA 254nm
Column Type:		Sample Set Name	

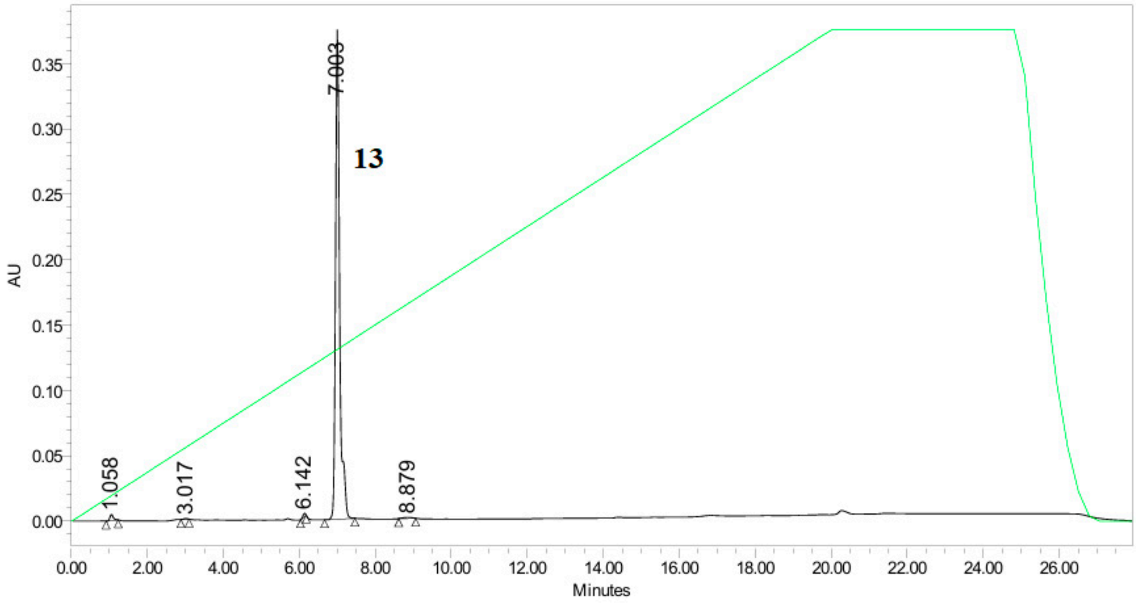

	RT (min)	Area (µV*sec)	% Area	Height (µV)	% Height
1	1.058	29530	1.01	4795	1.26
2	3.017	4025	0.14	569	0.15
3	6.142	11324	0.39	2443	0.64
4	7.003	2858448	98.01	373441	97.74
5	8.879	13299	0.46	817	0.21

Report Method: Gradient Overlay Report ASC

Page: 1 of 1

Printed: 10.11.2025 14:15:52 Etc/GMT-4

Figure S41. The HPLS data of **13.**

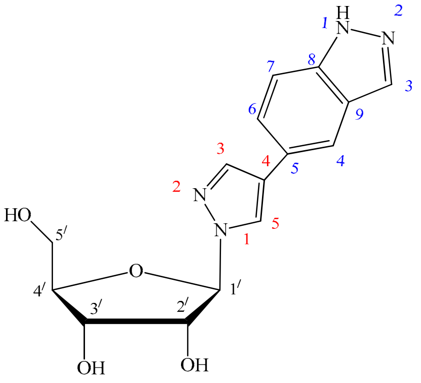

14

N1-(β-D-ribofuranosyl)-5-(pyrazol-4-yl)-1H-indazole (14)

^{1}H NMR: δ =13.03, (s, 1H, N1-H-1 indazole), 8.40 (s, 1H, H-5 pyrazole), 8.05 (s., 1H, H- H-3 indazole), 7.99 (s., 1H, H-3 pyrazole), 7.95 (s., 1H, H-4 indazole), 7.60 (d, J = 8.55 Hz, 1H, H-7 indazole), 7.55 (d, J = 8.75 Hz, 1H, H -6 indazole), 5.68 (d, J = 4.40 Hz, 1H, H-1′), 5.40 (d, J = 5.24 Hz, 1H, OH-2′), 5.11 (d, J = 4.20 Hz, 1H, OH-3′), 4.91 (t, J = 5.34 Hz; 10.80 Hz, 1H, OH-5′), 4.39 (m, 1H, H-2′), 4.16 (m, 1H, H-3′), 3.92 (q, J = 4.42 Hz; 9.25 Hz, 1H, H-4′), 3.64 (m, 1H, Ha-5′), 3.52 (m, 1H, Hb-5′).

^{13}C NMR: δ = 138.63 (C9 indazole), 136.81 (C3 pyrazole), 133.15 (C3 indazole), 125.95 (C8 indazole), 124.45 (C5 pyrazole), 124.39 (C6 indazole), 123.15 (C4 pyrazole), 122.65 (C4 indazole), 115.73 (C5 indazole), 110.27 (C7 indazole), 93.21 (C1′), 84.81 (C4′), 74.24 (C2′), 70.26 (C3′), 61.62 (C5′).

^{15}N NMR: δ = 315.68 (N2 indazole), 305.74 (N2 pyrazole), 221.44 (N1 pyrazole), 183.86 (N1 indazole).

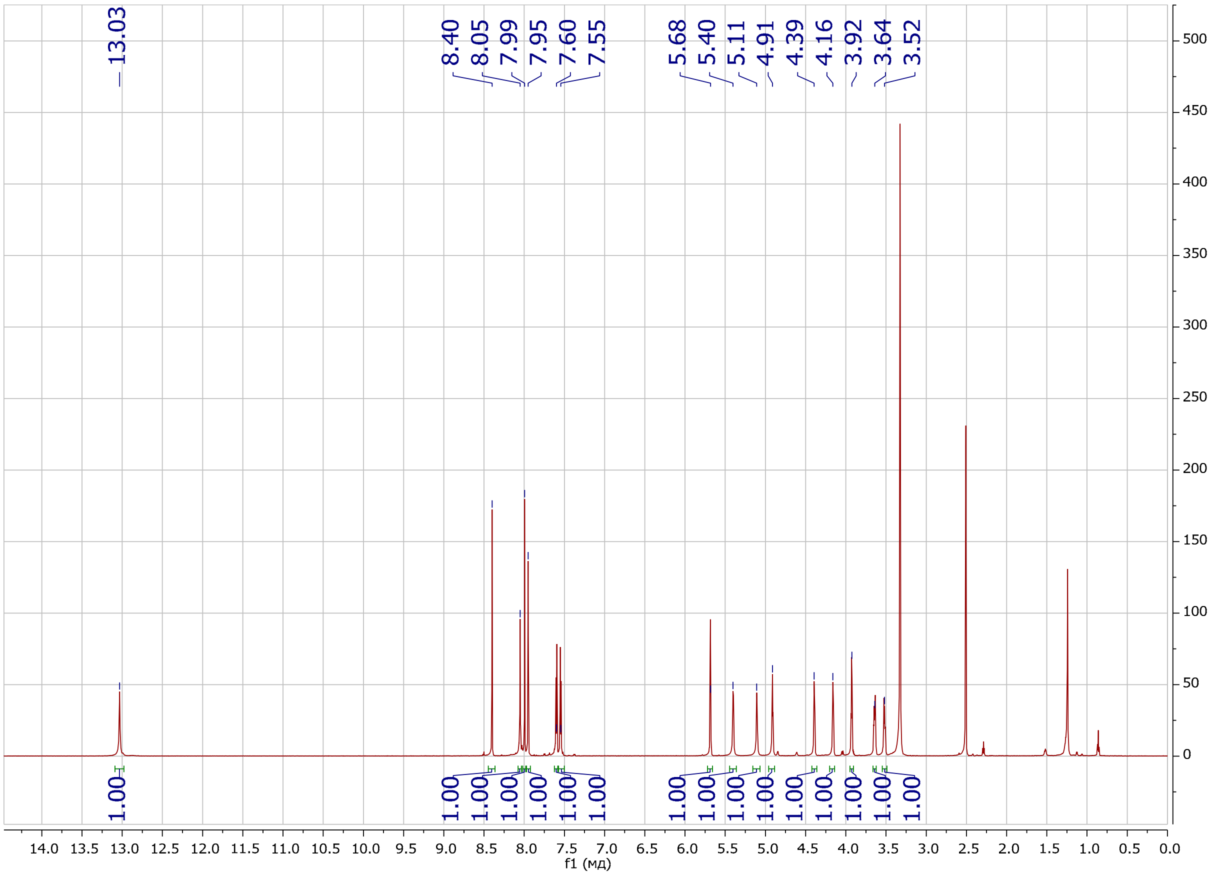

Figure S42. The ^{1}H NMR spectrum of N1-(β-D-ribofuranosyl)-5-(pyrazol-4-yl)-1H-indazole (**14**) (700 MHz, DMSO-d_6, 303 K).

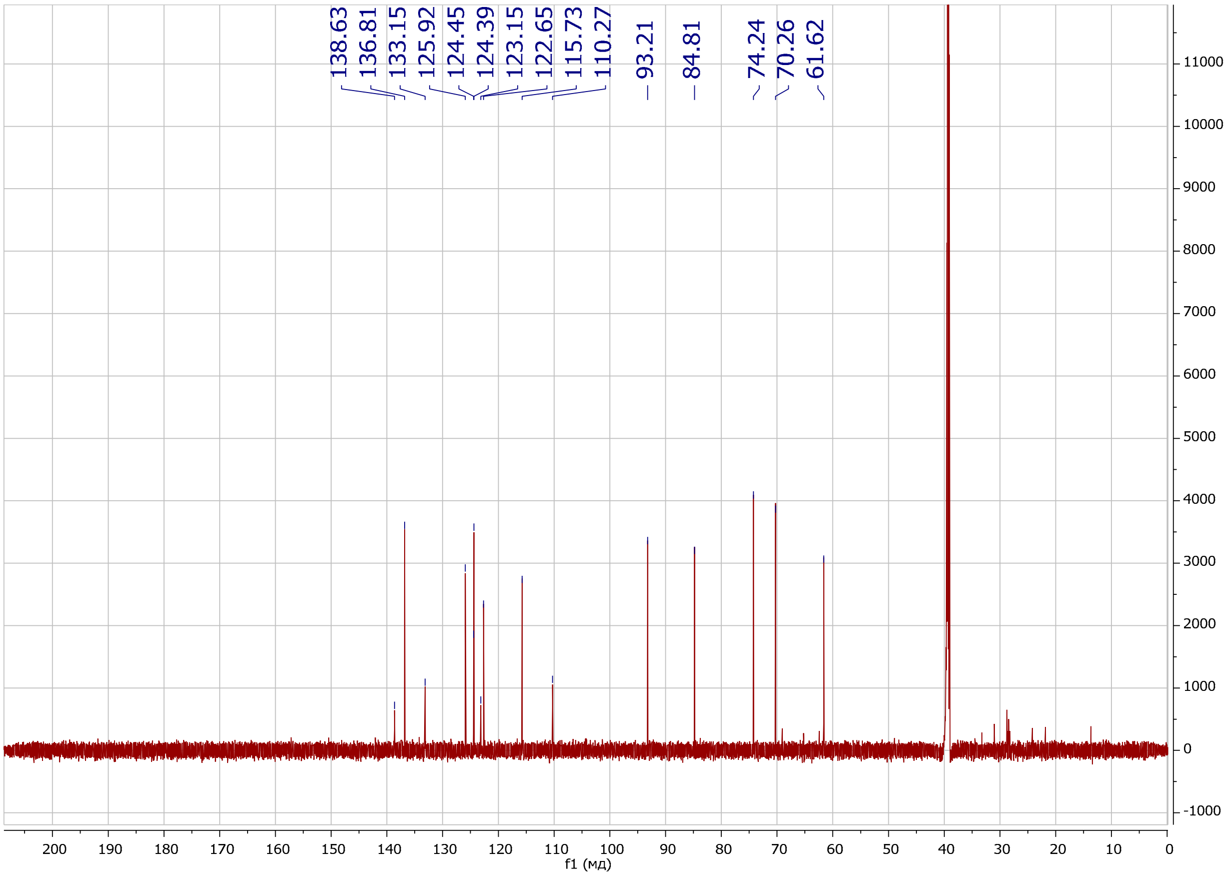

Figure S43. The ^{13}C NMR spectrum of N1-(β-D-ribofuranosyl)-5-(pyrazol-4-yl)-1H-indazole (**14**) (176 MHz, DMSO-d_6, 303 K).

Display Report

Analysis Info

Analysis Name	D:\Data\Ikar\2026 april\strangeweek\LN 295 rib(3) 220426 240426_23_01_6568.d	Acquisition Date	4/24/2026 12:32:54 PM
Method	ik-deltasmall-1600-positive .m	Operator	BDAL@DE
Sample Name	LN 295 rib(3) 220426 240426	Instrument	compact 8255754.20088
Comment			

Acquisition Parameter

Source Type	ESI	Ion Polarity	Positive	Set Nebulizer	0.4 Bar
Focus	Active	Set Capillary	4500 V	Set Dry Heater	180 °C
Scan Begin	50 m/z	Set End Plate Offset	-500 V	Set Dry Gas	4.0 l/min
Scan End	1600 m/z	Set Charging Voltage	2000 V	Set Divert Valve	Source
		Set Corona	0 nA	Set APCI Heater	0 °C

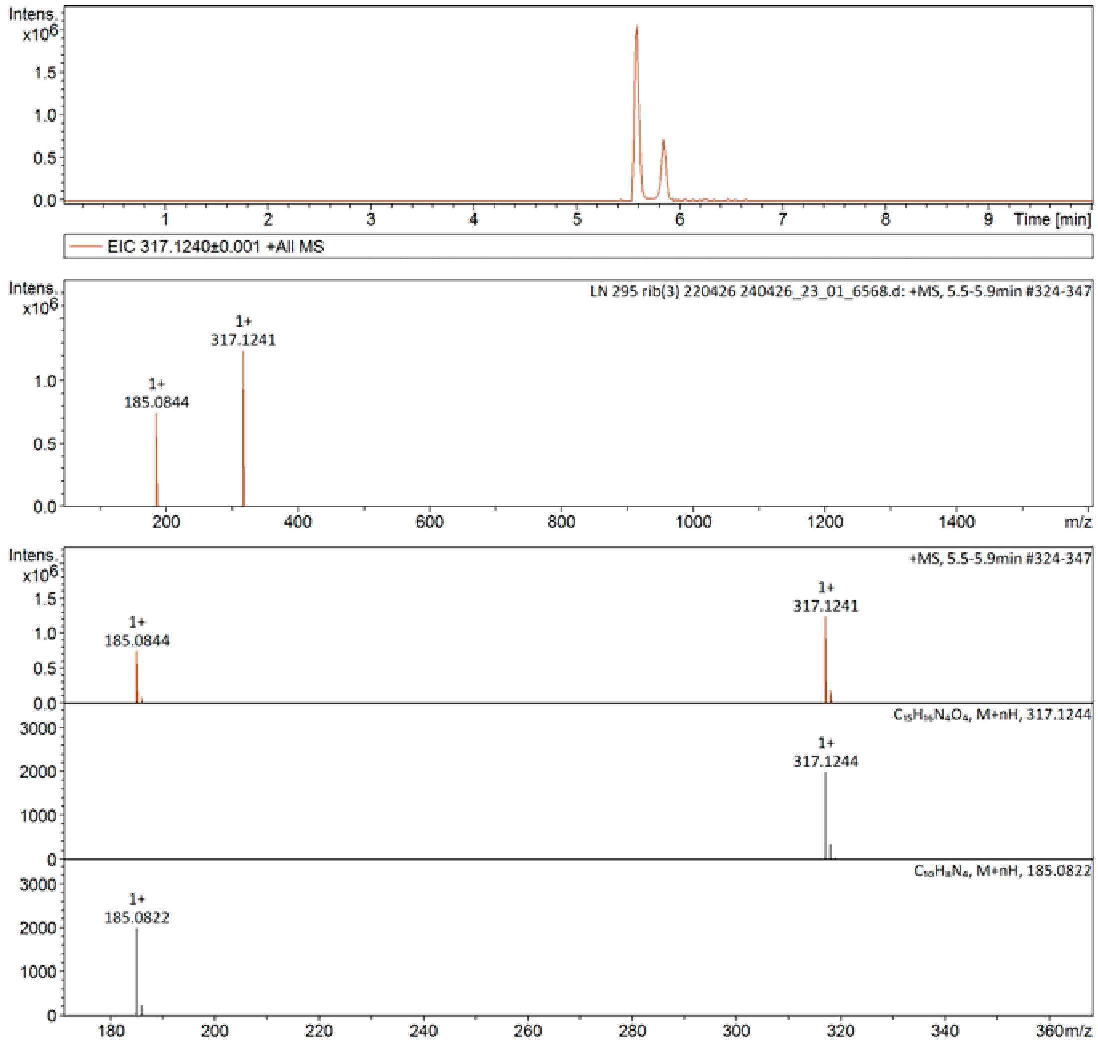

LN 295 rib(3) 220426 240426_23_01_6568.d

Bruker Compass DataAnalysis 4.3 printed: 4/24/2026 1:43:39 PM by: BDAL@DE Page 1 of 1

Figure S44. The HMRS data of **14**.

IBCH RAS

Project Name Defaults

Reported by User: Breeze user (Breeze)

SAMPLE INFORMATION

Sample Name:	LN-295-Rib purity	Acquired By:	Breeze
Sample Type:	Unknown	Date Acquired:	05.12.2023 20:14:55 GMT-4
Vial:	3	Acq. Method:	100B_dual_280nm_05ml
Injection #:	1	Date Processed:	10.11.2025 13:49:40 GMT-4
Injection Volume:	10.00 ul	Channel Name:	W2489 ChB
Run Time:	28.00 Minutes	Channel Desc.:	W2489 ChB 280nm
Column Type:		Sample Set Name	

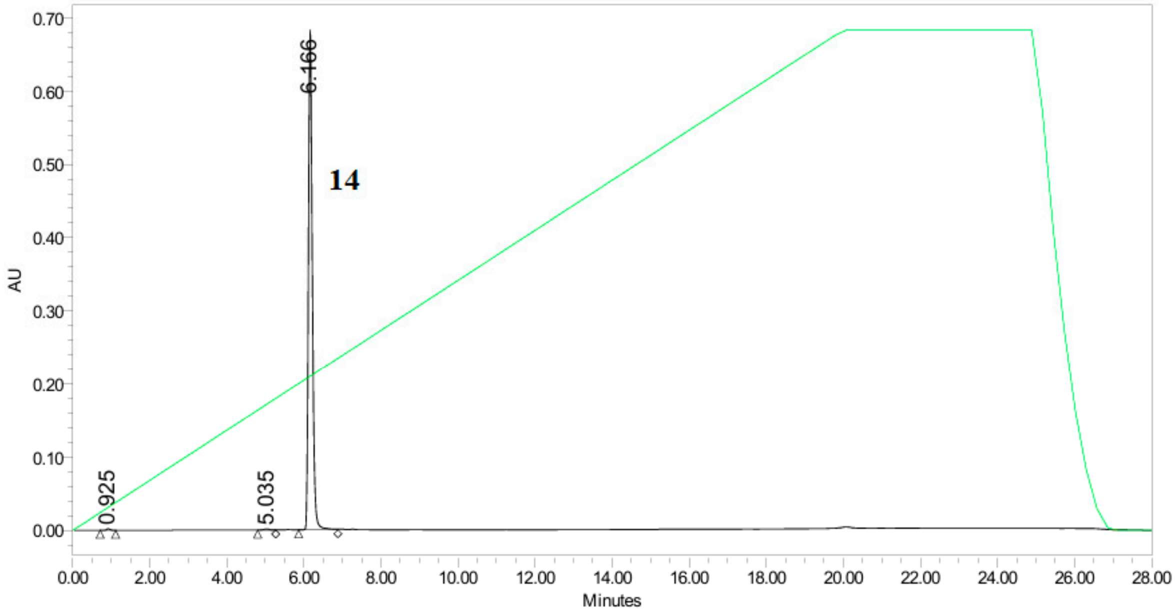

	RT (min)	Area (µV*sec)	% Area	Height (µV)	% Height
1	0.925	11477	0.24	1996	0.29
2	5.035	13967	0.29	1249	0.18
3	6.166	4785734	99.47	679955	99.53

Report Method: Gradient Overlay Report ASC

Page: 1 of 1

Printed: 10.11.2025 13:50:01 Etc/GMT-4

Figure S45. The HPLS data of **14.**

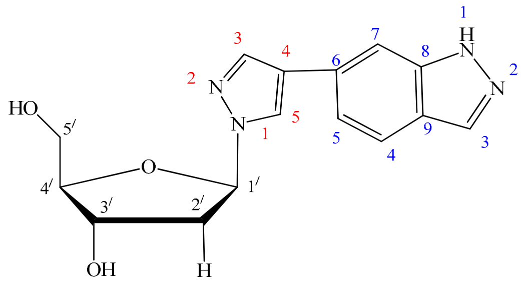

15

N1-(β-D-2'deoxyribofuranosyl)-6-(pyrazol-4-yl)-1H-indazole (15)

^{1}H NMR: δ = 13.01, (s, 1H, N1-H-1 indazole), 8.45 (s, 1H, H-5 pyrazole), 8.04 (s., 1H, H-3 pyrazole), 8.02 (s., 1H, H-3 indazole), 7.74 (d, J = 8.69 Hz, 1H, H-4 indazole), 7.68 (s, 1H, H-7 indazole), 7.38 (dd, J = 8.34 Hz; 1.15 Hz, 1H, H-5 indazole), 6.13 (t, J = 6.39 Hz, 12.66 Hz, 1H, H-1′), 5.24 (s, 1H, OH-3′), 4.85 (s, 1H, OH-5′), 4.40 (s, 1H, 3′), 3.85 (q, J = 5.10 Hz; 13.85 Hz, 1H, H-4′), 3.56 (m, 1H, Ha-5′), 3.46 (m, 1H, Hb-5′), 2.65 (m, 1H, Ha-2′), 2.27 (m, 1H, Hb-2′).

^{13}C NMR: δ = 140.40 (C9 indazole), 137.08 (C3 pyrazole), 133.27 (C3 indazole), 130.05 (C8 indazole), 126.61 (C5 pyrazole), 122.49 (C6 indazole), 121.42 (C4 pyrazole), 120.73 (C4 indazole), 118.85 (C5 indazole), 105.18 (C7 indazole), 89.07 (C1′), 87.66 (C4′), 70,69 (C2′), 70.52 (C3′), 62.02 (C5′), 39.98 (C2′).

^{15}N NMR: δ = 315.72 (N2 indazole), 304.45 (N2 pyrazole), 225.69 (N1 pyrazole), 184.23 (N1 indazole).

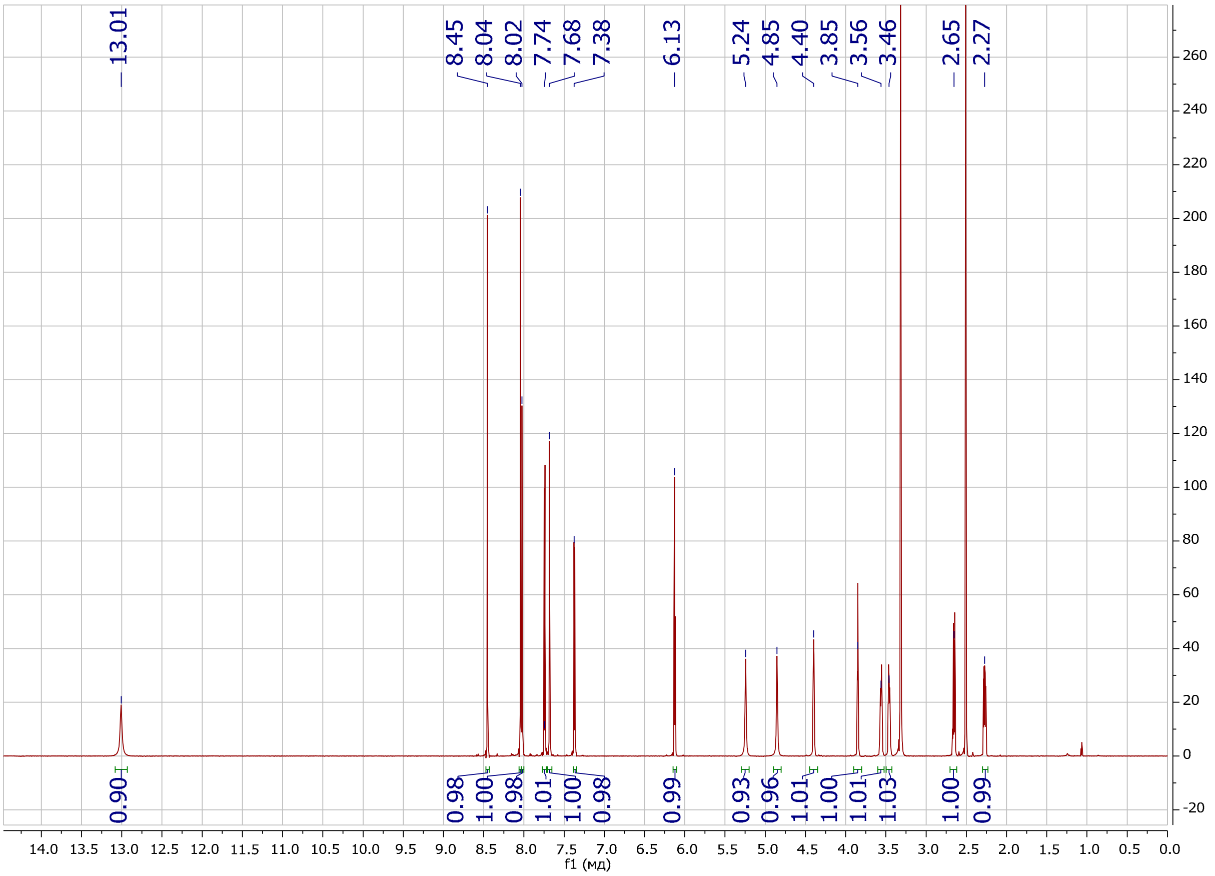

Figure S46. The ^{1}H NMR spectrum of N1-(β-D-2'deoxyribofuranosyl)-6-(pyrazol-4-yl)-1H-indazole (**15**) (700 MHz, DMSO-d_6, 303 K).

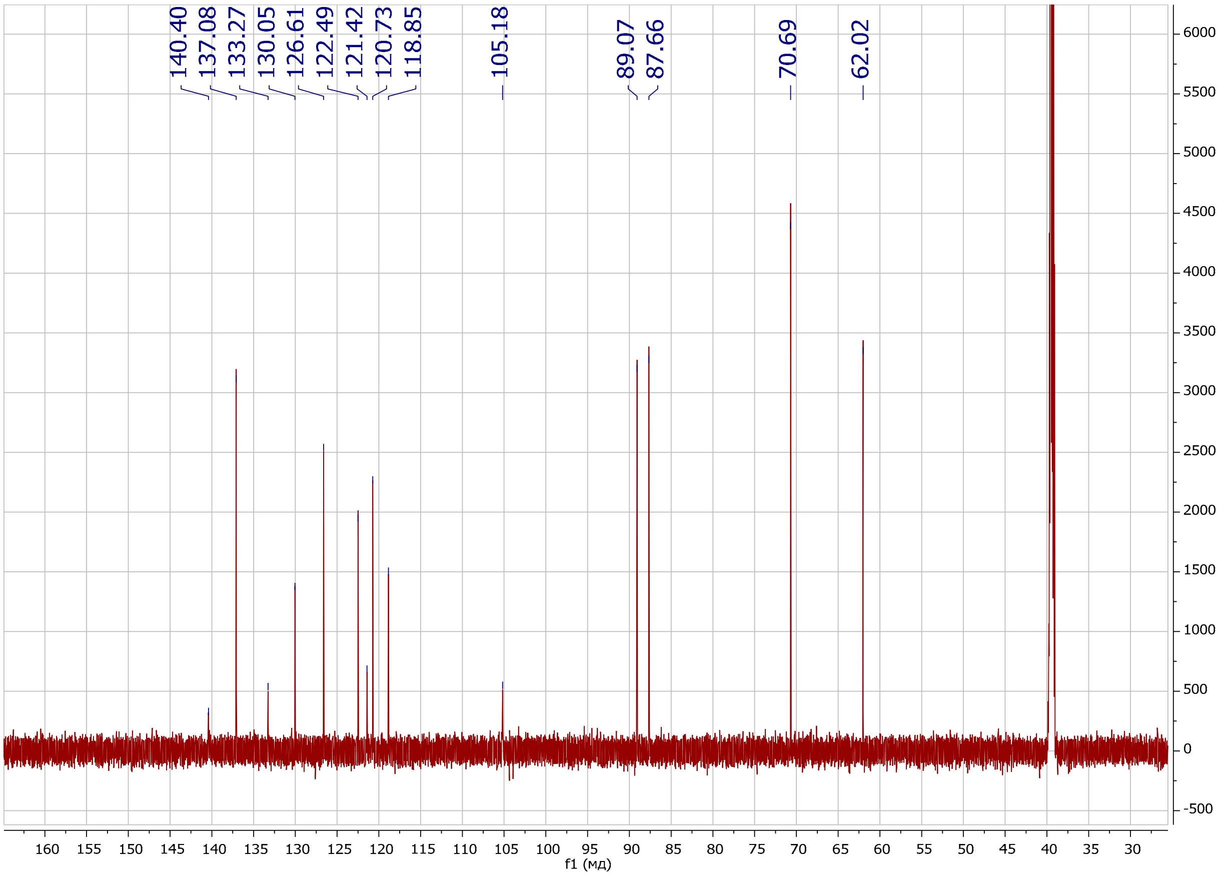

Figure S47. The ^{13}C NMR spectrum of N1-(β-D-2'deoxyribofuranosyl)-6-(pyrazol-4-yl)-1H-indazole (**15**) (176 MHz, DMSO-d_6, 303 K).

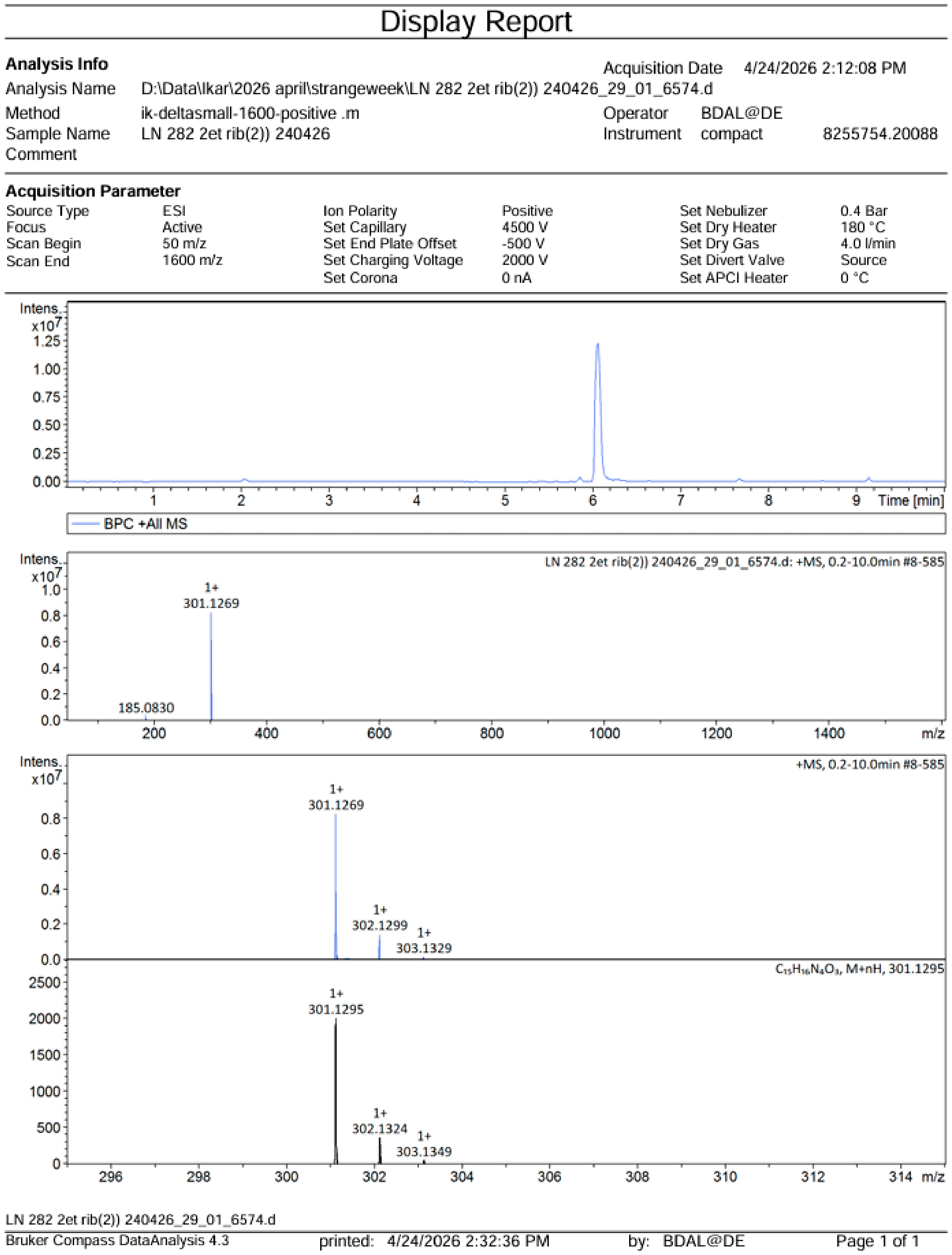

Figure S48. The HRMS data of **15.**

IBCH RAS

Project Name Defaults

Reported by User: Breeze user (Breeze)

Breeze 2 HPLC System

SAMPLE INFORMATION

Sample Name:	LN-282-2dRib purity	Acquired By:	Breeze
Sample Type:	Unknown	Date Acquired:	26.09.2023 16:41:41 GMT-4
Vial:	1	Acq. Method:	100B_dual_280nm_05ml
Injection #:	1	Date Processed:	10.11.2025 13:57:22 GMT-4
Injection Volume:	5.00 ul	Channel Name:	W2489 ChB
Run Time:	20.00 Minutes	Channel Desc.:	W2489 ChB 280nm
Column Type:		Sample Set Name	

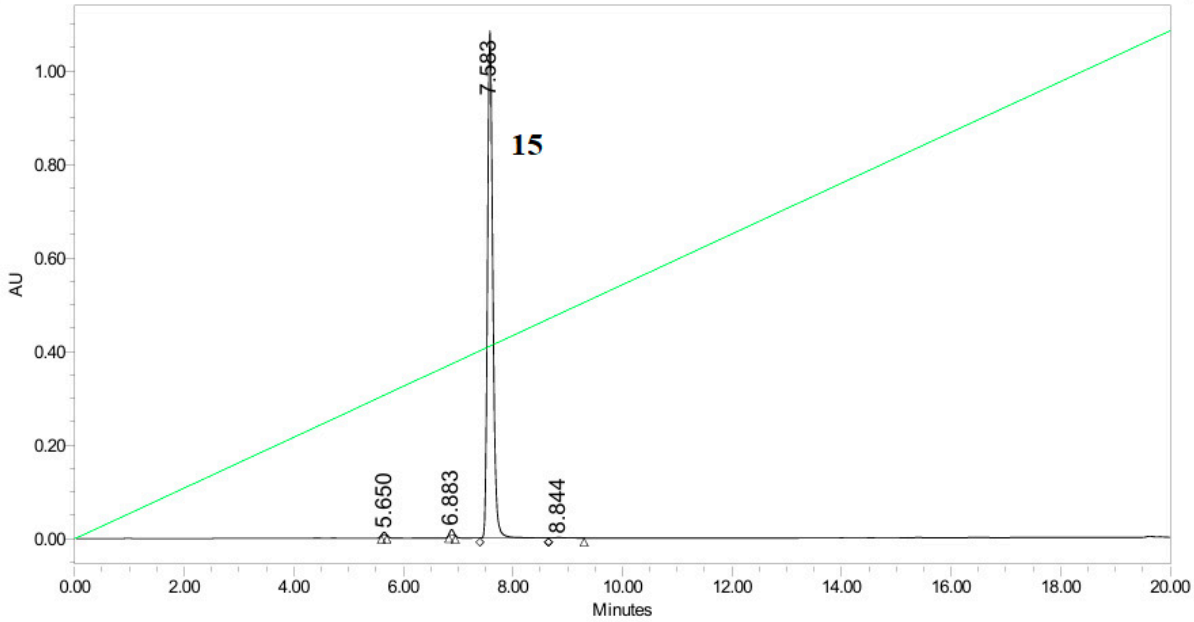

	RT (min)	Area (µV*sec)	% Area	Height (µV)	% Height
1	5.650	22735	0.31	6154	0.56
2	6.883	46346	0.63	11275	1.03
3	7.583	7324282	98.85	1079148	98.32
4	8.844	15814	0.21	1050	0.10

Report Method: Gradient Overlay Report ASC

Printed: 10.11.2025 13:57:56 Etc/GMT-4

Page: 1 of 1

Figure S49. The HPLS data of **15.**

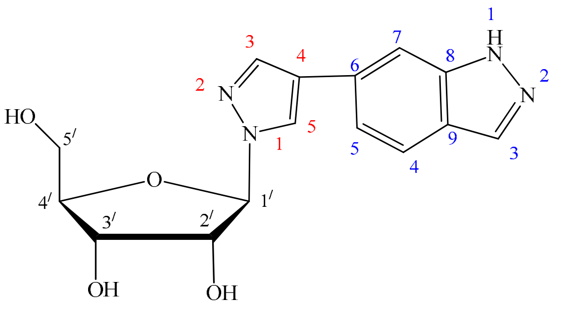

16

N1-(β-D-ribofuranosyl)-6-(pyrazol-4-yl)-1H-indazole (16)

^{1}H NMR: δ = 13.01, (s, 1H, N1-H-1 indazole), 8.50 (s, 1H, H-5 pyrazole), 8.07 (s., 1H, H-3 pyrazole), 8.02 (s., 1H, H-3 indazole), 7.75 (d, J = 8.33 Hz, 1H, H-4 indazole), 7.68 (s, 1H, H-7 indazole), 7.37 (dd, J = 8.54 Hz; 1.25 Hz, 1H, H-5 indazole), 5.69 (d, J = 4.35 Hz, 1H, H-1′), 5.41 (d, J = 5.82 Hz, 1H, OH-2′), 5.10 (d, J = 5.44 Hz, 1H, OH-3′), 4.91 (t, J = 5.77 Hz; 11.43 Hz, 1H, OH-5′), 4.40 (q, J = 4.90 Hz; 10.18 Hz, 1H, H-2′), 4.16 (q, J = 5.28 Hz; 9.63 Hz, 1H, H-3′), 3.93 (q, J = 5.12 Hz; 9.25 Hz, 1H, H-4′), 3.64 (m, 1H, Ha-5′), 3.53 (m, 1H, Hb-5′).

^{13}C NMR: δ = 140.62 (C9 indazole), 137.49 (C3 pyrazole), 133.50 (C3 indazole), 130.23 (C8 indazole), 127.01 (C5 pyrazole), 122.72 (C6 indazole), 121.66 (C4 pyrazole), 120.96 (C4 indazole), 119.03 (C5 indazole), 105.38 (C7 indazole), 93.53 (C1′), 85.14 (C4′), 74.53 (C2′), 70.52 (C3′), 61.86 (C5′).

^{15}N NMR: δ = 315.11 (N2 indazole), 304.83 (N2 pyrazole), 223.50 (N1 pyrazole), 183.23 (N1 indazole).

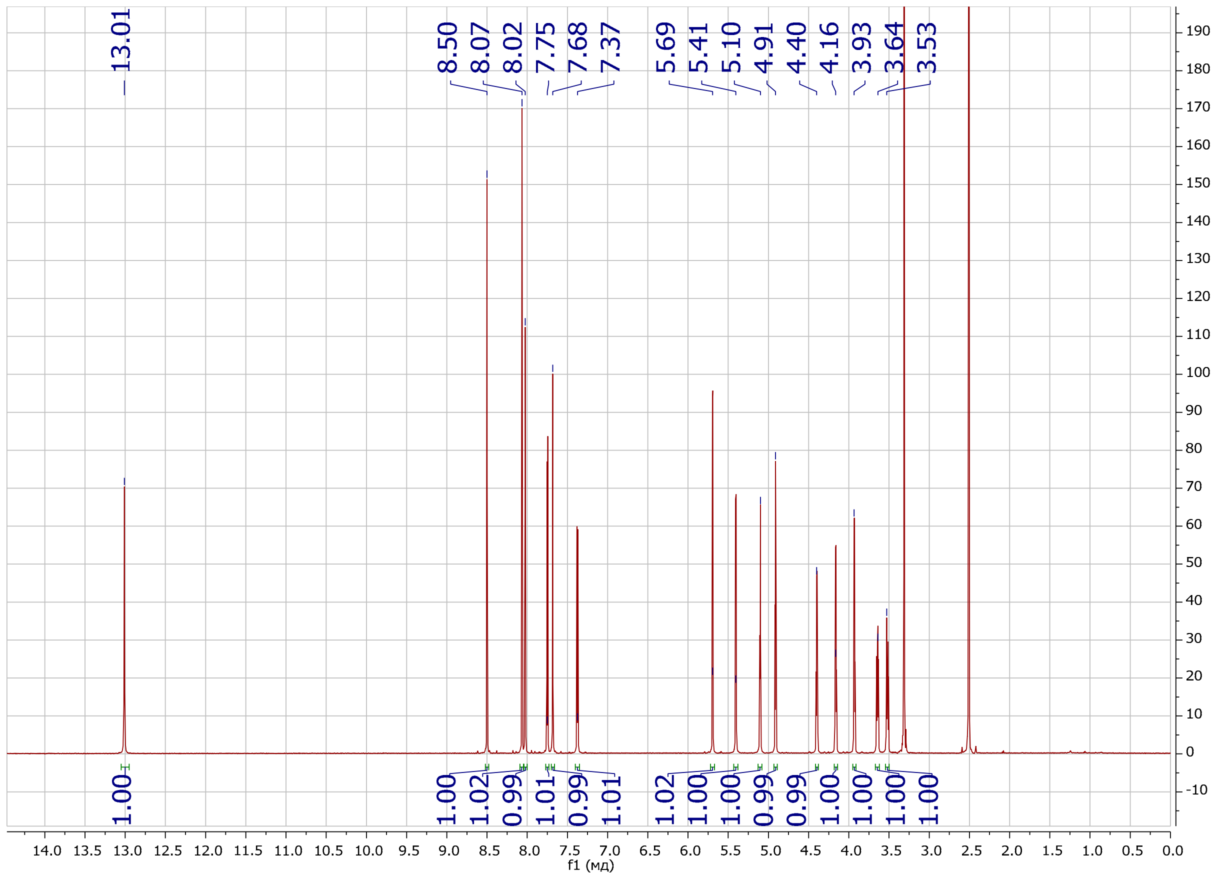

Figure S50. The ^{1}H NMR spectrum of N1-(β-D-ribofuranosyl)-6-(pyrazol-4-yl)-1H-indazole (**16**) (700 MHz, DMSO-d_6, 303 K).

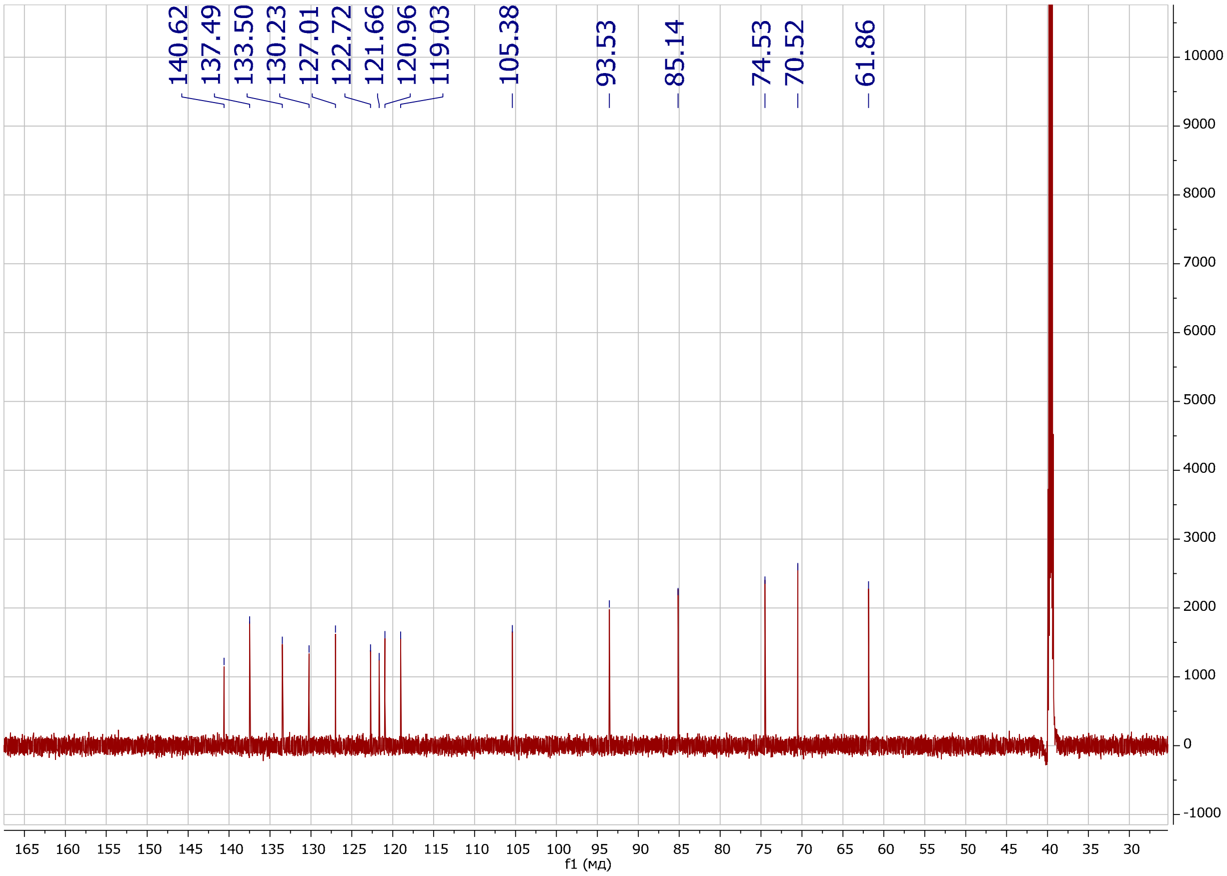

Figure S51. The ^{13}C NMR spectrum of N1-(β-D-ribofuranosyl)-6-(pyrazol-4-yl)-1H-indazole (**16**) (176 MHz, DMSO-d_6, 303 K).

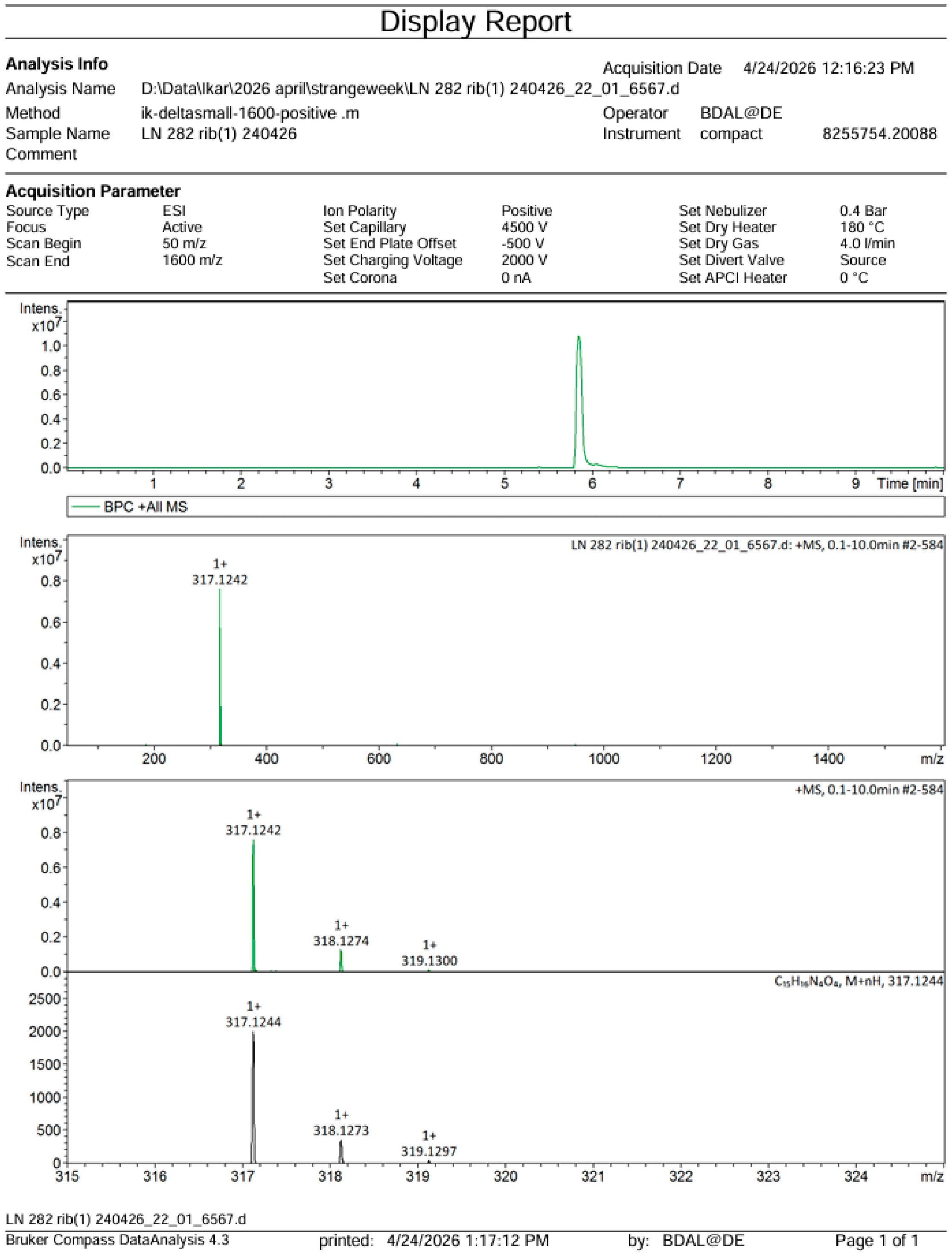

Figure S52. The HMRS data of **16**.

HPLC data of reaction mixtures

IBCH RAS

Project Name Defaults

Reported by User: Breeze user (Breeze)

SAMPLE INFORMATION

Sample Name:	LN-282-Rib purity	Acquired By:	Breeze
Sample Type:	Unknown	Date Acquired:	26.09.2023 10:04:28 GMT-4
Vial:	1	Acq. Method:	100B_dual_280nm_05ml
Injection #:	1	Date Processed:	10.11.2025 14:01:04 GMT-4
Injection Volume:	5.00 ul	Channel Name:	W2489 ChB
Run Time:	30.00 Minutes	Channel Desc.:	W2489 ChB 280nm
Column Type:		Sample Set Name	

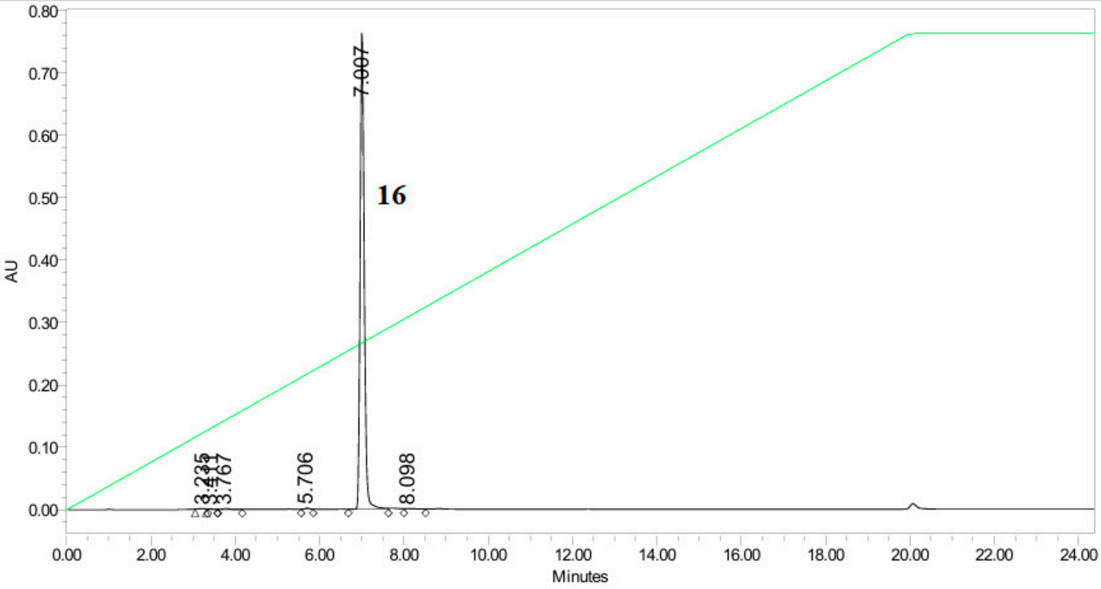

	RT (min)	Area (µV*sec)	% Area	Height (µV)	% Height
1	3.235	7498	0.14	841	0.11
2	3.411	11809	0.22	1286	0.17
3	3.767	26193	0.50	1801	0.23
4	5.706	17498	0.33	2174	0.28
5	7.007	5197075	98.38	766676	99.06
6	8.098	22382	0.42	1204	0.16

Report Method: Gradient Overlay Report ASC

Page: 1 of 1

Printed: 10.11.2025 14:01:30 Etc/GMT-4

Figure S53. The HPLS data of **16.**

HPLC data of reaction mixtures

reachem-pharma

Project Name Sasha

Reported by User: Breeze user (Breeze)

Breeze 2 HPLC System

SAMPLE INFORMATION			
Sample Name:	5-br-indz-2'drib 25h	Acquired By:	Breeze
Sample Type:	Unknown	Date Acquired:	15.04.2025 14:16:44 MSK
Vial:	1	Acq. Method:	
Injection #:	1	Date Processed:	21.10.2025 14:05:17 MSK
Injection Volume:	10.00 ul	Channel Name:	W2489 ChB
Run Time:	30.00 Minutes	Channel Desc.:	W2489 ChB 280nm
Column Type:		Sample Set Name	indz

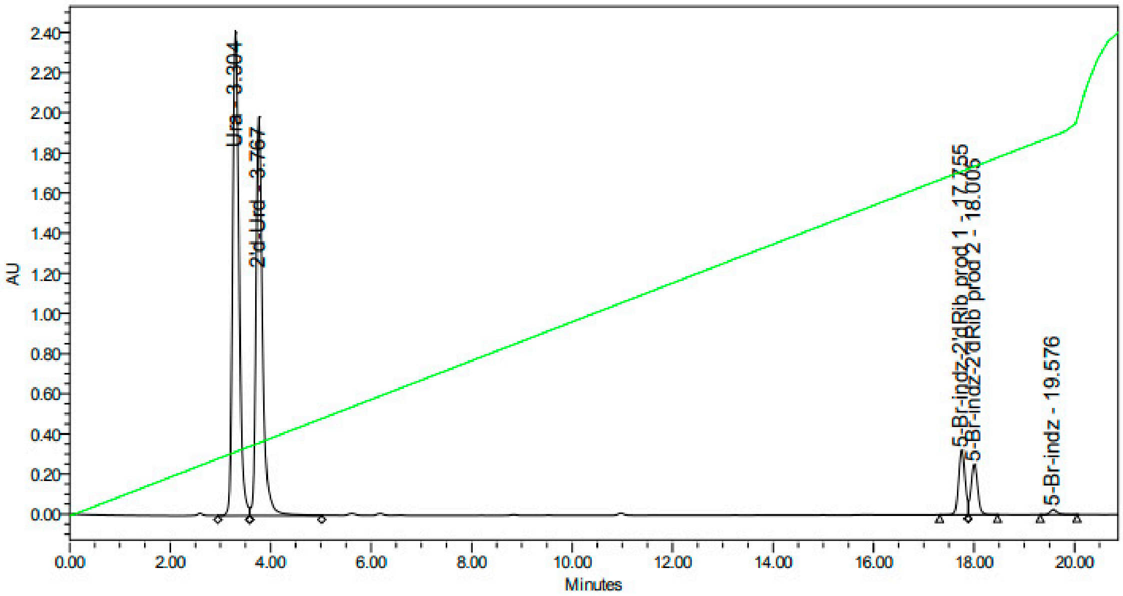

	Peak Name	RT (min)	Area (µV*sec)	% Area	Height (µV)	% Height
1	Ura	3.304	21740302	48.35	2421101	48.41
2	2'd-Urd	3.767	17877014	39.76	1983960	39.67
3	5-Br-indz-2'dRib prod 1	17.755	2820263	6.27	321682	6.43
4	5-Br-indz-2'dRib prod 2	18.005	2273416	5.06	250261	5.00
5	5-Br-indz	19.576	250544	0.56	24282	0.49

Report Method: Gradient Overlay Report ASC

Page: 1 of 1

Printed: 21.10.2025 14:05:48 Europe/Moscow

Figure S54. The chromatogram transglycosylation reaction of the base **1** in 2'-deoxyriboside.

reachem-pharma

Project Name Sasha

Reported by User: Breeze user (Breeze)

SAMPLE INFORMATION

Sample Name:	5-br-indz-rib 5d	Acquired By:	Breeze
Sample Type:	Unknown	Date Acquired:	11.08.2025 10:45:28 MSK
Vial:	1	Acq. Method:	20_100_20min_260_280
Injection #:	1	Date Processed:	21.10.2025 14:00:56 MSK
Injection Volume:	10.00 ul	Channel Name:	W2489 ChB
Run Time:	25.00 Minutes	Channel Desc.:	W2489 ChB 280nm
Column Type:		Sample Set Name	indz

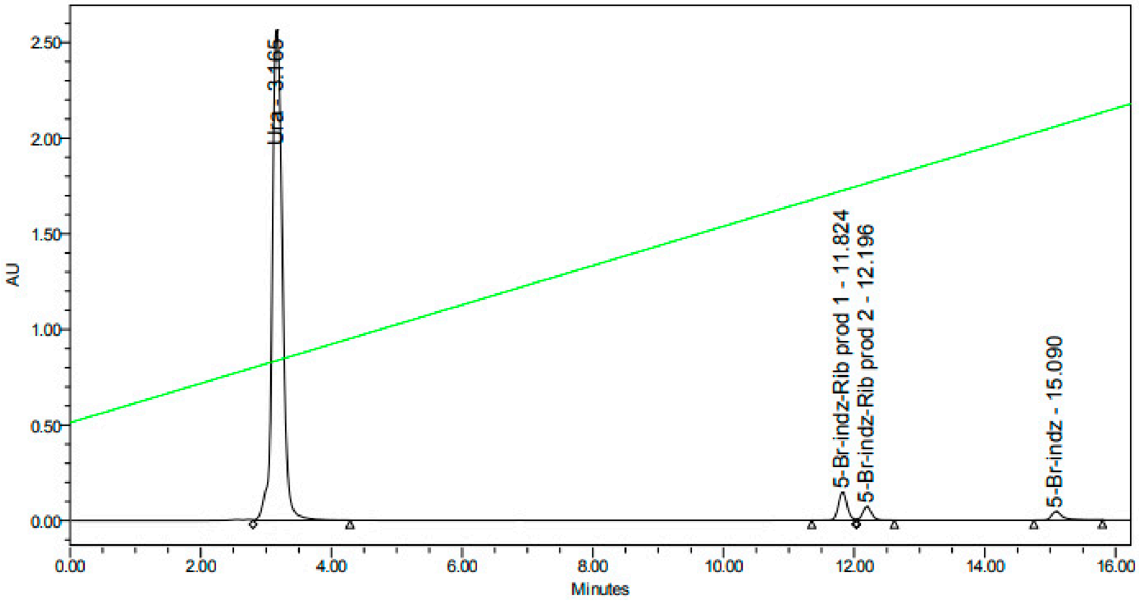

	Peak Name	RT (min)	Area (µV*sec)	% Area	Height (µV)	% Height
1	Ura	3.165	27924730	91.39	2567345	90.65
2	5-Br-indz-Rib prod 1	11.824	1377938	4.51	147088	5.19
3	5-Br-indz-Rib prod 2	12.196	695611	2.28	71983	2.54
4	5-Br-indz	15.090	556574	1.82	45727	1.61

Report Method: Gradient Overlay Report ASC Printed: 21.10.2025 14:01:20 Europe/Moscow

Page: 1 of 1

Figure S55. The chromatogram transglycosylation reaction of the base **1** in riboside.

reachem-pharma
Project Name Sasha
Reported by User: Breeze user (Breeze)

Breeze 2 HPLC System

SAMPLE INFORMATION

Sample Name:	6-br-indz-2'drib 1h	Acquired By:	Breeze
Sample Type:	Unknown	Date Acquired:	11.09.2025 12:17:00 MSK
Vial:	1	Acq. Method:	20_100_20min_260_280
Injection #:	1	Date Processed:	21.10.2025 13:45:18 MSK
Injection Volume:	10.00 ul	Channel Name:	W2489 ChB
Run Time:	25.00 Minutes	Channel Desc.:	W2489 ChB 280nm
Column Type:		Sample Set Name	indz

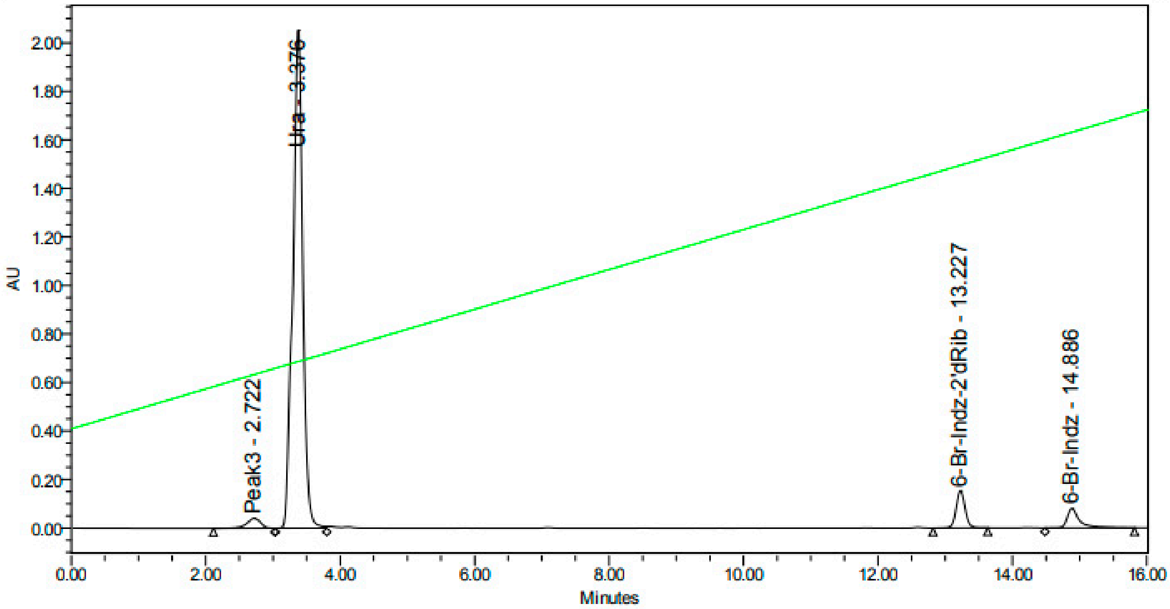

	Peak Name	RT (min)	Area (µV*sec)	% Area	Height (µV)	% Height
1	Peak3	2.722	589133	2.45	40949	1.75
2	Ura	3.376	21067792	87.59	2064311	88.31
3	6-Br-Indz-2'dRib	13.227	1400670	5.82	152653	6.53
4	6-Br-Indz	14.886	995116	4.14	79664	3.41

Report Method: Gradient Overlay Report ASC
Page: 1 of 1

Printed: 21.10.2025
13:46:35 Europe/Moscow

Figure S56. The chromatogram transglycosylation reaction of the base **2** in 2'-deoxyriboside.

reachem-pharma

Project Name Sasha

Reported by User: Breeze user (Breeze)

Breeze 2 HPLC System

SAMPLE INFORMATION

Sample Name:	6-br-indz-rib summ	Acquired By:	Breeze
Sample Type:	Unknown	Date Acquired:	14.08.2025 13:30:40 MSK
Vial:	1	Acq. Method:	20_100_20min_260_280
Injection #:	1	Date Processed:	21.10.2025 13:56:32 MSK
Injection Volume:	10.00 ul	Channel Name:	W2489 ChB
Run Time:	25.00 Minutes	Channel Desc.:	W2489 ChB 280nm
Column Type:		Sample Set Name	indz

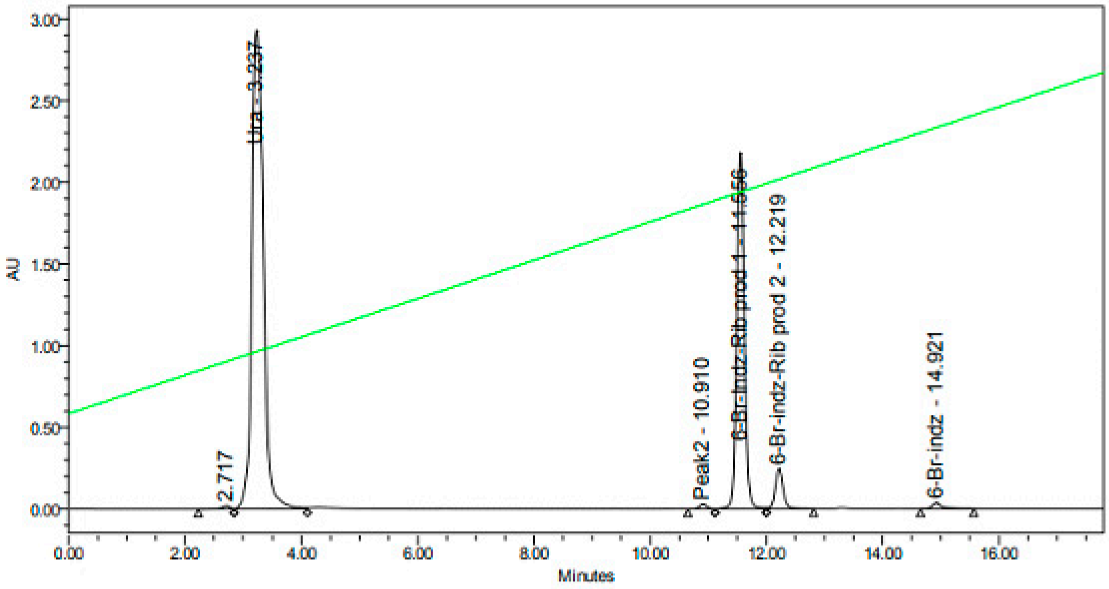

	Peak Name	RT (min)	Area (µV*sec)	% Area	Height (µV)	% Height
1		2.717	212477	0.34	21136	0.39
2	Ura	3.237	40094970	63.90	2932780	53.82
3	Peak2	10.910	262277	0.42	29370	0.54
4	6-Br-Indz-Rib prod 1	11.556	19484802	31.05	2186805	40.13
5	6-Br-indz-Rib prod 2	12.219	2282471	3.64	244101	4.48
6	6-Br-indz	14.921	407364	0.65	35418	0.65

Report Method: Gradient Overlay Report ASC

Printed: 21.10.2025 13:57:28 Europe/Moscow

Page: 1 of 1

Figure S57. The chromatogram transglycosylation reaction of the base **2** in riboside.

IBCH RAS

Project Name Defaults

Reported by User: Breeze user (Breeze)

Breeze 2 HPLC System

SAMPLE INFORMATION

Sample Name:	LN-295-2drib 1h	Acquired By:	Breeze
Sample Type:	Unknown	Date Acquired:	12.12.2023 13:30:13 GMT-4
Vial:	31	Acq. Method:	50B_dual_280_05ml
Injection #:	1	Date Processed:	21.10.2025 15:54:08 GMT-4
Injection Volume:	5.00 ul	Channel Name:	W2489 ChA
Run Time:	18.00 Minutes	Channel Desc.:	W2489 ChA 254nm
Column Type:		Sample Set Name	Fleximers

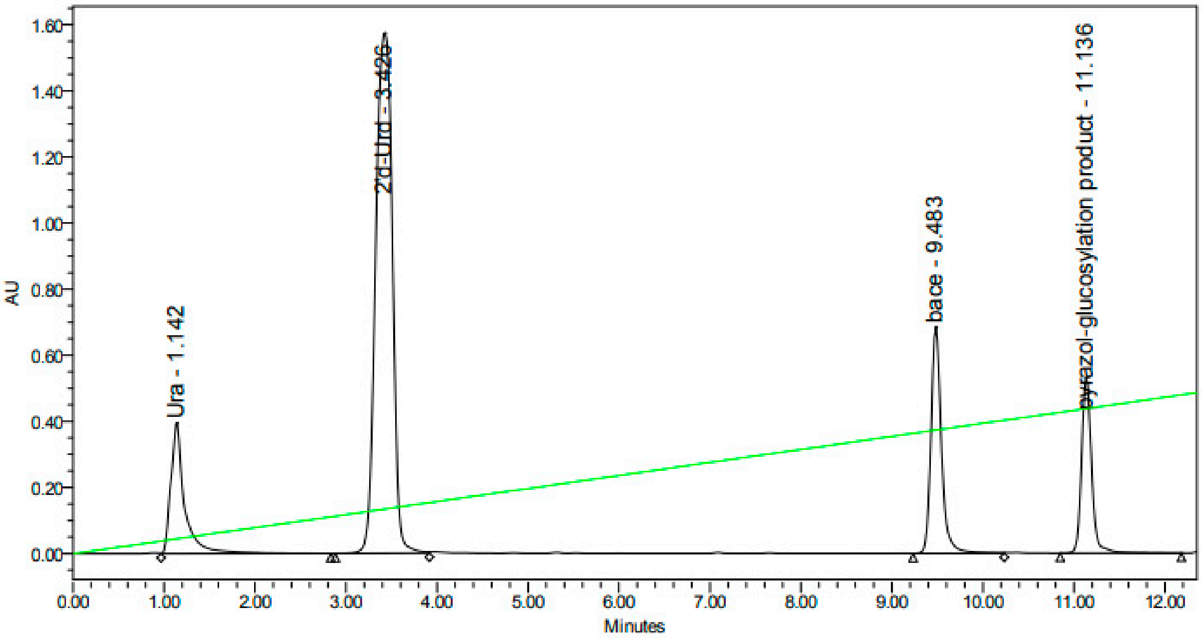

	Peak Name	RT (min)	Area (µV*sec)	% Area	Height (µV)
1	Ura	1.142	4140160	12.27	395824
2	2'd-Urd	3.426	20035610	59.37	1579391
3	bace	9.483	5535696	16.40	683909
4	pyrazol-glucosylation product	11.136	4036036	11.96	532608

Report Method: Gradient Overlay Report ASC

Printed: 21.10.2025 15:54:39 Etc/GMT-4

Page: 1 of 1

Figure S58. The chromatogram transglycosylation reaction of the base **3** in 2'-deoxyriboside.

IBCH RAS

Project Name Defaults

Reported by User: Breeze user (Breeze)

SAMPLE INFORMATION

Sample Name:	LN-295-Rib 25h	Acquired By:	Breeze
Sample Type:	Unknown	Date Acquired:	29.11.2023 15:04:39 GMT-4
Vial:	31	Acq. Method:	30B_dual_280_05ml
Injection #:	1	Date Processed:	21.10.2025 15:59:10 GMT-4
Injection Volume:	5.00 ul	Channel Name:	W2489 ChB
Run Time:	18.00 Minutes	Channel Desc.:	W2489 ChB 280nm
Column Type:		Sample Set Name	Fleximers

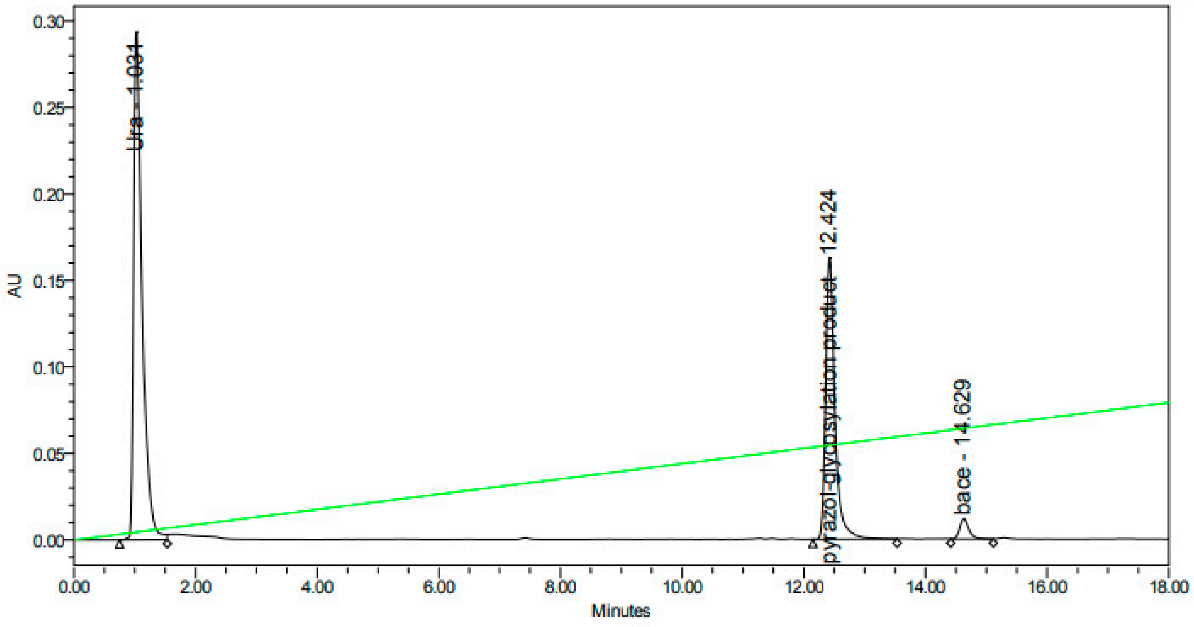

	Peak Name	RT (min)	Area (µV*sec)	% Area	Height (µV)
1	Ura	1.031	2860751	60.82	294677
2	pyrazol-glycosylation product	12.424	1717966	36.53	163914
3	bace	14.629	124784	2.65	11676

Report Method: Gradient Overlay Report ASC

Printed: 21.10.2025 15:59:31 Etc/GMT-4

Page: 1 of 1

Figure S59. The chromatogram transglycosylation reaction of the base **3** in riboside.

IBCH RAS

Project Name Defaults

Reported by User: Breeze user (Breeze)

Breeze 2 HPLC System

SAMPLE INFORMATION

Sample Name:	LN-282-2dRib 1h	Acquired By:	Breeze
Sample Type:	Unknown	Date Acquired:	13.09.2023 12:35:31 GMT-4
Vial:	31	Acq. Method:	50B_dual_280_05ml
Injection #:	1	Date Processed:	21.10.2025 16:18:36 GMT-4
Injection Volume:	5.00 ul	Channel Name:	W2489 ChA
Run Time:	18.00 Minutes	Channel Desc.:	W2489 ChA 254nm
Column Type:		Sample Set Name	Fleximers

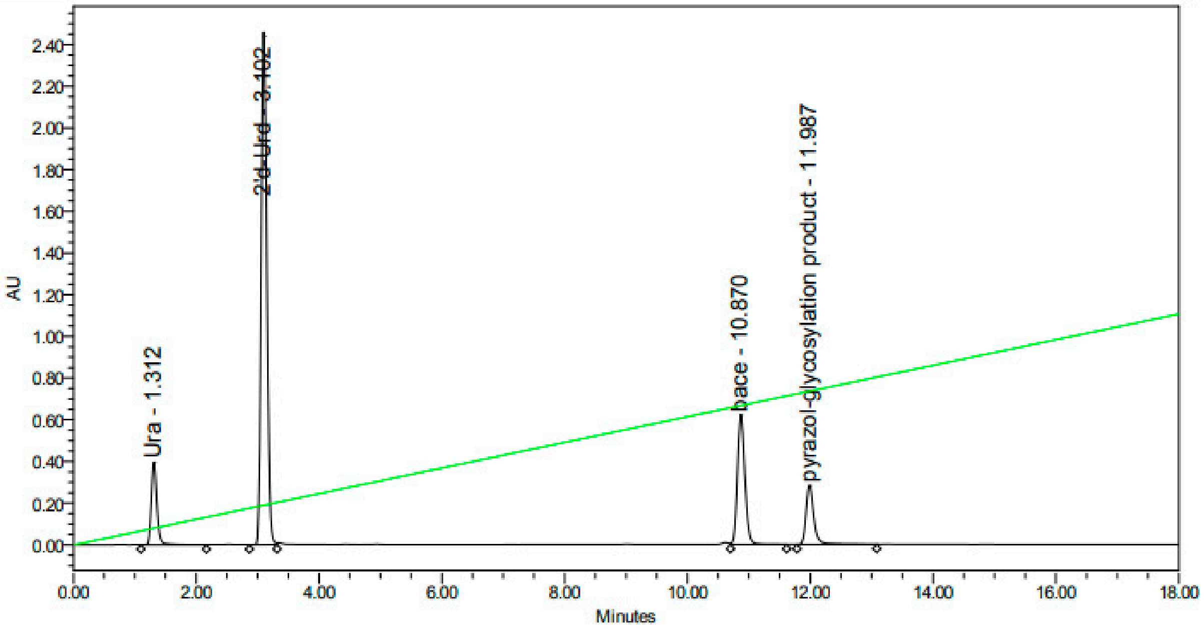

	Peak Name	RT (min)	Area (µV*sec)	% Area	Height (µV)
1	Ura	1.312	2524657	9.81	396809
2	2'd-Urd	3.102	15368359	59.74	2448915
3	bace	10.870	5287341	20.55	623960
4	pyrazol-glycosylation product	11.987	2544224	9.89	284173

Report Method: Gradient Overlay Report ASC   Printed: 21.10.2025

Page: 1 of 1   16:19:03 Etc/GMT-4

Figure S60. The chromatogram transglycosylation reaction of the base **4** in 2'-deoxyriboside.

IBCH RAS

Project Name Defaults

Reported by User: Breeze user (Breeze)

SAMPLE INFORMATION

Sample Name:	LN-282-Rib 21h	Acquired By:	Breeze
Sample Type:	Unknown	Date Acquired:	13.09.2023 11:30:20 GMT-4
Vial:	31	Acq. Method:	50B_dual_280_05ml
Injection #:	1	Date Processed:	21.10.2025 16:10:14 GMT-4
Injection Volume:	5.00 ul	Channel Name:	W2489 ChB
Run Time:	18.00 Minutes	Channel Desc.:	W2489 ChB 280nm
Column Type:		Sample Set Name	Fleximers

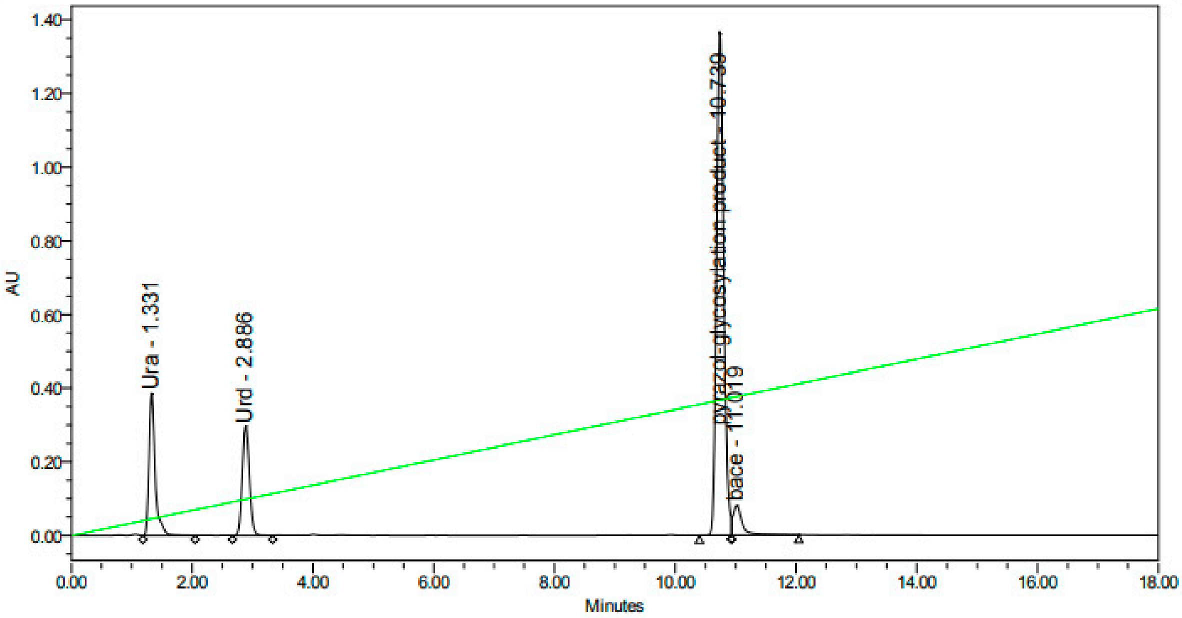

	Peak Name	RT (min)	Area (µV*sec)	% Area	Height (µV)
1	Ura	1.331	2774217	15.92	385121
2	Urd	2.886	2428442	13.94	296979
3	pyrazol-glycosylation product	10.739	11358637	65.18	1372903
4	bace	11.019	864873	4.96	81044

Report Method: Gradient Overlay Report ASC

Page: 1 of 1

Printed: 21.10.2025 16:10:34 Etc/GMT-4

Figure S61. The chromatogram transglycosylation reaction of the base **4** in riboside.

IBCH RAS

Project Name Fleximer

Reported by User: Breeze user (Breeze)

SAMPLE INFORMATION

Sample Name:	LN-270-2'dr syn 1h	Acquired By:	Breeze
Sample Type:	Unknown	Date Acquired:	17.07.2025 16:17:01 GMT-4
Vial:	1	Acq. Method:	30B_dual_280_05ml
Injection #:	1	Date Processed:	21.10.2025 15:17:32 GMT-4
Injection Volume:	10.00 ul	Channel Name:	W2489 ChB
Run Time:	25.00 Minutes	Channel Desc.:	W2489 ChB 280nm
Column Type:		Sample Set Name	g

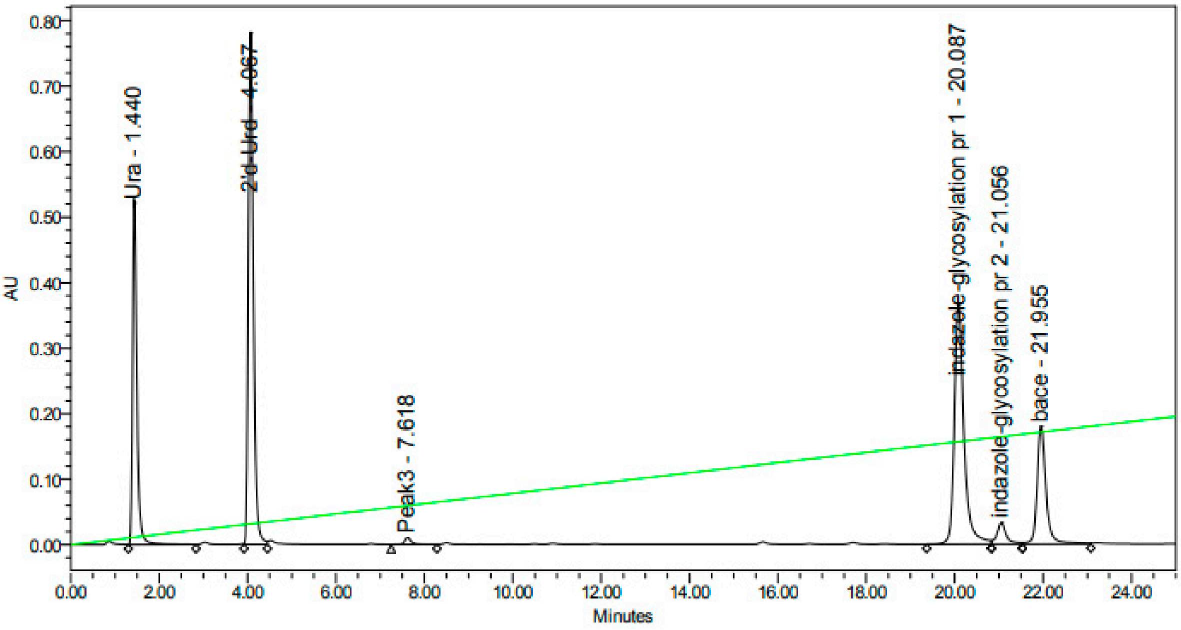

	Peak Name	RT (min)	Area (µV*sec)	% Area	Height (µV)	% Height
1	Ura	1.440	3636337	19.67	528811	27.81
2	2'd-Urd	4.067	6147538	33.25	780052	41.03
3	Peak3	7.618	110459	0.60	10632	0.56
4	indazole-glycosylation pr 1	20.087	5573974	30.15	368469	19.38
5	indazole-glycosylation pr 2	21.056	485023	2.62	33155	1.74
6	bace	21.955	2534167	13.71	180260	9.48

Report Method: Gradient Overlay Report ASC
Page: 1 of 2

Printed: 21.10.2025 15:48:41 Etc/GMT-4

Figure S62. The chromatogram transglycosylation reaction of the base **5** in 2'-deoxyriboside.

IBCH RAS

Project Name Fleximer

Reported by User: Breeze user (Breeze)

SAMPLE INFORMATION

Sample Name:	In-270-rib 20d syn	Acquired By:	Breeze
Sample Type:	Unknown	Date Acquired:	20.05.2025 11:41:22 GMT-4
Vial:	1	Acq. Method:	50B_dual_280_05ml
Injection #:	1	Date Processed:	21.10.2025 14:37:14 GMT-4
Injection Volume:	10.00 ul	Channel Name:	W2489 ChB
Run Time:	30.00 Minutes	Channel Desc.:	W2489 ChB 280nm
Column Type:		Sample Set Name	270

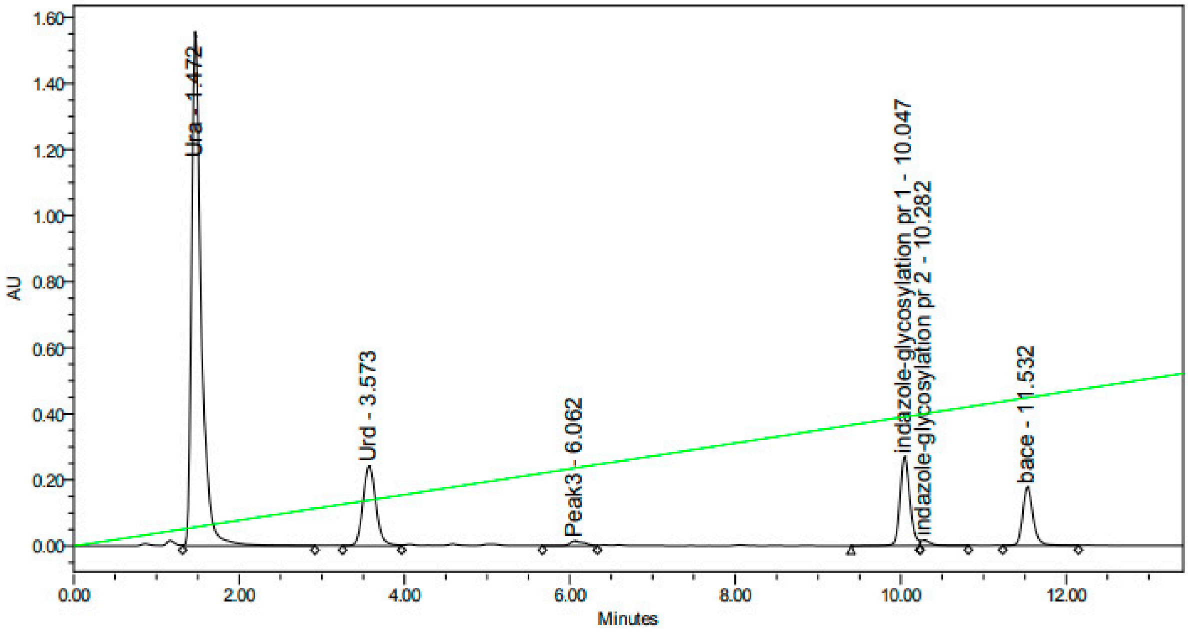

	Peak Name	RT (min)	Area (µV*sec)	% Area	Height (µV)
1	Ura	1.472	12663983	64.99	1560716
2	Urd	3.573	2658005	13.64	242066
3	Peak3	6.062	183072	0.94	14579
4	indazole-glycosylation pr 1	10.047	2234919	11.47	270764
5	indazole-glycosylation pr 2	10.282	155930	0.80	16714
6	bace	11.532	1590839	8.16	177594

Report Method: Gradient Overlay Report ASC

Printed: 21.10.2025 14:38:51 Etc/GMT-4

Page: 1 of 2

Figure S63. The chromatogram transglycosylation reaction of the base **5** in riboside.

IBCH RAS

Project Name Defaults

Reported by User: Breeze user (Breeze)

Breeze 2 HPLC System

SAMPLE INFORMATION

Sample Name:	FL 28/1 1 h	Acquired By:	Breeze
Sample Type:	Unknown	Date Acquired:	25.01.2023 16:07:00 GMT-4
Vial:	31	Acq. Method:	50B_dual_280_05ml
Injection #:	1	Date Processed:	10.11.2025 14:09:17 GMT-4
Injection Volume:	10.00 ul	Channel Name:	W2489 ChA
Run Time:	18.00 Minutes	Channel Desc.:	W2489 ChA 254nm
Column Type:		Sample Set Name	Fleximers

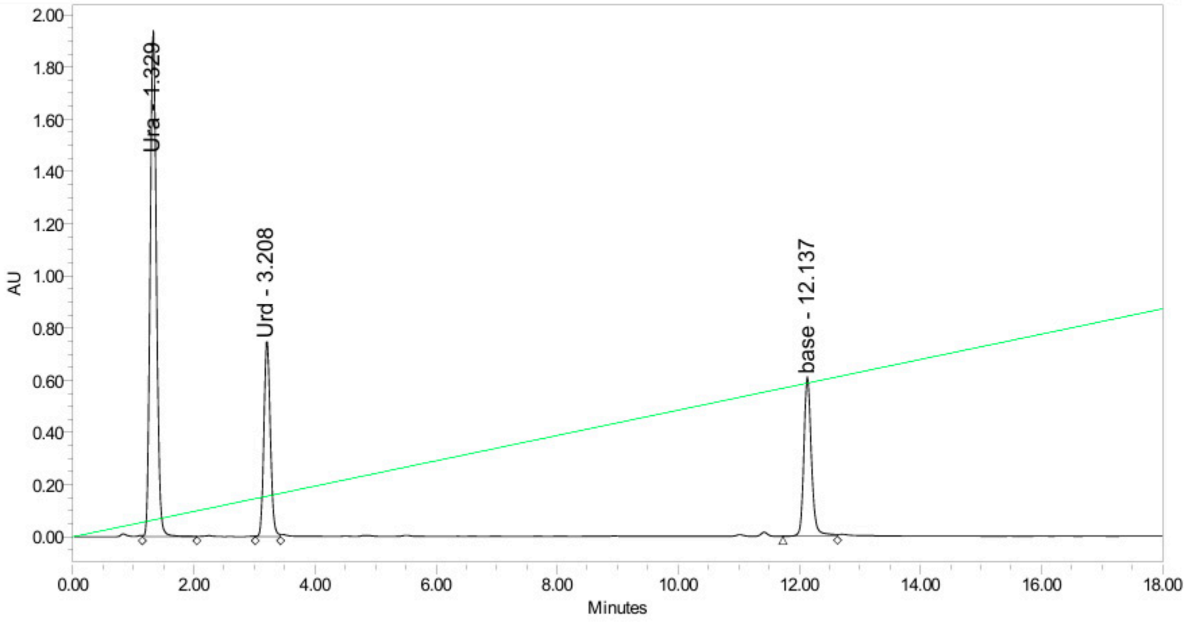

	Peak Name	RT (min)	Area (µV*sec)	% Area	Height (µV)	% Height
1	Ura	1.329	14085605	56.49	1940497	58.71
2	Urd	3.208	5527945	22.17	753324	22.79
3	base	12.137	5321000	21.34	611544	18.50

Report Method: Gradient Overlay Report ASC

Page: 1 of 1

Printed: 10.11.2025 14:09:41 Etc/GMT-4

Figure S64. The chromatogram transglycosylation reaction of the base **6** in riboside (not a substrate)

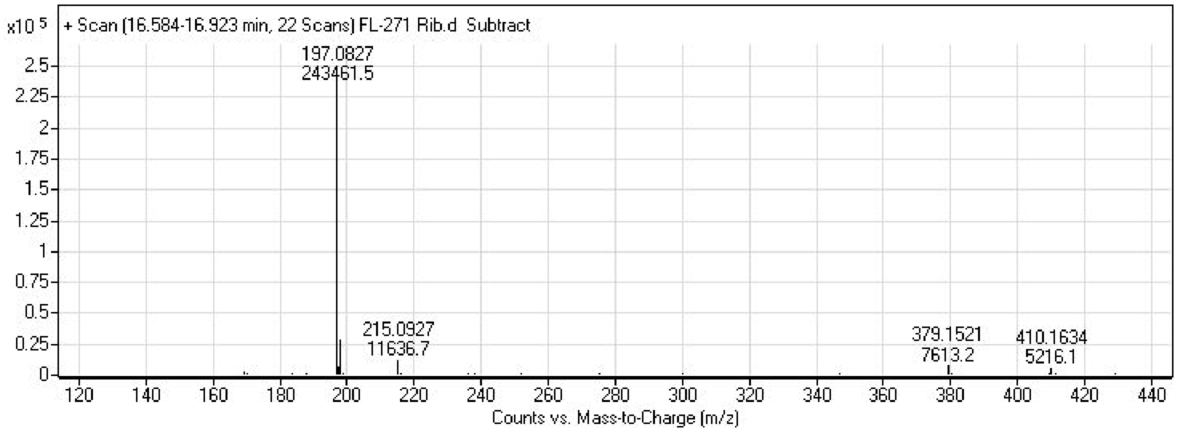

Figure S65. The mass-spectrometry data of transglycosylation reaction of the base **6** (not a substrate)

ADA *E. coli* inhibition data

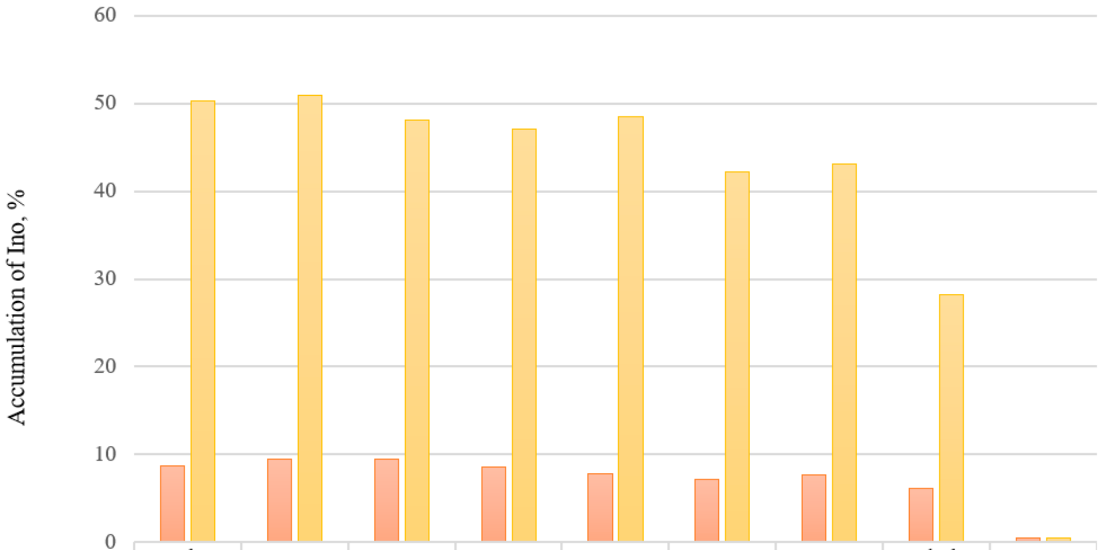

	ado	1	2	3	4	5	6	clad	pent
5 min	8.66	9.45	9.51	8.59	7.84	7.16	7.69	6.15	0.51
25 min	50.25	50.97	48.07	47.07	48.42	42.25	43.07	28.24	0.51

Figure S66. Comparative analysis of the rate of adenosine deamination under the action of ADA *E. coli* for 5 and 25 minutes in the presence of bases **1-6**.

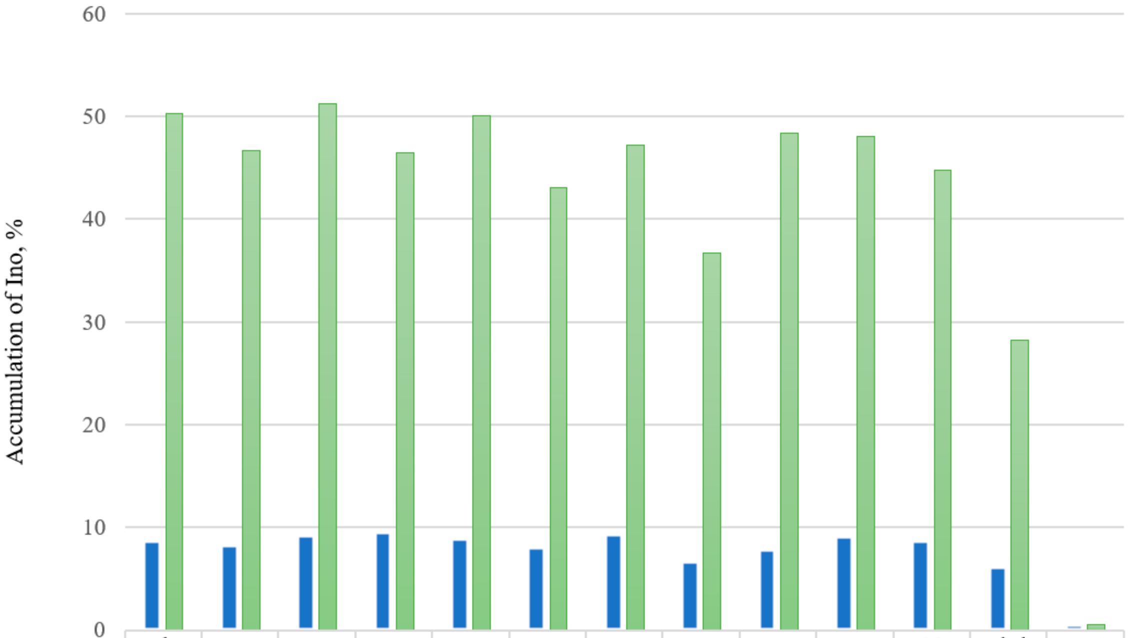

	ado	7	8	9	10	11	12	13	14	15	16	clad	pent
5 min	8.66	8.24	9.19	9.55	8.92	8.09	9.32	6.74	7.84	9.09	8.75	6.15	0.49
25min	50.25	46.66	51.28	46.49	50.02	43.04	47.18	36.72	48.42	48.08	44.81	28.24	0.50

Figure S67. Comparative analysis of the rate of adenosine deamination under the action of ADA *E. coli* for 5 and 25 minutes in the presence of nucleosides **7-16**.
